# Genome-Wide Association and Functional Follow-Up Reveals New Loci for Kidney Function

**DOI:** 10.1371/journal.pgen.1002584

**Published:** 2012-03-29

**Authors:** Cristian Pattaro, Anna Köttgen, Alexander Teumer, Maija Garnaas, Carsten A. Böger, Christian Fuchsberger, Matthias Olden, Ming-Huei Chen, Adrienne Tin, Daniel Taliun, Man Li, Xiaoyi Gao, Mathias Gorski, Qiong Yang, Claudia Hundertmark, Meredith C. Foster, Conall M. O'Seaghdha, Nicole Glazer, Aaron Isaacs, Ching-Ti Liu, Albert V. Smith, Jeffrey R. O'Connell, Maksim Struchalin, Toshiko Tanaka, Guo Li, Andrew D. Johnson, Hinco J. Gierman, Mary Feitosa, Shih-Jen Hwang, Elizabeth J. Atkinson, Kurt Lohman, Marilyn C. Cornelis, Åsa Johansson, Anke Tönjes, Abbas Dehghan, Vincent Chouraki, Elizabeth G. Holliday, Rossella Sorice, Zoltan Kutalik, Terho Lehtimäki, Tõnu Esko, Harshal Deshmukh, Sheila Ulivi, Audrey Y. Chu, Federico Murgia, Stella Trompet, Medea Imboden, Barbara Kollerits, Giorgio Pistis, CARDIoGRAM Consortium, ICBP Consortium, CARe Consortium, Wellcome Trust Case Control Consortium 2 (WTCCC2), Tamara B. Harris, Lenore J. Launer, Thor Aspelund, Gudny Eiriksdottir, Braxton D. Mitchell, Eric Boerwinkle, Helena Schmidt, Margherita Cavalieri, Madhumathi Rao, Frank B. Hu, Ayse Demirkan, Ben A. Oostra, Mariza de Andrade, Stephen T. Turner, Jingzhong Ding, Jeanette S. Andrews, Barry I. Freedman, Wolfgang Koenig, Thomas Illig, Angela Döring, H.-Erich Wichmann, Ivana Kolcic, Tatijana Zemunik, Mladen Boban, Cosetta Minelli, Heather E. Wheeler, Wilmar Igl, Ghazal Zaboli, Sarah H. Wild, Alan F. Wright, Harry Campbell, David Ellinghaus, Ute Nöthlings, Gunnar Jacobs, Reiner Biffar, Karlhans Endlich, Florian Ernst, Georg Homuth, Heyo K. Kroemer, Matthias Nauck, Sylvia Stracke, Uwe Völker, Henry Völzke, Peter Kovacs, Michael Stumvoll, Reedik Mägi, Albert Hofman, Andre G. Uitterlinden, Fernando Rivadeneira, Yurii S. Aulchenko, Ozren Polasek, Nick Hastie, Veronique Vitart, Catherine Helmer, Jie Jin Wang, Daniela Ruggiero, Sven Bergmann, Mika Kähönen, Jorma Viikari, Tiit Nikopensius, Michael Province, Shamika Ketkar, Helen Colhoun, Alex Doney, Antonietta Robino, Franco Giulianini, Bernhard K. Krämer, Laura Portas, Ian Ford, Brendan M. Buckley, Martin Adam, Gian-Andri Thun, Bernhard Paulweber, Margot Haun, Cinzia Sala, Marie Metzger, Paul Mitchell, Marina Ciullo, Stuart K. Kim, Peter Vollenweider, Olli Raitakari, Andres Metspalu, Colin Palmer, Paolo Gasparini, Mario Pirastu, J. Wouter Jukema, Nicole M. Probst-Hensch, Florian Kronenberg, Daniela Toniolo, Vilmundur Gudnason, Alan R. Shuldiner, Josef Coresh, Reinhold Schmidt, Luigi Ferrucci, David S. Siscovick, Cornelia M. van Duijn, Ingrid Borecki, Sharon L. R. Kardia, Yongmei Liu, Gary C. Curhan, Igor Rudan, Ulf Gyllensten, James F. Wilson, Andre Franke, Peter P. Pramstaller, Rainer Rettig, Inga Prokopenko, Jacqueline C. M. Witteman, Caroline Hayward, Paul Ridker, Afshin Parsa, Murielle Bochud, Iris M. Heid, Wolfram Goessling, Daniel I. Chasman, W. H. Linda Kao, Caroline S. Fox

**Affiliations:** 1 Institute of Genetic Medicine, European Academy of Bozen/Bolzano (EURAC) and Affiliated Institute of the University of Lübeck, Bolzano, Italy; 2 Department of Epidemiology, Johns Hopkins Bloomberg School of Public Health, Baltimore, Maryland, United States of America; 3 Renal Division, Freiburg University Clinic, Freiburg, Germany; 4 Interfaculty Institute for Genetics and Functional Genomics, University of Greifswald, Greifswald, Germany; 5 Division of Genetics, Department of Medicine, Brigham and Women's Hospital, Harvard Medical School, Boston, Massachusetts, United States of America; 6 Department of Internal Medicine II, University Medical Center Regensburg, Regensburg, Germany; 7 Center for Statistical Genetics, Department of Biostatistics, University of Michigan, Ann Arbor, Michigan, United States of America; 8 Department of Internal Medicine II, University Hospital Regensburg, Regensburg, Germany; 9 Department of Epidemiology and Preventive Medicine, Regensburg University Medical Center, Regensburg, Germany; 10 Department of Neurology, Boston University School of Medicine, Boston, Massachusetts, United States of America; 11 Department of Biostatistics, Boston University School of Public Health, Boston, Massachusetts, United States of America; 12 Division of Statistical Genomics, Washington University School of Medicine, St. Louis, Missouri, United States of America; 13 Department of Epidemiology and Preventive Medicine, University Hospital Regensburg, Regensburg, Germany; 14 Institute of Epidemiology I, Helmholtz Zentrum München, German Research Center for Environmental Health, Neuherberg, Germany; 15 Department of Biostatistics, Boston University School of Public Health, Boston, Massachusetts, United States of America; 16 Renal Division, Freiburg University Clinic, Freiburg, Germany; 17 National Heart, Lung, and Blood Institute's Framingham Heart Study and the Center for Population Studies, Framingham, Massachusetts, United States of America; 18 Division of Nephrology, Brigham and Women's Hospital and Harvard Medical School, Boston, Massachusetts, United States of America; 19 Section of Preventive Medicine and Epidemiology, Department of Medicine, Boston University School of Medicine, Boston, Massachusetts, United States of America; 20 Genetic Epidemiology Unit, Department of Epidemiology, Erasmus University Medical Center, Rotterdam, The Netherlands; 21 Centre for Medical Systems Biology, Leiden, The Netherlands; 22 Department of Biostatistics, Boston University School of Public Health, Boston, Massachusetts, United States of America; 23 Icelandic Heart Association, Research Institute, Kopavogur, Iceland; 24 University of Iceland, Reykjavik, Iceland; 25 Department of Medicine, University of Maryland Medical School, Baltimore, Maryland, United States of America; 26 Department of Epidemiology and Biostatistics and Department of Forensic Molecular Biology, Erasmus University Medical Centre, Rotterdam, The Netherlands; 27 Clinical Research Branch, National Institute of Aging, Baltimore, Maryland, United States of America; 28 University of Washington, Seattle, Washington, United States of America; 29 Department of Developmental Biology, Stanford University, Stanford, California, United States of America; 30 Division of Biomedical Statistics and Informatics, Mayo Clinic, Rochester, Minnesota, United States of America; 31 Department of Epidemiology and Prevention, Public Health Sciences, Wake Forest School of Medicine, Winston-Salem, North Carolina, United States of America; 32 Department of Nutrition, Harvard School of Public Health, Boston, Massachusetts, United States of America; 33 Genetics and Pathology, Rudbeck Laboratory, Uppsala University, Uppsala, Sweden; 34 Department of Medicine, University of Leipzig, Leipzig, Germany; 35 IFB Adiposity Diseases, University of Leipzig, Leipzig, Germany; 36 Department of Epidemiology, Erasmus University Medical Center, Rotterdam, The Netherlands; 37 Inserm UMR744, Institut Pasteur, Lille, France; 38 Centre for Clinical Epidemiology and Biostatistics, School of Medicine and Public Health, University of Newcastle, Newcastle, Australia; 39 Centre for Information-based Medicine, Hunter Medical Research Institute, Newcastle, Australia; 40 Institute of Genetics and Biophysics “Adriano-Buzzati Traverso”–CNR, Napoli, Italy; 41 Department of Medical Genetics, University of Lausanne, Lausanne, Switzerland; 42 Swiss Institute of Bioinformatics, Lausanne, Switzerland; 43 Department of Clinical Chemistry, University of Tampere and Tampere University Hospital, Centre for Laboratory Medicine Tampere Finn-Medi 2, Tampere, Finland; 44 Estonian Genome Center of University of Tartu (EGCUT), Tartu, Estonia; 45 Estonian Biocenter and Institute of Molecular and Cell Biology, University of Tartu, Tartu, Estonia; 46 Wellcome Trust Centre for Molecular Medicine, Clinical Research Centre, Ninewells Hospital, University of Dundee, Dundee, United Kingdom; 47 Institute for Maternal and Child Health – IRCCS “Burlo Garofolo”, Trieste, Italy; 48 Brigham and Women's Hospital, Boston, Massachusetts, United States of America; 49 Institute of Population Genetics – CNR, Sassari, Italy; 50 Department of Cardiology, Leiden University Medical Center, Leiden, The Netherlands; 51 Unit of Chronic Disease Epidemiology, Swiss Tropical and Public Health Institute, Basel, Switzerland; 52 Division of Genetic Epidemiology, Innsbruck Medical University, Innsbruck, Austria; 53 Division of Genetics and Cell Biology, San Raffaele Scientific Institute, Milano, Italy; 54 Laboratory of Epidemiology, Demography, and Biometry, NIA, Bethesda, Maryland, United States of America; 55 Human Genetics Center, University of Texas Health Science Center, Houston, Texas, United States of America; 56 Austrian Stroke Prevention Study, Institute of Molecular Biology and Biochemistry and Department of Neurology, Medical University Graz, Graz, Austria; 57 Austrian Stroke Prevention Study, University Clinic of Neurology, Department of Special Neurology, Medical University Graz, Graz, Austria; 58 Division of Nephrology/Tufts Evidence Practice Center, Tufts University School of Medicine, Tufts Medical Center, Boston, Massachusetts, United States of America; 59 Department of Internal Medicine, Division of Nephrology and Hypertension, Mayo Clinic, Rochester, Minnesota, United States of America; 60 Department of Internal Medicine/Geriatrics, Wake Forest School of Medicine, Winston-Salem, North Carolina, United States of America; 61 Department of Biostatistical Sciences, Public Health Sciences, Wake Forest School of Medicine, Winston-Salem, North Carolina, United States of America; 62 Department of Internal Medicine, Wake Forest School of Medicine, Winston-Salem, North Carolina, United States of America; 63 Abteilung Innere II, Universitätsklinikum Ulm, Ulm, Germany; 64 Institute of Epidemiology II, Helmholtz Zentrum München, German Research Center for Environmental Health, Neuherberg, Germany; 65 Institute of Medical Informatics, Biometry, and Epidemiology, Ludwig-Maximilians-Universität, Munich, Germany; 66 Klinikum Grosshadern, Neuherberg, Germany; 67 Croatian Centre for Global Health, University of Split Medical School, Split, Croatia; 68 Department of Genetics, Stanford University, Stanford, California, United States of America; 69 Department of Medicine, University of Chicago, Chicago, Illinois, United States of America; 70 Center for Population Health Sciences, University of Edinburgh Medical School, Edinburgh, United Kingdom; 71 MRC Human Genetics Unit, Institute of Genetics and Molecular Medicine, Western General Hospital, Edinburgh, United Kingdom; 72 Institute of Clinical Molecular Biology, Christian-Albrechts University, Kiel, Germany; 73 popgen Biobank, University Hospital Schleswig-Holstein, Kiel, Germany; 74 Clinic for Prosthodontic Dentistry, Gerostomatology, and Material Science, University of Greifswald, Greifswald, Germany; 75 Institute of Anatomy and Cell Biology, University of Greifswald, Greifswald, Germany; 76 Institute of Pharmacology, University of Greifswald, Greifswald, Germany; 77 Institute of Clinical Chemistry and Laboratory Medicine, Ernst-Moritz-Arndt-University Greifswald, Greifswald, Germany; 78 Clinic for Internal Medicine A, University of Greifswald, Greifswald, Germany; 79 Institute for Community Medicine, University of Greifswald, Greifswald, Germany; 80 Department of Medicine, University of Leipzig, Leipzig, Germany; 81 Wellcome Trust Centre for Human Genetics and Oxford Centre for Diabetes, Endocrinology, and Metabolism, University of Oxford, Oxford, United Kingdom; 82 Department of Internal Medicine, Erasmus University Medical Center, Rotterdam, The Netherlands; 83 Croatian Centre for Global Health, Faculty of Medicine, University of Split, Split, Croatia; 84 MRC Human Genetics Unit, Institute of Genetics and Molecular Medicine, Western General Hospital, Edinburgh, United Kingdom; 85 INSERM U897, Université Victor Ségalen Bordeaux 2, ISPED, Bordeaux, France; 86 Université Bordeaux 2 Victor Segalen, Bordeaux, France; 87 Centre for Vision Research, Westmead Millennium Institute, Westmead Hospital, University of Sydney, Sydney, Australia; 88 Centre for Eye Research Australia (CERA), University of Melbourne, Melbourne, Australia; 89 Department of Clinical Physiology, University of Tampere and Tampere University Hospital, Tampere, Finland; 90 Department of Medicine, University of Turku and Turku University Hospital, Turku, Finland; 91 NHS Tayside, Wellcome Trust Centre for Molecular Medicine, Clinical Research Centre, Ninewells Hospital, University of Dundee, Dundee, United Kingdom; 92 Institute for Maternal and Child Health, IRCCS “Burlo Garofolo,” University of Trieste, Trieste, Italy; 93 University Medical Centre Mannheim, 5th Department of Medicine, Mannheim, Germany; 94 Robertson Centre for Biostatistics, University of Glasgow, Glasgow, United Kingdom; 95 Department of Pharmacology and Therapeutics, University College Cork, Cork, Ireland; 96 First Department of Internal Medicine, Paracelsus Medical University, Salzburg, Austria; 97 Division of Genetic Epidemiology, Innsbruck Medical University, Innsbruck, Austria; 98 Inserm UMRS 1018, CESP Team 10, Université Paris Sud, Villejuif, France; 99 Department of Internal Medicine, Centre Hospitalier Universitaire Vaudois, Lausanne, Switzerland; 100 Research Centre of Applied and Preventive Cardiovascular Medicine, Department of Clinical Physiology, Turku University Hospital, University of Turku, Turku, Finland; 101 Biomedical Research Institute, Ninewells Hospital and Medical School, University of Dundee, Dundee, United Kingdom; 102 Interuniversity Cardiology Institute of the Netherlands (ICIN), Utrecht, The Netherlands; 103 Einthoven Laboratory for Experimental Vascular Medicine, Leiden, The Netherlands; 104 Durrer Center for Cardiogenetic Research, Amsterdam, The Netherlands; 105 Geriatric Research and Education Clinical Center, Veterans Administration Medical Center, Baltimore, Maryland, United States of America; 106 Welch Center for Prevention, Epidemiology, and Clinical Research, Baltimore, Maryland, United States of America; 107 Department of Epidemiology, School of Public Health, University of Michigan, Ann Arbor, Michigan, United States of America; 108 Brigham and Women's Hospital and Channing Laboratory, Harvard Medical School, Boston, Massachusetts, United States of America; 109 Institute of Physiology, University of Greifswald, Greifswald, Germany; 110 Harvard Medical School, Boston, Massachusetts, United States of America; 111 Division of Nephrology, University of Maryland Medical School, Baltimore, Maryland, United States of America; 112 University Institute of Social and Preventive Medicine, Centre Hospitalier Universitaire Vaudois and University of Lausanne, Epalinges, Switzerland; 113 Department of Epidemiology and Preventive Medicine, University Hospital Regensburg, Regensburg, Germany; 114 Institute of Epidemiology I, Helmholtz Zentrum München, German Research Center for Environmental Health, Neuherberg, Germany; 115 Divisions of Genetics and Gastroenterology, Department of Internal Medicine, Brigham and Women's Hospital, Boston, Massachusetts, United States of America; 116 Harvard Stem Cell Institute, Harvard University, Cambridge, Massachusetts, United States of America; 117 Division of Endocrinology, Brigham and Women's Hospital and Harvard Medical School, Boston, Massachusetts, United States of America; Georgia Institute of Technology, United States of America

## Abstract

Chronic kidney disease (CKD) is an important public health problem with a genetic component. We performed genome-wide association studies in up to 130,600 European ancestry participants overall, and stratified for key CKD risk factors. We uncovered 6 new loci in association with estimated glomerular filtration rate (eGFR), the primary clinical measure of CKD, in or near *MPPED2*, *DDX1*, *SLC47A1*, *CDK12*, *CASP9*, and *INO80*. Morpholino knockdown of *mpped2* and *casp9* in zebrafish embryos revealed podocyte and tubular abnormalities with altered dextran clearance, suggesting a role for these genes in renal function. By providing new insights into genes that regulate renal function, these results could further our understanding of the pathogenesis of CKD.

## Author Summary

Chronic kidney disease (CKD) is an important public health problem with a hereditary component. We performed a new genome-wide association study in up to 130,600 European ancestry individuals to identify genes that may influence kidney function, specifically genes that may influence kidney function differently depending on sex, age, hypertension, and diabetes status of individuals. We uncovered 6 new loci associated with estimated glomerular filtration rate (eGFR), the primary measure of renal function, in or near *MPPED2*, *DDX1*, *SLC47A1*, *CDK12*, *CASP9*, and *INO80*. *CDK12* effect was stronger in younger and absent in older individuals. *MPPED2*, *DDX1*, *SLC47A1*, and *CDK12* loci were associated with eGFR in African ancestry samples as well, highlighting the cross-ethnicity validity of our findings. Using the zebrafish model, we performed morpholino knockdown of *mpped2* and *casp9* in zebrafish embryos and revealed podocyte and tubular abnormalities with altered dextran clearance, suggesting a role for these genes in renal function. These results further our understanding of the pathogenesis of CKD and provide insights into potential novel mechanisms of disease.

## Introduction

Chronic kidney disease (CKD) affects nearly 10% of the global population [1], [2], and its prevalence continues to increase [3]. Reduced estimated glomerular filtration rate (eGFR), the primary measure used to define CKD (eGFR<60 ml/min/1.73 m^2^) [4], is associated with an increased risk of cardiovascular morbidity and mortality [5], acute kidney injury [6], and end stage renal disease (ESRD) [6], [7].

Using genome-wide association studies (GWAS) in predominantly population-based cohorts, we and others have previously identified more than 20 genetic loci associated with eGFR and CKD [8]–[11]. Although most of these genetic effects seem largely robust across strata of diabetes or hypertension status [9], evidence suggests that some of the loci such as the *UMOD* locus may have heterogeneous effects across these strata [11]. We thus hypothesized that GWAS in study populations stratified by four key CKD risk factors - age, sex, diabetes or hypertension status - may permit the identification of novel eGFR and CKD loci. We carried this out by extending our previous work [9] to a larger discovery sample of 74,354 individuals with independent replication in additional 56,246 individuals, resulting in a total of 130,600 individuals of European ancestry. To assess for potential heterogeneity, we performed separate genome-wide association analyses across strata of CKD risk factors, as well as in a more extreme CKD phenotype.

## Results

Meta-analyses of GWAS on the 22 autosomes were performed for: 1) eGFR based on serum creatinine (eGFRcrea) and CKD (6,271 cases) in the overall sample, 2) eGFRcrea and CKD stratified by the four risk factors, and 3) CKD45, a more severe CKD phenotype defined as eGFRcrea <45 ml/min/1.73 m^2^ in the overall sample (2,181 cases). For the stratified analyses, in addition to identifying loci that were significant within each stratum, we performed a genome-wide comparison of the effect estimates between strata of the four risk factors. A complete overview of the analysis workflow is given in Figure S1. All studies participating in the stage 1 discovery and stage 2 replication phases are listed in Tables S1 and S2. The characteristics of all stage 1 discovery samples by study are reported in Table S3, and information on study design and genotyping are reported in Table S4. Results of the eGFRcrea analyses are summarized in the Manhattan and quantile-quantile plots reported in Figures S2 and S3. A total of 21 SNPs from the discovery stage were carried forward for replication in an independent set of 56,246 individuals (Tables S5 and S6). These SNPs were selected for replication for the following (Figure S1): 5 reached genome-wide significance in either eGFRcrea overall or stratified analyses, 1 based on a test of direction-consistency of SNP-eGFR associations across the discovery cohorts for eGFRcrea overall, 4 demonstrated a *P* value≤10^−6^ and high between-study homogeneity (I^2^<25%) in the CKD45 analysis (Table S7), and 11 demonstrated between-strata *P* value≤5×10^−5^ along with a *P* value≤5×10^−5^ for association with eGFRcrea in at least one of the two strata (Table S8).

While none of the loci identified for CKD45 or the test for between-strata difference analyses replicated, all 6 loci identified from the eGFRcrea overall analysis, stratified analyses, and the direction test did (Table 1). These 6 loci were identified and replicated in the overall analysis (rs3925584, located upstream of the *MPPED2* gene; rs6431731 near the *DDX1* gene), in the diabetes-free sub-group (rs2453580 in an intron of the *SLC47A1* gene), in the younger age stratum (rs11078903 in an intron of the *CDK12* gene; rs12124078 located near the *CASP9* gene), and the direction test (rs2928148, located in the *INO80* gene, see Methods for details). In the combined meta-analysis of all 45 studies used in the discovery and replication stages, all six SNPs met the genome-wide significance threshold of 5×10^−8^, with individual *P* values ranging from 4.3×10^−8^ to 8.4×10^−18^ (Table 1). The imputation quality of these SNPs is reported in Table S9, and Figure S4 shows the regional association plots for each of the 6 loci. We also confirmed all previously identified renal function loci in the current data (Table S10). Brief descriptions of the genes included within the 6 new loci uncovered can be found in Table S11. Forest plots for the associations between the index SNP at each of the 6 novel loci and eGFR across all discovery studies and all strata are presented in Figures S5 and S6. Most of the 6 new loci had similar associations across strata of CKD risk factors except for the *CDK12* locus, which revealed stronger association in the younger (≤65 years of age) as compared to the older age group (>65 years of age).

**Table 1:** Novel loci associated with eGFRcrea.

Locus description						Discovery analysis		Replication analysis			Combined analysis†		
Analysis subgroup	SNP ID	Chr	Position (bp)‡	Genes nearby‡	Ref./Non-Ref. alleles (RAF)	Effect(SE)§	*P* value§	Effect(SE)	1-sided *P* value	Q value	Effect(SE)	*P* value	I^2^
Overall	rs3925584	11	30,716,911	*MPPED2*	T/C(0.54)	−0.0077(0.0013)	1.0×10^−09^	−0.0073(0.0013)	4.0×10^−9^	1.1×10^−08^	−0.0075(0.0009)	8.4×10^−18^	21%
Overall	rs6431731	2	15,780,453	*DDX1*	T/C(0.94)	−0.0181(0.0033)	4.6×10^−08^	−0.0065(0.0034)	0.0277	0.0195	−0.0127(0.0023)	4.3×10^−08^	11%
No Diabetes	rs2453580	17	19,378,913	***SLC47A1***	T/C(0.59)	0.0076(0.0014)	4.6×10^−08^	0.0038(0.0014)	0.0037	0.0039	0.0059(0.0010)	2.1×10^−09^	21%
Age≤65 yrs*	rs12124078	1	15,742,486	***DNAJC16***, *CASP9*, *AGMAT*	A/G(0.70)	0.0096(0.0015)	9.8×10^−10^	0.0098(0.0017)	5.0×10^−9^	1.1×10^−08^	0.0097(0.0011)	1.5×10^−17^	20%
Age≤65 yrs	rs11078903	17	34,885,450	***CDK12***, *MED1*, *FBXL20*	A/G(0.76)	−0.0103(0.0017)	2.4×10^−09^	−0.0083(0.0023)	1.4×10^−4^	2.0×10^−04^	−0.0096(0.0013)	9.0×10^−13^	0%
Direction Test (Overall)**	rs2928148	15	39,188,842	***INO80***, *EXD1*, *CHAC1*	A/G(0.52)	0.0064(0.0012)	1.2×10^−07^	0.0033(0.0015)	0.0145	0.0122	0.0051(0.0009)	4.0×10^−08^	0%

SNPs are listed in the stratum where the smallest *P* value in the discovery analysis was observed. Sample size/number of studies in the discovery phase: 74,354/26 (overall, direction test), 66,931/24 (No Diabetes), 46,435/23 (age ≤65 years); replication phase: 56,246/19 (overall, direction test), 41,218/17 (No Diabetes), 28,631/16 (age ≤65 years); combined analysis: 130,600/45 (overall, direction test), 108,149/41 (No Diabetes), 75,066/39 (age ≤65 years).

Chr.: chromosome; bp: base-pairs; Ref./Non-Ref. All.: reference/non-reference alleles; RAF: reference allele frequency; SE: standard error.

‡ Genes nearby were based on RefSeq genes (build 36). The gene closest to the SNP is listed first and is in boldface if the SNP is located within the gene.

§ Effects on log(eGFRcrea); post GWAS meta-analysis genomic control correction applied to *P* values and SEs.

* While being uncovered in the younger samples, this locus showed consistent results in the non-diabetic group (combined-analysis *P* value 5.7×10^−16^) and in the overall population (*P* value 9.5×10^−22^) - see Tables S16 and S10 for additional details.

** The direction test was performed in the overall dataset; the genomic control corrected *P* value from the direction test for the SNP rs2928148 was 4.0×10^−7^. In the combined analysis, the largest effect size (0.0054 on log eGFR in ml/min/1.73 m^2^) and the smallest *P* value (3.7×10^−8^) were observed in the non-diabetic group.

† All results were confirmed by random-effect meta-analysis.

**Figure 1:**
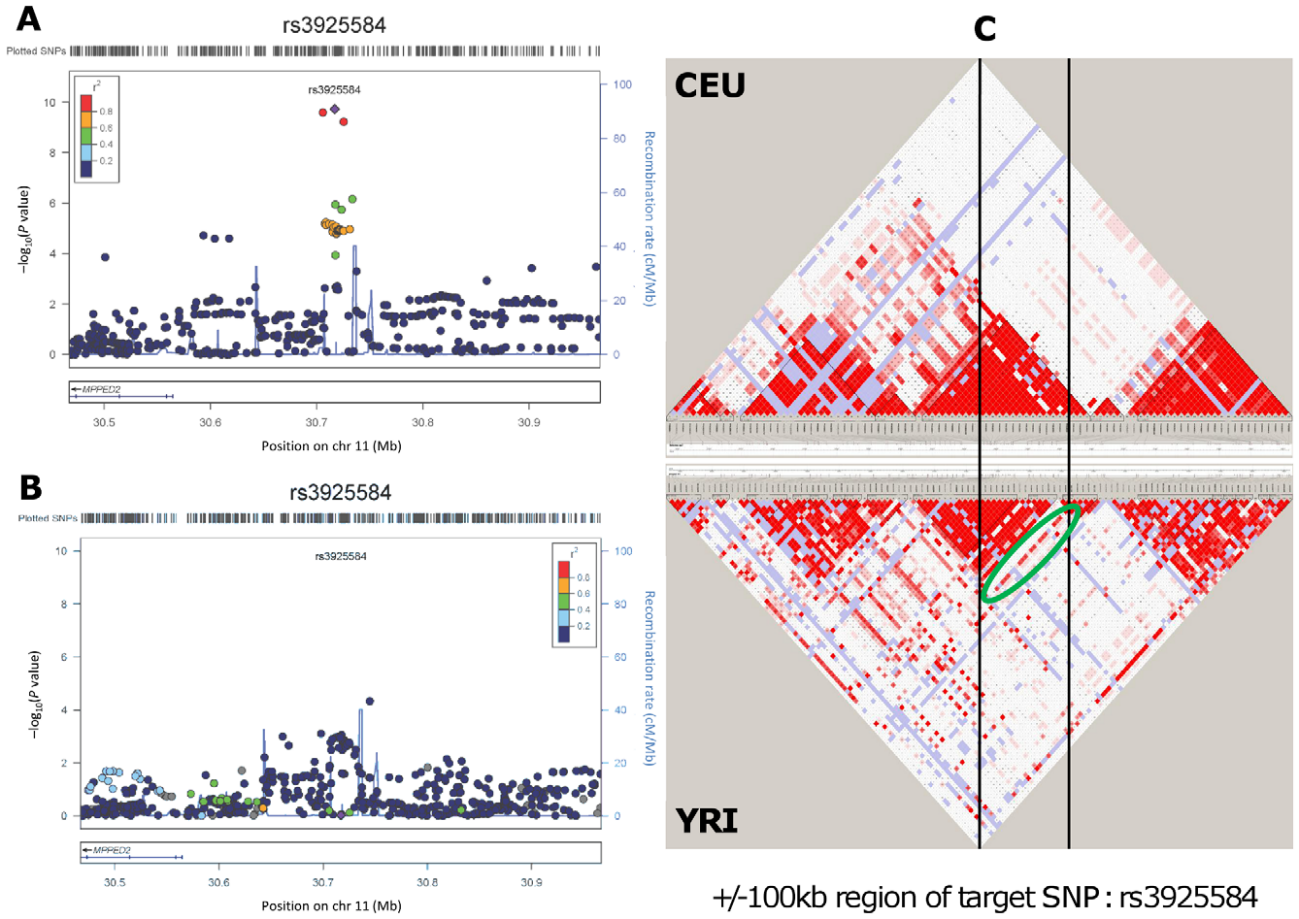
Genetic association and LD distribution of the *MPPED2* gene locus in European and African ancestry populations. Regional association plots in the CKDGen European ancestry discovery analysis (N = 74,354) (A) and in the CARe African ancestry discovery analysis (N = 8,110) (B). LD structure: comparison between the HapMap release II – CEU and YRI samples in the region included within +/−100 kb from the target SNP rs3925584 identified in the CKDGen GWAS. The green circle highlights a stream of high LD connecting the two blocks, indicating the presence of common haplotypes (C).

We further examined our findings in 8,110 African ancestry participants from the CARe consortium [12] (Table 2). Not surprisingly, given linkage disequilibrium (LD) differences between Europeans and African Americans, none of the 6 lead SNPs uncovered in CKDGen achieved significance in the African American samples. Next, we interrogated the 250 kb flanking regions from the lead SNP at each locus, and showed that 4 of the 6 regions (*MPPED2*, *DDX1*, *SLC47A1*, and *CDK12*) harbored SNPs that achieved statistical significance after correcting for multiple comparisons based on the genetic structure of each region (see Methods for details). Figure 1 presents the regional association plots for *MPPED2*, and Figure S7 presents the plots of the remaining loci in the African American sample. Imputation scores for the lead SNPs can be found in Table S12. We observed that rs12278026, upstream of *MPPED2*, was associated with eGFRcrea in African Americans (*P* value = 5×10^−5^, threshold for statistical significance: *P* value = 0.001). While rs12278026 is monomorphic in the CEU population in HapMap, rs3925584 and rs12278026 have a D′ of 1 (r^2^ = 0.005) in the YRI population, suggesting that these SNPs may have arisen from the same ancestral haplotype.

**Table 2:** Interrogation of the six novel loci uncovered in the European ancestry (EA) individuals (CKDGen consortium) in individuals of African ancestry (AA) from the CARe consortium for the trait eGFRcrea.

Results for the lead SNPs in the CARe AA individuals					Best SNP in region in the CARe AA individuals							
SNP ID*	Nearby genes§	Ref./Non-Ref. alleles (RAF)	Effect(SE)	*P* value	SNP ID	Position (build 36)	LD (R^2^) with lead SNP	RAF (Ref./Non-Ref. alleles)	Effect(SE)	*P* value	S**	Bonferroni *P* value threshold (0.05/S)
rs3925584	*MPPED2*	T/C (0.88)	−0.0005(0.0066)	0.9349	rs12278026	30,744,460	0.005	0.89 (A/G)	0.0342(0.0084)	4.6×10^−5^	46	0.0011
rs6431731	*DDX1*	T/C (0.99)	−0.0181(0.0213)	0.3948	rs4669002	15,874,859	NA†	0.56 (T/C)	−0.0196(0.0047)	2.6×10^−5^	78	6.4×10^−4^
rs12124078	***SLC47A1***	A/G (0.69)	−0.0024(0.0045)	0.5956	rs1472554	15,987,920	0.004	0.50 (C/G)	−0.0120(0.0041)	0.0035	44	0.0011
rs2453580	***DNAJC16***, *CASP9*, *AGMAT*	T/C (0.59)	0.0056(0.0049)	0.2524	rs1800869	19,505,226	0.011	0.93 (C/G)	−0.0294(0.0082)	3.6×10^−4^	33	0.0015
rs11078903‡	***CDK12***, *MED1*, *FBXL20*	A/G (NA‡)	NA‡	NA‡	rs1874226	34,982,557	0.112	0.34 (T/C)	0.0157(0.0045)	4.2×10^−4^	15	0.0033
rs2928148	***INO80***, *EXD1*, *CHAC1*	A/G (0.22)	−0.0003(0.0053)	0.9497	rs8039934	39,284,719	0.105	0.50 (T/C)	−0.0086(0.0042)	0.0412	22	0.0023

Ref./Non-Ref. All.: reference/non-reference alleles; RAF: reference allele frequency; SE: standard error.

* Characteristics of the six lead SNPs in the EA individuals from the CKDGen consortium can be found in Table 1.

§ The gene closest to the SNP is listed first and is in boldface if the SNP is located within the gene.

** S = number of independent, typed SNPs interrogated.

† No LD information available in the HapMap database between the target SNP and the best SNP in the DDX1 region.

‡ The SNP rs11078903 was not present in the CARe consortium database.

We also performed eQTL analyses of our 6 newly identified loci using known databases and a newly created renal eSNP database (see Methods) and found that rs12124078 was associated with *cis* expression of the nearby *CASP9* gene in myocytes, which encodes caspase-9, the third apoptotic activation factor involved in the activation of cell apoptosis, necrosis and inflammation (*P* value for the monocyte eSNP of interest = 3.7×10^−13^). In the kidney, caspase-9 may play an important role in the medulla response to hyperosmotic stress [13] and in cadmium-induced toxicity [14]. The other 5 SNPs were not associated with any investigated eQTL. Additional eQTL analyses of 81 kidney biopsies (Table S13) did not reveal further evidence of association with eQTLs (Table S14).

**Figure 2:**
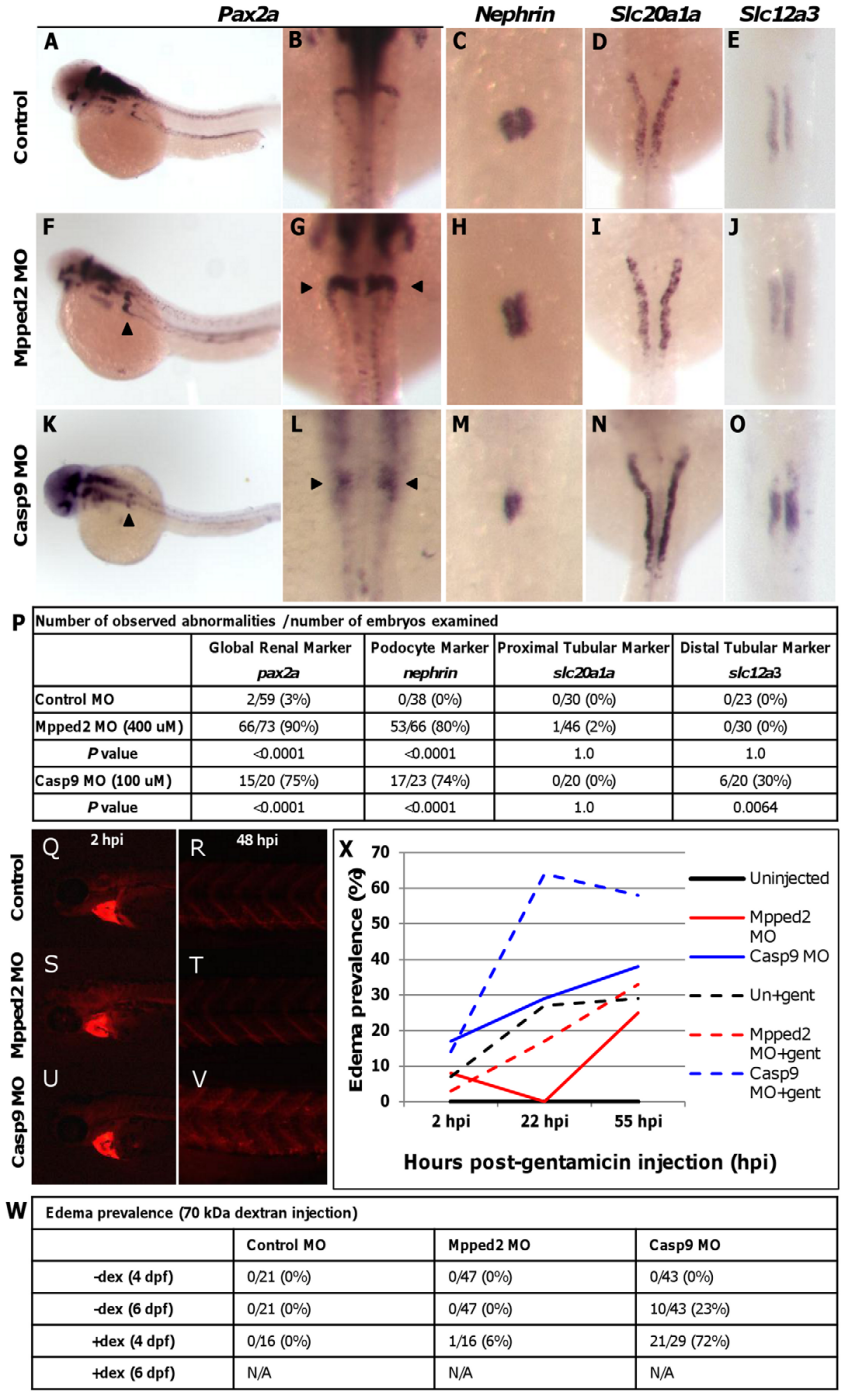
*Mpped2* and *casp9* knockdowns result in defective kidney development. (A–E) Whole mount *in situ* hybridization in control embryos demonstrates normal expression of the global kidney marker *pax2a* (A: lateral view; B: dorsal view), the glomerular marker *nephrin* (C), and the tubular markers *slc20a1a* (proximal tubule, D), and *slc12a3* (distal tubule, E) at 48 hours post fertilization (hpf). (F–J) *Mpped2* morpholino (MO) knockdown embryos develop glomerular gene expression defects (F–H, arrowheads), but tubular marker expression is normal (I, J). (K–O) *Casp9* MO knockdown embryos demonstrate reduced glomerular gene expression (K–M, arrowheads) and shortened distal tubules (O). (P) Quantification of observed abnormalities per number of embryos reveal significant differences in expression of *pax2a* and *nephrin* in response to knockdown of both *mpped2* and *casp9* (Fisher's exact test). (Q–V) Embryos were injected with control, *mpped2*, *or casp9* MO at the one-cell stage and subsequently injected with 70,000 MW fluorescent rhodamine dextran at 80 hpf. Dextran fluorescence was monitored over the next 48 hours. All dextran-injected embryos show equal loading into the cardiac sinus venosus at 2 hours post-injection (2 hpi/82 hpf; Q, S, U). Compared to control MO-injected embryos (R) and *mpped2* knockdown embryos (T), knockdown of *casp9* resulted in reduced dextran clearance at 48 hpi as shown by increased trunk fluorescence (V). (W) *Casp9* knockdown results in increased susceptibility to edema formation both spontaneously (−dex) (*P* value = 0.0234, Fisher's exact test) and after dextran challenge (+dex) (*P* value<0.0001). Embryos injected with both MO and dextran did not survive to 6 dpf (N/A). (X) Edema develops earlier and with higher frequency in *casp9* morphants following injection of the nephrotoxin gentamicin.

Of the 6 novel loci identified, 2 (*MPPED2* and *DDX1*) were in regions containing only a single gene, and 1 (*CASP9*) had its expression associated with the locus lead SNP. Thus, to determine the potential involvement of these three genes during zebrafish kidney development, we independently assessed the expression of 4 well-characterized renal markers following morpholino knockdown: *pax2a* (global kidney) [15], *nephrin* (podocyte) [16], *slc20a1a* (proximal tubule) [17], and *slc12a3* (distal tubule) [17]. While we observed no abnormalities in *ddx1* morphants (Figure S8), *mpped2* and *casp9* knockdown resulted in expanded *pax2a* expression in the glomerular region in 90% and 75% of morphant embryos, respectively, compared to 0% in controls (*P* value<0.0001 for both genes; Figure 2A versus 2F and 2K; 2B versus 2G and 2L; and 2P). Significant differences were also observed in expression of the podocyte marker *nephrin* (Figure 2C versus 2H and 2M; 80% and 74% abnormalities for *mpped2* and *casp9*, respectively, versus 0% in controls, *P* value<0.0001 for both genes). For *mpped2*, no differences were observed in expression of the proximal or distal tubular markers *slc20a1a* and *slc12a3* (*P* value = 1.0; Figure 2D versus 2I and 2E versus 2J). *Casp9* morphants and controls showed no differences in proximal tubular marker expression (Figure 2D versus 2N), but abnormalities were observed in distal tubular marker expression in *casp9* knockdown embryos (30% versus 0%; Figure 2E versus 2O; *P* value = 0.0064).

*Casp9* morphants displayed diminished clearance of 70,000 MW fluorescent dextran 48 hours after injection into the sinus venosus compared to controls, revealing significant functional consequences of *casp9* knockdown (Figure 2Q–2V). No clearance abnormalities were observed in *mpped2* morphants. The occurrence of abdominal edema is a non-specific finding that is frequently observed in zebrafish embryos with kidney defects. We examined the occurrence of edema in *mpped2* and *casp9* knockdown embryos at 4 and 6 days post fertilization (dpf), both in the absence and presence of dextran, and observed a significant increase in edema prevalence in *casp9* with (*P* value<0.0001) and without (*P* value = 0.0234) dextran challenge but not in *mpped2* morphants (Figure 2W).

In order to further demonstrate differences in kidney function in response to knockdown of *mpped2* and *casp9*, we injected the nephrotoxin gentamicin which predictably causes edema in a subset of embryos. Casp9 morphants were more susceptible to developing edema compared to both controls and mpped2 morphants (Figure 2X). In addition, edema developed earlier and was more severe, encompassing a greater area of the entire embryo (Figure S9). Together, these findings suggest that *casp9* and *mpped2* knockdowns result in altered kidney gene expression and function. Specifically, abnormal expression of *pax2a* and *nephrin* in casp9 morphants in addition to dextran retention and edema formation suggest loss of *casp9* impacts glomerular development and function.

The lead SNP at the *MPPED2* locus is located approximately 100 kb upstream of the gene metallophosphoesterase domain containing 2 (*MPPED2*), which is highly evolutionary conserved and encodes a protein with metallophosphoesterase activity [18]. It has been recognized for a role in brain development and tumorigenesis [19] but thus far not for kidney function.

To determine whether the association at our newly identified eGFRcrea loci was primarily due to creatinine metabolism or renal function, we compared the relative associations between eGFRcrea and eGFR estimated using cystatin C (eGFRcys) (Figure S10, File S1). The new loci showed similar effect sizes and consistent effect directions for eGFRcrea and eGFRcys, suggesting a relation to renal function rather than to creatinine metabolism. Placing the results of these 6 loci in context with our previously identified loci [8], [9] (23 known and 6 novel), 18 were associated with CKD at a 0.05 significance level (odds ratio, OR, from 1.05 to 1.26; *P* values from 3.7×10^−16^ to 0.01) and 11 with CKD45 (OR from 1.08 to 1.34; *P* values from 1.1×10^−5^ to 0.047; Figure S11 and Table S15).

When we examined these 29 renal function loci by age group, sex, diabetes and hypertension status (Tables S16, S17, S18, and S19), we observed consistent associations with eGFRcrea for most loci across all strata, with only two exceptions: *UMOD* had a stronger association in older individuals (*P* value for difference 8.4×10^−13^) and in those with hypertension (*P* value for difference 0.002), and *CDK12* was stronger in younger subjects (*P* value for difference 0.0008). We tested the interaction between age and rs11078903 in one of our largest studies, the ARIC study. The interaction was significant (*P* value = 0.0047) and direction consistent with the observed between-strata difference.

Finally, we tested for associations between our 6 new loci and CKD related traits. The new loci were not associated with urinary albumin-to-creatinine ratio (UACR) or microalbuminuria [20] (Tables S20 and S21), with blood pressure from the ICBP Consortium [21] (Table S22) or with myocardial infarction from the CARDIoGRAM Consortium [22] (Table S23).

## Discussion

We have extended prior knowledge of common genetic variants for kidney function [8]–[11], [23] by performing genome-wide association tests within strata of key CKD risk factors, including age, sex, diabetes, and hypertension, thus uncovering 6 loci not previously known to be associated with renal function in population-based studies (*MPPED2*, *DDX1*, *CASP9*, *SLC47A1*, *CDK12*, *INO80*). In contrast to our prior genome-wide analysis [8], [9], the majority of the new loci uncovered in the present analysis have little known prior associations with renal function. This highlights a continued benefit of the GWAS approach by using large sample sizes to infer new biology.

Despite our hypothesis that genetic effects are modified by CKD risk factors, most of the identified variants did not exhibit strong cross-strata differences. This highlights that many genetic associations with kidney function may be shared across risk factor strata. The association of several of these loci with kidney function in African Americans underscores the generalizability of identified renal loci across ethnicities. Zebrafish knockdown of *mpped2* resulted in abnormal podocyte anatomy as assessed by expression of glomerular markers, and loss of *casp9* led to altered podocyte and distal tubular marker expression, decreased dextran clearance, edema, and enhanced susceptibility to gentamicin-induced kidney damage. These findings demonstrate the potential importance of these genes with respect to renal function and illustrate that zebrafish are a useful *in vivo* model to explore the functional consequences of GWAS-identified genes.

Despite these strengths, there are some limitations of our study that warrant discussion. Although we used cystatin C to separate creatinine metabolism from true filtration loci, SNPs within the cystatin C gene cluster have been shown to be associated with cystatin C levels [8], which might result in some degree of misclassification in absolute levels. While we used standard definitions of diabetes and hypertension in the setting of population-based studies, these may differ from those definitions used in clinical practice. In addition, we were unable to differentiate the use of anti-hypertension medications from other clinical indications of these agents or type 1 from type 2 diabetes. The absence of association between our six newly discovered SNPs and the urinary albumin to creatinine ratio, blood pressure, and cardiovascular disease may have resulted from disparate genetic underpinnings of these traits, the overall small effect sizes, or the cross-sectional nature of our explorations; and we were unable to differentiate between these potential issues. Finally, power was modest to detect between-strata heterogeneity.

With increased sample size and stratified analyses, we have identified additional loci for kidney function that continue to have novel biological implications. Our primary findings suggest that there is substantial generalizability of SNPs associations across strata of important CKD risk factors, specifically with hypertension and diabetes.

## Materials and Methods

### Phenotype definition

Serum creatinine and cystatin C were measured as detailed in Tables S1 and S2. To account for between-laboratory variation, serum creatinine was calibrated to the US nationally representative National Health and Nutrition Examination Study (NHANES) standards in all discovery and replication studies as described previously [8], [24], [25]. GFR based on serum creatinine (eGFRcrea) was estimated using the four-variable MDRD Study equation [26]. GFR based on cystatin C (eGFRcys) was estimated as eGFRcys = 76.7×(serum cystatin C)^−1.19^ [27]. eGFRcrea and eGFRcys values<15 ml/min/1.73 m^2^ were set to 15, and those >200 were set to 200 ml/min/1.73 m^2^. CKD was defined as eGFRcrea <60 ml/min/1.73 m^2^ according to the National Kidney Foundation guidelines [28]. A more severe CKD phenotype, CKD45, was defined as eGFRcrea <45 ml/min/1.73 m^2^. Control individuals for both CKD and CKD45 analyses were defined as those with eGFRcrea >60 ml/min/1.73 m^2^.

### Covariate definitions

In discovery and replication cohorts, diabetes was defined as fasting glucose ≥126 mg/dl, pharmacologic treatment for diabetes, or by self-report. Hypertension was defined as systolic blood pressure ≥140 mmHg or diastolic blood pressure ≥90 mmHg or pharmacologic treatment for hypertension.

### Discovery analyses

Genotyping was conducted as specified in Table S4. After applying quality-control filters to exclude low-quality SNPs or samples, each study imputed up to ∼2.5 million HapMap-II SNPs, based on the CEU reference samples. Imputed genotypes were coded as the estimated number of copies of a specified allele (allelic dosage). Additional, study-specific details can be found in Table S1.

### Primary association analysis

A schematic view of our complete analysis workflow is presented in Figure S1. Using data from 26 population-based studies of individuals of European ancestry, we performed GWA analyses of the following phenotypes: 1) log_e_(eGFRcrea), log_e_(eGFRcys), CKD, and CKD45 overall and 2) log_e_(eGFRcrea) and CKD stratified by diabetes status, hypertension status, age group (≤/>65 years), and sex. GWAS of log_e_(eGFRcrea) and log_e_(eGFRcys) were based on linear regression. GWAS of CKD and CKD45 were performed in studies with at least 25 cases (i.e. all 26 studies for CKD and 11 studies for CKD45) and were based on logistic regression. Additive genetic effects were assumed and models were adjusted for age and, where applicable, for sex, study site and principal components. Imputation uncertainty was accounted for by including allelic dosages in the model. Where necessary, relatedness was modeled with appropriate methods (see Table S1 for study-specific details). Before including in the meta-analysis, all GWA data files underwent to a careful quality control, performed using the GWAtoolbox package in R (www.eurac.edu/GWAtoolbox.html) [29].

Meta-analyses of study-specific SNP-association results, assuming fixed effects and using inverse-variance weighting, i.e.: the pooled effect 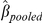 is estimated as 
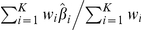
, where 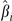 is the effect of the SNP on the outcome in the *i* ^th^ study, *K* is the number of studies, and 
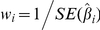
 is the weight given to the *i* ^th^ study. The meta-analyses were performed using METAL [30], with genomic control correction applied across all imputed SNPs [31] if the inflation factor λ>1 at both the individual study level and after the meta-analysis. SNPs with minor allele frequency (MAF)<1% were excluded. All SNPs with a meta-analysis *P* value≤5×10^−8^ for any trait or any stratum were deemed genome-wide significant [32].

In the eGFRcrea analyses, after excluding loci that were previously reported [8], [9], we selected for replication all SNPs with *P* value<5×10^−8^ in any trait or stratum that were independent (defined by pairwise *r* ^2^<0.2), in the primary association analysis. This yielded five SNPs in five independent loci. The same criterion was applied to the CKD analysis, where no SNPs passed the selection threshold. Given the smaller number of cases with severe CKD resulting in less statistical power, a different selection strategy was adopted for the CKD45 analysis: selected for replication were SNPs with discovery *P* value≤5×10^−6^, MAF≥5%, and homogeneous effect size across studies (I^2^≤25%). Four additional SNPs were thereby selected for replication from the CKD45 analysis.

### Direction test to identify SNPs for replication

In addition to identifying SNPs for replication based on the genome-wide significance threshold from a fixed effect model meta-analysis, we performed a “direction test” to identify additional SNPs for which between-study heterogeneity in effect size might have obscured the overall association that was nevertheless highly consistent in the direction of allelic effects. Under the null hypothesis of no association, the *a priori* probability that a given effect allele of a SNP has either a positive or negative association with eGFRcrea is 0.5. Because the meta-analysis includes independent studies, the number of concordant effect directions follows a binomial distribution. Therefore, we tested whether the number of discovery cohorts with the same sign of association (i.e. direction of effect) was greater than expected by chance given the binomial distribution and a null expectation of equal numbers of associations with positive and negative sign. The test was only applied for eGFRcrea in the overall analysis. Multiple testing was controlled by applying the same *P* value threshold of 5×10^−8^ as in the overall GWAS. Given that no SNP met this criterion, we selected for replication one novel SNP with the lowest *P* value of 4.0×10^−7^.

### Genome-wide between-strata difference test to identify SNPs for replication

Based on the results of the stratified GWAS of eGFRcrea and CKD, for each SNP we tested the hypothesis whether the effect of a SNP on eGFRcrea or CKD was the same between strata (null hypothesis), i.e. diabetes versus non-diabetes subjects, hypertensive versus normotensive, younger versus older, females versus males. We used a two-sample test defined as Z = (b_1_−b_2_)/(SE(b_1_)^2^+SE(b_2_)^2^)^0.5^, with b_1_ and b_2_ indicating the effect estimates in the two strata and SE(b_1_) and SE(b_2_) their standard errors [33]. For large samples, the test statistic follows a standard normal distribution. SNPs were selected for replication if they had a between-stratum difference *P* value≤5×10^−5^, an association *P* value≤5×10^−5^ in one of the two strata, and MAF≥10%. Independent loci were defined using the same criteria as described above. Eleven further SNPs, one per locus, were selected for replication from the between-strata difference test.

### Replication analysis

Replication was performed for a total of 21 SNPs including 5 from the overall and stratified eGFRcrea analyses, 1 from the direction test on eGFRcrea, 4 from the overall CKD45 analysis, and 11 from the between-strata difference test. Replication studies used the same phenotype definition, and had available genotypes from imputed *in silico* genome-wide SNP data or *de novo* genotyping. The same association analyses including the identical stratifications were performed as in discovery studies. Details can be found in the Tables S2, S5 and S6. Study-specific replication results for the selected SNPs were combined using the same meta-analysis approach and software as in the discovery stage. One-sided *P* values were derived with regard to the effect direction found in the discovery stage. Based on the *P* value distribution of all SNPs submitted for replication (the 10 from eGFRcrea and CKD45 and the 11 from the between strata difference test), we estimated the False Discovery Rate as a q-value using the QVALUE [34] package in R. SNPs with q-value<0.05 were called significantly replicating, thus specifying a list of associations expected to include not more than 5% false positives.

Finally, study-specific results from both the discovery and replication stage were combined in a joint inverse-variance weighted fixed-effect meta-analysis and the two-sided *P* values were compared to the genome-wide significance threshold of 5×10^−8^ to test whether a SNP was genome-wide significant. Between-study heterogeneity of replicated SNPs was quantified by the I^2^ statistic [35].

### Replication genotyping

For *de novo* genotyping in 10,446 samples from KORA F3, KORA F4, SAPHIR and SAPALDIA, the MassARRAY system at the Helmholtz Zentrum (München, Germany) was used, using Assay Design v3.1.2 and the iPLEX chemistry (Sequenom, San Diego, USA). Assay design failed for rs1322199 and genotyping was not performed. Ten percent of the spectra were checked by two independent, trained persons, and 100% concordance between investigators was obtained. SNPs with a *P* value<0.001 when testing for Hardy-Weinberg equilibrium (rs10490130, rs10068737, rs11078903), SNPs with call rate <90% (rs500456 in KORA F4 only) or monomorphic SNPs (rs2928148) were excluded from analyses without attempting further genotyping. The call rates of rs4149333 and rs752805 were near 0% on the MassARRAY system. These SNPs were thus genotyped on a 7900HT Fast Real-Time PCR System (Applied Biosystems, Foster City, USA). Mean call rate across all studies and SNPs ranged from 96.8% (KORA F4) to 99% (SAPHIR). Duplicate genotyping was performed in at least 14% of the subjects in each study with a concordance of 95–100% (median 100%). In the Ogliastra Genetic Park Replication Study (n = 3000) *de novo* genotyping was conducted on a 7900HT Fast Real-Time PCR System (Applied Biosystems, Foster City, USA), with a mean call rate of 99.4% and 100% concordance of SNPs genotyped in duplicate.

### Between-strata analyses for candidate SNPs in replication samples

Twenty-nine SNPs, including the 6 novel loci reported in the current manuscript along with 23 previously confirmed to be associated with renal function [9], were tested for differential effects between the strata. The same Z statistics as described for discovery (above) was used and the Bonferroni-adjusted significance level was set to 0.10/29 = 0.003.

SNP-by-age interaction, for the one SNP showing significantly different effects between strata of age, was tested in the ARIC study by fitting a linear model on log(eGFRcrea) adjusted for sex, recruitment site, the first and the seventh genetic principal components (only these two were associated with the outcome at *P* value<0.05). Both the interaction term and the terms for the main effects of age and the SNP were included in the model.

### Power to assess between-strata effect difference

To assess genome-wide between-strata differences, with alpha = 5×10^−8^ and power = 80%, the maximum detectable difference was 0.025 when comparing nonDM versus DM and 0.015 when comparing nonHTN versus HTN. Similarly, when testing for between-strata differences the 29 known and new loci (Bonferroni-corrected alpha = 0.003) in the combined sample (n = ∼125,000 in nonDM and n = ∼13,000 in DM) we had 80% power to detect differences as large as 0.035.

### Look-up in African Americans (CARe)

For each of the 6 lead SNPs identified in our European ancestry samples, we extracted eGFR association statistics from a genome-wide study in the CARe African ancestry consortium [12]. We further investigated potential allelic heterogeneity across ethnicities by examining the 250 kb flanking region surrounding each lead SNP to determine whether other SNPs with stronger associations exist in each region. A SNP with the smallest association *P* value with MAF>0.03 was considered the top SNP in the African ancestry sample. We defined statistical significance of the identified lead SNP in African ancestry individuals based on a region-specific Bonferroni correction. The number of independent SNPs was determined based on the variance inflation factor (VIF) with a recursive calculation within a sliding window of 50 SNPs and pairwise r^2^ of 0.2. These analyses were performed using PLINK.

### Analyses of related phenotypes

For each replicating SNP, we obtained association results for urinary albumin-to-creatinine ratio and microalbuminuria from our previous genome-wide association analysis [20], and for blood pressure and myocardial infarction from genome-wide association analysis from the ICBP [21] and CARDIoGRAM [22] consortia, respectively.

### eSNP analysis

Significant renal SNPs were searched against a database of expression SNPs (eSNP) including the following tissues: fresh lymphocytes [36], fresh leukocytes [37], leukocyte samples in individuals with Celiac disease [38], lymphoblastoid cell lines (LCL) derived from asthmatic children [39], HapMap LCL from 3 populations [40], a separate study on HapMap CEU LCL [41], peripheral blood monocytes [42], [43], adipose [44], [45] and blood samples [44], 2 studies on brain cortex [42], [46], 3 large studies of brain regions including prefrontal cortex, visual cortex and cerebellum (Emilsson, personal communication), liver [45], [47], osteoblasts [48], skin [49] and additional fibroblast, T cell and LCL samples [50]. The collected eSNP results met criteria for statistical significance for association with gene transcript levels as described in the original papers.

A second expression analysis of 81 biopsies from normal kidney cortex samples was performed as described previously [51], [52]. Genotyping was performed using Affymetrix 6.0 Genome-wide chip and called with GTC Software (Affymetrix). For eQTL analyses, expression probes (Affymetrix U133set) were linked to SNP probes with >90% call-rate using RefSeq annotation (Affymetrix build a30). *P* values for eQTLs were calculated using linear multivariable regression in both cohorts and then combined using Fisher's combined probability test (see also [52]). Pairwise LD was calculated using SNAP [53] on the CEU HapMap release 22.

### Zebrafish functional experiments

Zebrafish were maintained according to established IACUC protocols. Briefly, we injected zebrafish embryos with newly designed (mpped2, ddx1) or previously validated (casp9 [54]) morpholino antisense oligonucleotides (MO, GeneTools, Philomath OR) at the one-cell stage at various doses. We fixed embryos in 4% PFA at the appropriate stages for in situ hybridization (http://zfin.org/ZFIN/Methods/ThisseProtocol.html). Different anatomic regions of the kidney were visualized using a panel of 4 established markers: *pax2a* (global kidney marker) [15], *nephrin* (podocyte marker) [16], *slc20a1a* (proximal tubule) [17], and *slc12a3* (distal tubule marker) [17]. Abnormalities in gene expression were independently scored by two investigators. We compared the number of abnormal morphant embryos to control embryos, injected with a standard control MO designed by GeneTools, with the Fisher's exact test, at the Bonferroni-corrected significance level of 0.0125, i.e.: 0.05/4 markers. We documented the development of gross edema at 4 and 6 days post-fertilization in live embryos.

We performed dextran clearance experiments following previously described protocols [55]. Briefly, 80 hours after MO injection, we anesthetized embryos in 4 mg/ml Tricaine in embryo water (1∶20 dilution), then positioned embryos on their back in a 1% agarose injection mold. We injected an equal volume of tetramethylrhodamine dextran (70,000 MW; Invitrogen) into the cardiac sinus venosus of each embryo. We then returned the embryos to fresh embryo water. Using fluorescence microscopy, we imaged the embryos at 2 hours post-injection (82 hpf) to demonstrate equal loading, then at 48 hours post-injection (128 hpf) to evaluate dextran clearance.

Embryos were injected with control, mpped2, or casp9 MOs at the one-cell stage. At 48 hpf, embryos were manually dechorionated, anesthetized in a 1∶20 dilution of 4 mg/ml Tricaine in embryo water, and oriented on a 1% agarose injection mold. As previously described [56], embryos were injected with equal volumes of 10 mg/ml gentamicin (Sigma) in the cardiac sinus venosus, returned to fresh embryo water, and subsequently scored for edema (prevalence, time of onset) over the next 3 days.

## Supporting Information

Figure S1 Flowchart of the project.
(TIF)

Figure S2 Genome-wide −log_10_ *P* values plot from stage 1 discovery meta-analysis. Plots show the discovery analysis of eGFRcrea in the overall group, with known loci [8], [9] highlighted in orange and novel loci highlighted in blue (A), and in strata of the main CKD risk factors (B, C, D, and E), with complementary groups being contrasted each other. The dotted line indicates the genome-wide significance threshold at *P* value = 5×10^−8^. The unmarked locus is *RNASEH*2C on chromosome 11, colored in gray despite genome-wide significance. The P value for the current stage 1 discovery for rs4014195 was 2.7×10^−9^. This locus previously did not replicate [9]; when we additionally considered our prior non-overlapping in silico and de novo replication data, the current stage 2 *P* value was 0.8832, yielding a combined stage 1+stage 2 *P* value of 2.6×10^−7^. Therefore, we did not submit this SNP for further replication.
(PDF)

Figure S3 Quantile-quantile plots of observed versus expected −log_10_ *P* values from the discovery analysis of eGFRcrea overall (A) and by strata of the main CKD risk factors (B). The orange line and its 95% confidence interval (shaded area) represent the null hypothesis of no association. In panel (A), results are compared when considering all SNPs (black dots) and when removing SNPs from loci that were already reported in previous GWAS [8], [9] (orange dots). The meta-analysis inflation factor λ is reported along with the discovery sample size. Individual-study minimum, maximum and median λs are also reported for comparison. Genomic-control correction was applied twice: on individual study results, before the meta-analysis, and on the meta-analysis results.
(PDF)

Figure S4 Regional association plots for the six new loci in the European ancestry discovery samples: (A) *MPPED2*; (B) *DDX1*; (C) *SLC47A1*; (D) *CASP9*; (E) *CDK12*; (F) *INO80*. −log_10_ *P* values are plotted versus genomic position(build 36). The lead SNP in each region is labeled. Other SNPs in each region are color-coded based on their LD to the lead SNP(LD based on the HapMap CEU, see color legend). Gene annotations are based on UCSC Genome Browser(RefSeq Genes, build 36) and arrows indicate direction of transcription. Graphs were generated using the stand-alone version of LocusZoom [57], version 1.1.
(PDF)

Figure S5 Forest plots of the six novel loci in the discovery phase.
(TIF)

Figure S6 Results from discovery meta-analysis of eGFRcrea for the six new loci: overall sample and all strata are considered. Reported is the effect size on log(eGFRcrea) and its 95% confidence interval. The stratum where the SNP was discovered is marked with a triangle for discovery based on meta-analysis *P* value or with a circle for discovery based on direction test.
(TIF)

Figure S7 Regional association plots for the six new loci in the African ancestry CARe samples: (A) *MPPED2*; (B) *DDX1*; (C) *SLC47A1*; (D) *CASP9*; (E) *CDK12*; (F) *INO80.* −log_10_ *P* values are plotted versus genomic position (build 36). The lead SNP in each region is labeled and identified by a blue arrow and blue *P* value. The SNP with the smallest *P* value in the region is indicated by a red arrow. Other SNPs in each region are color-coded based on their LD to the lead SNP (based on the HapMap YRI, see color legend). Gene annotation is based on UCSC Genome Browser (RefSeq Genes, build 36) and arrows indicate direction of transcription. Graphs were generated using the stand-alone version of LocusZoom [57], version 1.1.
(PDF)

Figure S8 Ddx1 knockdown does not affect kidney gene expression. (A–E) Uninjected control embryos show normal kidney development as demonstrated by in situ hybridization for the renal markers *pax2a* (A, B), *nephrin* (C), *slc20a1a* (D) and *slc12a3* (E). (F–J) *Ddx1* morpholino(MO)-injected embryos do not show significant changes in renal marker expression. (K) Number of observed abnormalities per number of embryos examined at 400 uM MO injection for renal gene expression analysis.
(TIF)

Figure S9 Casp9 and mpped2 knockdown embryos are more susceptible to gentamicin-induced kidney injury. Compared to control embryos (A), casp9 and mpped2 knockdown embryos develop edema at 103 hpf (C, E), suggestive of a renal defect. When injected with gentamicin, a nephrotoxin that reproducibly induces edema in control embryos (B), mpped2 and casp9 knockdown embryos develop edema earlier, more frequently, and in a more severe fashion (D, F). Whereas control embryos primarily develop cardiac edema, mpped2 and casp9 knockdown embryos display cardiac (arrowhead), ocular (black arrow), and visceral (white arrow) edema, demonstrating that mpped2 and casp9 knockdown predisposes embryos to kidney injury. (G) Quantification of edema prevalence in control, mpped2, and casp9 knockdown embryos 2, 22, and 55 hours post-injection (hpi) of gentamicin. These numbers are presented graphically in Figure 2X.
(TIF)

Figure S10 Comparison of the effect size on eGFRcrea and on eGFRcys for the lead SNPs of known and new loci. Results are based on the largest sample size available for each locus, i.e. the combined discovery and replication sample for the novel loci (N = 130,600), the discovery sample only for the known loci (N = 74,354). Sign of effect estimates has been changed to reflect the effects of the eGFRcrea lowering alleles. Original beta coefficients and their standard errors for the two traits can be downloaded from the File S1.
(TIF)

Figure S11 Odds ratios (ORs) and 95% confidence intervals of CKD and CKD45 for the lead SNPs of all known and new loci, sorted by decreasing OR of CKD.
(TIF)

File S1 Effect size on eGFRcrea and on eGFRcys for the lead SNPs of known and new loci.
(XLSX)

Table S1 Study-specific methods and full acknowledgments—discovery studies.
(DOC)

Table S2 Study-specific methods and full acknowledgments—replication studies and functional follow-up studies.
(DOC)

Table S3 Characteristics of stage 1 discovery studies.
(DOC)

Table S4 Study-specific genotyping information for stage 1 discovery studies.
(DOC)

Table S5 Characteristics of stage 2 replication studies.
(DOC)

Table S6 Study-specific genotyping information for stage 2 *in silico* replication studies.
(DOC)

Table S7 Top four SNPs from the CKD45 analysis.
(DOC)

Table S8 Loci identified by the test for differential effects between strata in the GWAS. Results are sorted by trait, group and chromosome. For each SNP, the *P* value of the test for difference between strata is reported.
(DOC)

Table S9 Imputation quality of replicated SNPs in all discovery and replication studies: median MACH-Rsq and interquartile range (IQR) are reported.
(DOC)

Table S10 Effects of novel and known loci on log(eGFRcrea) in the overall population.
(DOC)

Table S11 Genes nearest to loci associated with renal traits.
(DOC)

Table S12 Imputation Quality (MACH-Rsq) for the best SNPs in the African ancestry samples of the CARe consortium (1.00 refers to genotyped data).
(DOC)

Table S13 Baseline characteristics of the kidney biopsies for the eQTL analysis.
(DOC)

Table S14 Analysis of the new loci for eQTL status in meta-analysis of two cohorts of kidney biopsies.
(DOC)

Table S15 Association of novel and known loci with CKD and CKD45: Odds Ratios (OR), 95% confidence intervals (95%CI) and *P* values.
(DOC)

Table S16 Association between novel and known loci and log(eGFRcrea) in individuals without and with diabetes and test for difference between strata.
(DOC)

Table S17 Association between novel and known loci and log(eGFRcrea) in individuals without and with hypertension and test for difference between strata.
(DOC)

Table S18 Association between novel and known loci and log(eGFRcrea) in individuals younger and older than 65 years and test for difference between strata.
(DOC)

Table S19 Association between novel and known loci and log(eGFRcrea) in females and in males and test for difference between strata.
(DOC)

Table S20 Effects of novel loci on the logarithm of urinary albumin-to-creatinine ratio (log(UACR)) in the overall sample and by diabetes and hypertension status.
(DOC)

Table S21 Effects (log odds ratios) of novel loci on microalbuminuria (MA) in the overall sample and by diabetes and hypertension status.
(DOC)

Table S22 Association of novel loci with diastolic and systolic blood pressure in the ICBP consortium.
(DOC)

Table S23 Association of novel loci with myocardial infarction in the CARDIoGRAM consortium.
(DOC)
