# Supplementary figures and images for: Genome-Wide Association and Functional Follow-Up Reveals New Loci for Kidney Function
Source: PLoS Genet. 2012 Mar 29;8(3):e1002584. doi: 10.1371/journal.pgen.1002584 (PMC3315455; doi:10.1371/journal.pgen.1002584)

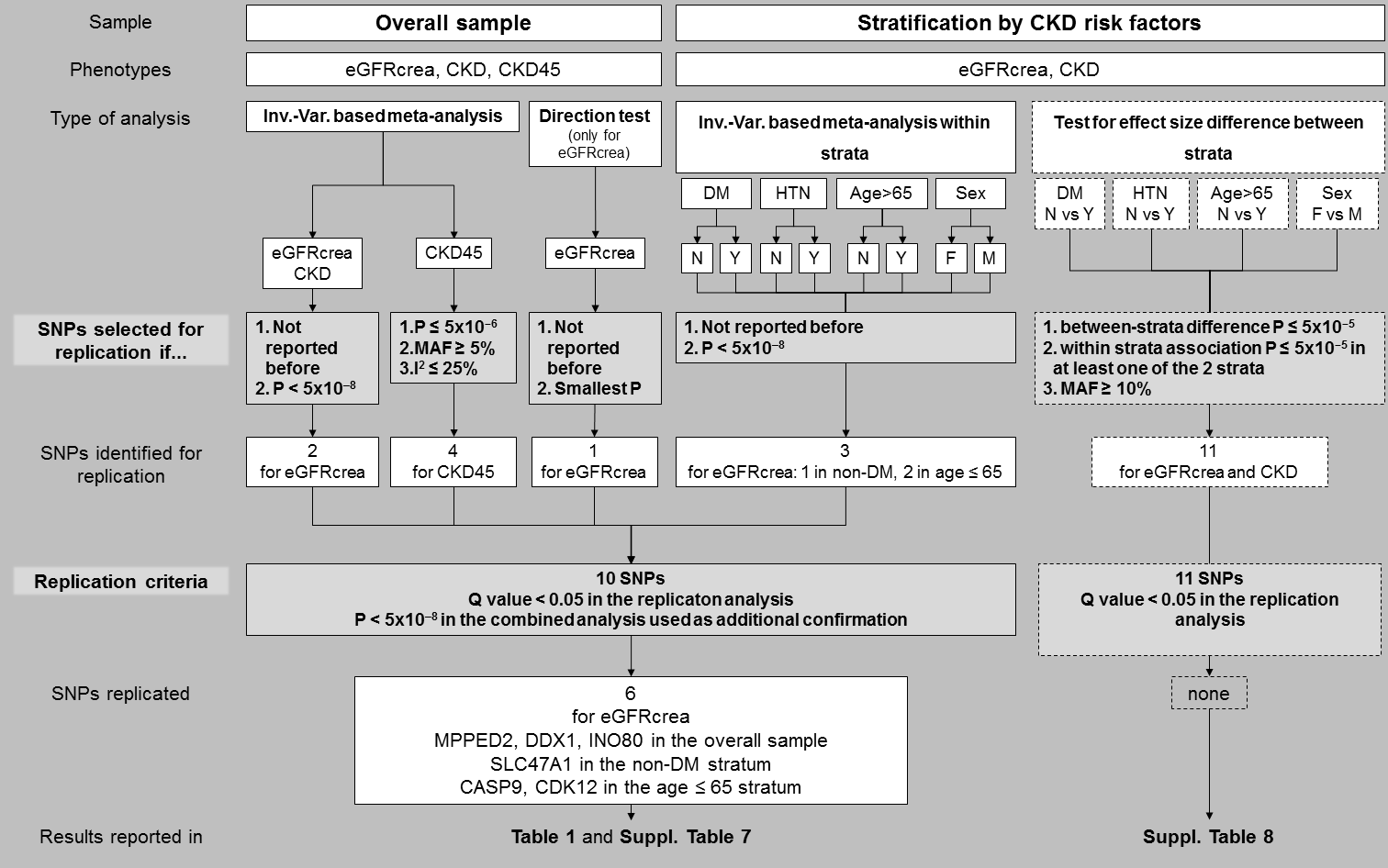

Supplement: Figure S1 — Flowchart of the project. (TIF) [file pgen.1002584.s001.tif]

**A**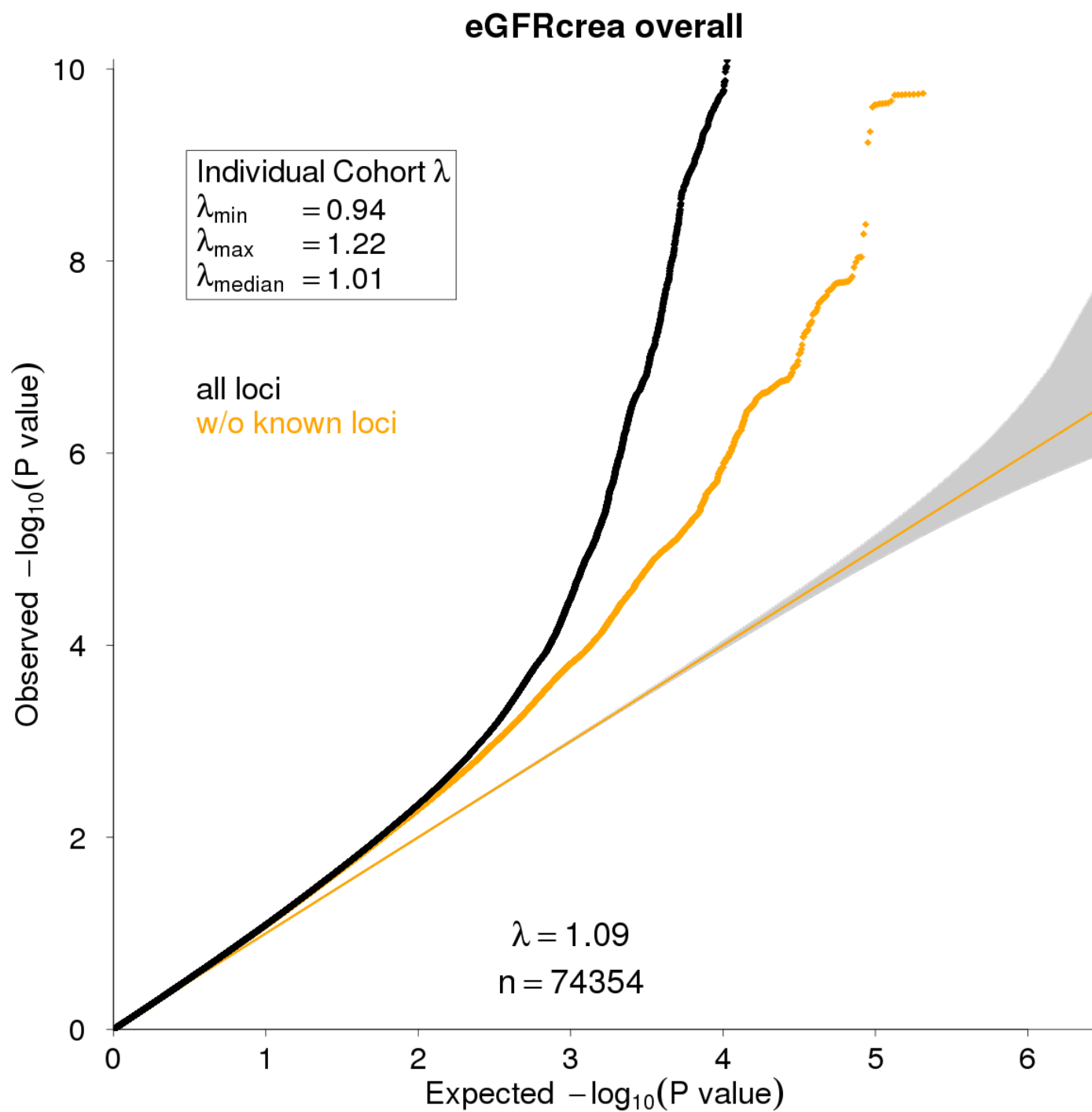

**B**

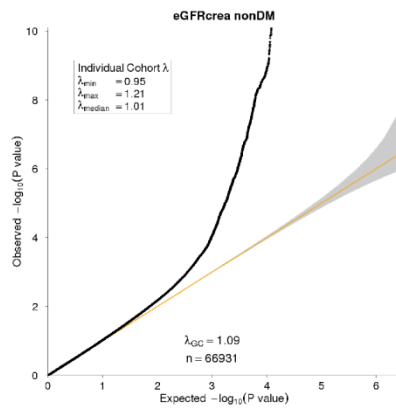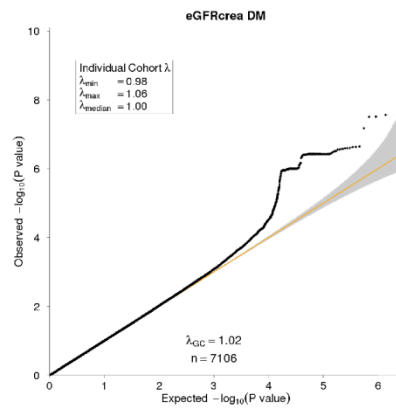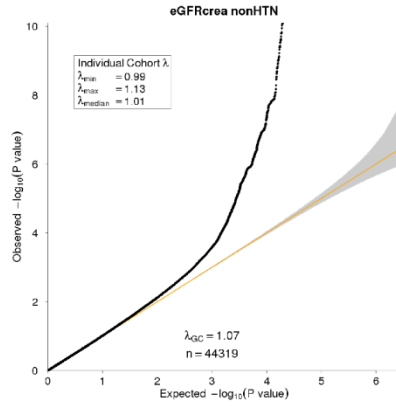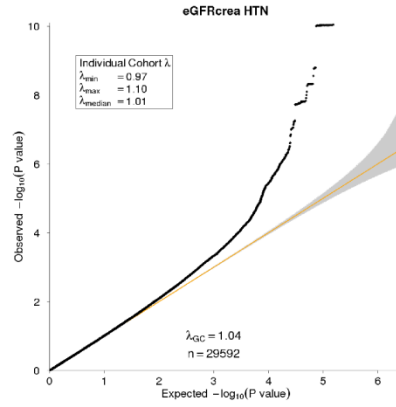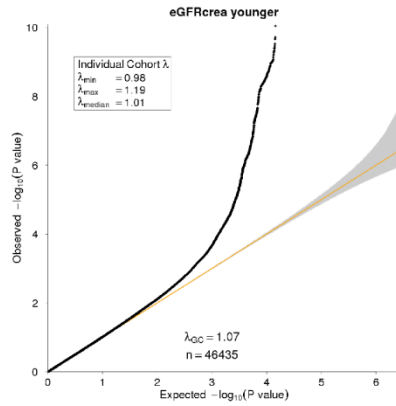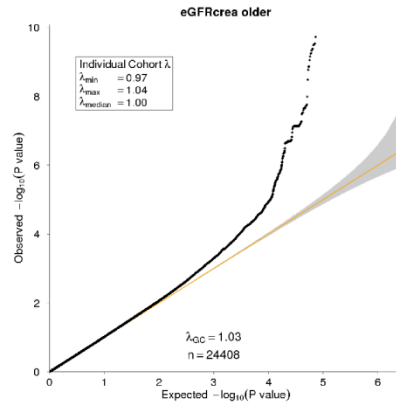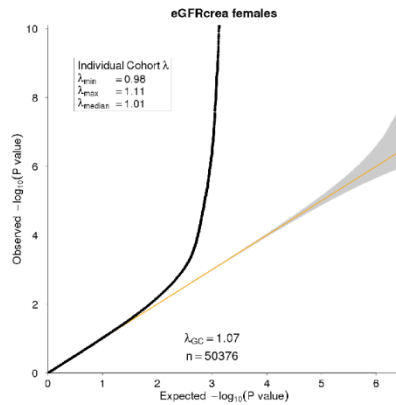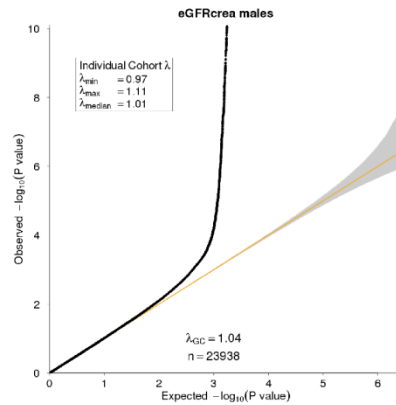

Supplement: Figure S3 — Quantile-quantile plots of observed versus expected −log10 P values from the discovery analysis of eGFRcrea overall (A) and by strata of the main CKD risk factors (B). The orange line and its 95% confidence interval (shaded area) represent the null hypothesis of no association. In panel (A), results are compared when considering all SNPs (black dots) and when removing SNPs from loci that were already reported in previous GWAS [8], [9] (orange dots). The meta-analysis inflation factor λ is reported along with the discovery sample size. Individual-study minimum, maximum and median λs are also reported for comparison. Genomic-control correction was applied twice: on individual study results, before the meta-analysis, and on the meta-analysis results. (PDF) [file pgen.1002584.s003.pdf]

# A

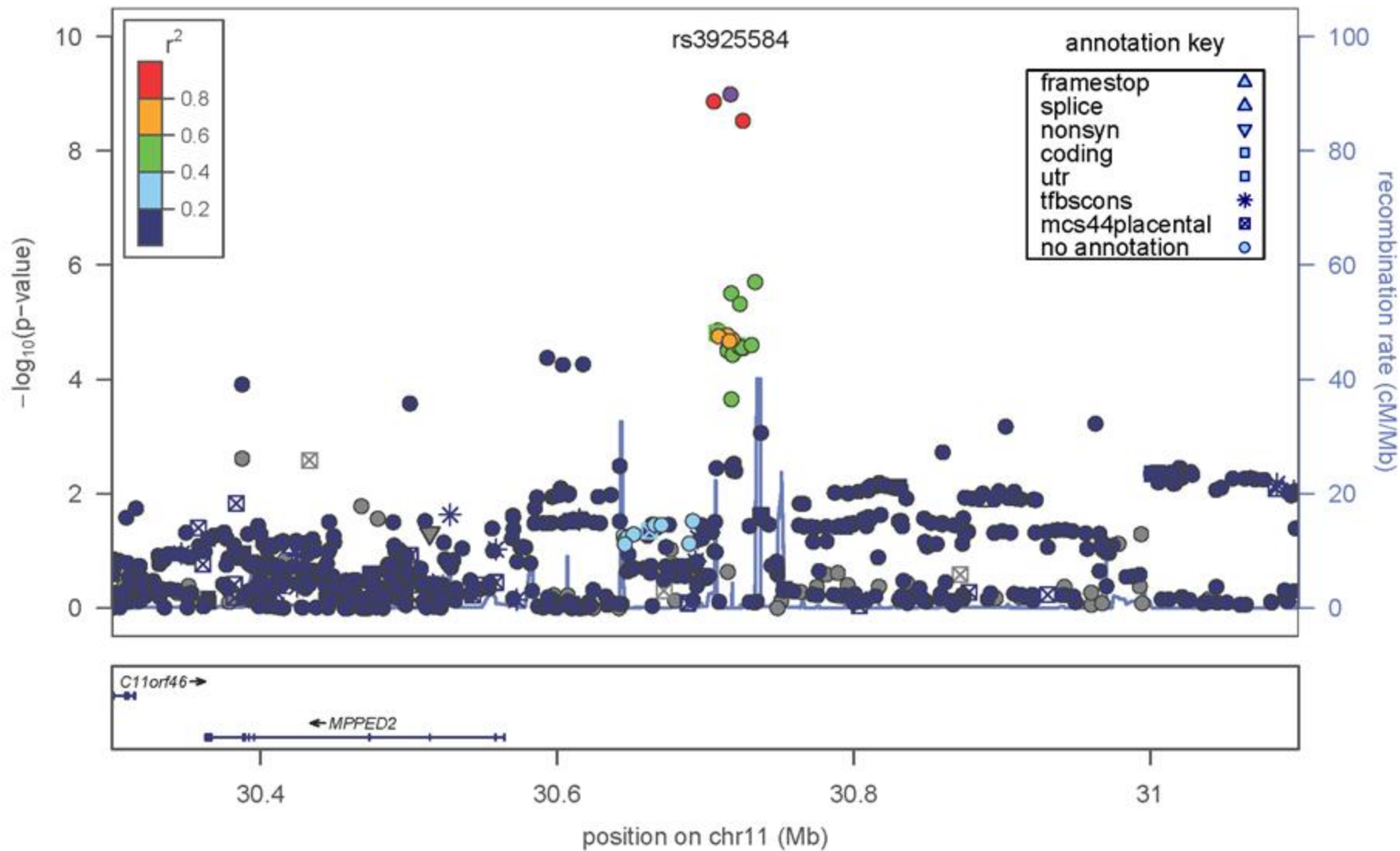

# B

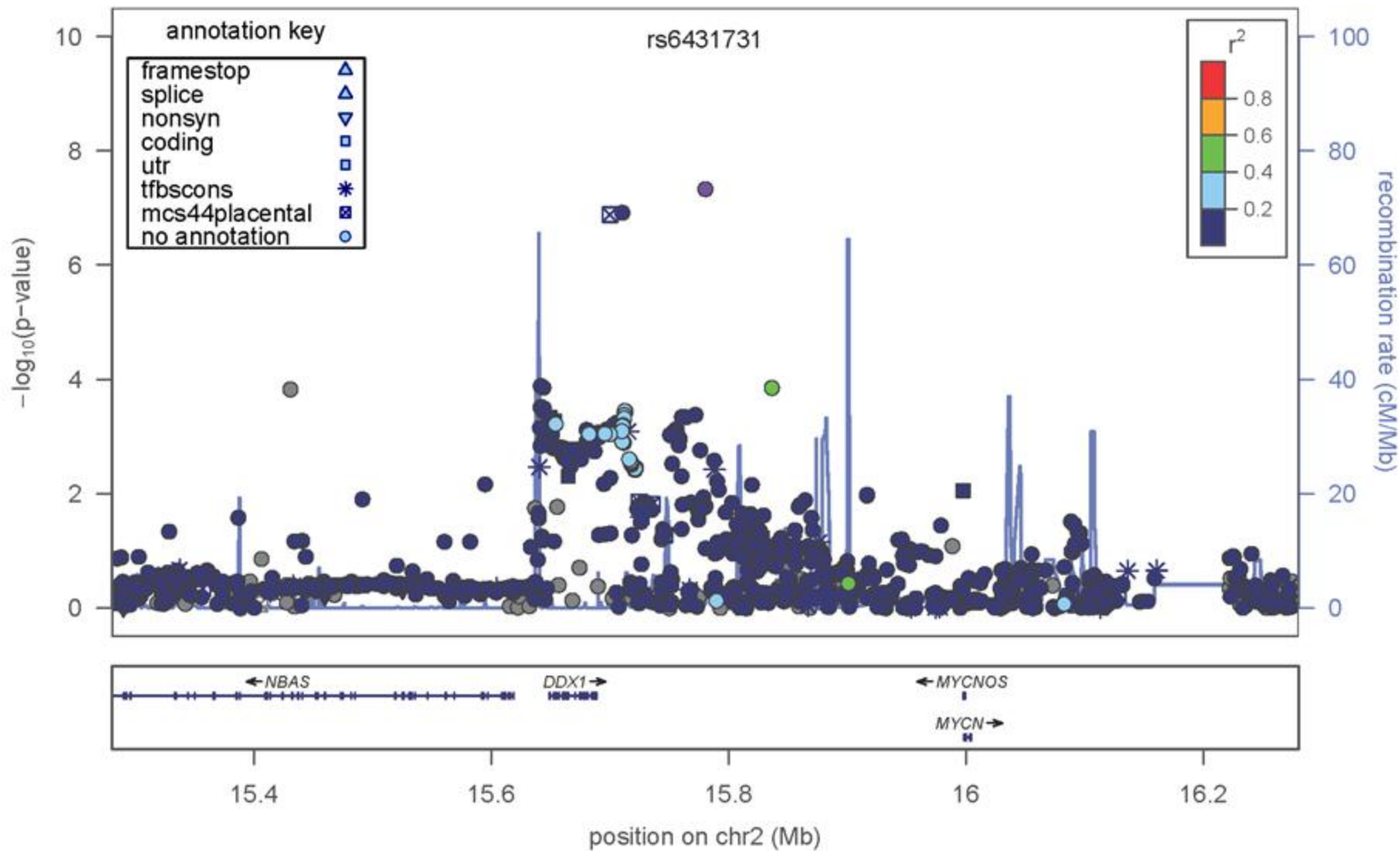

C

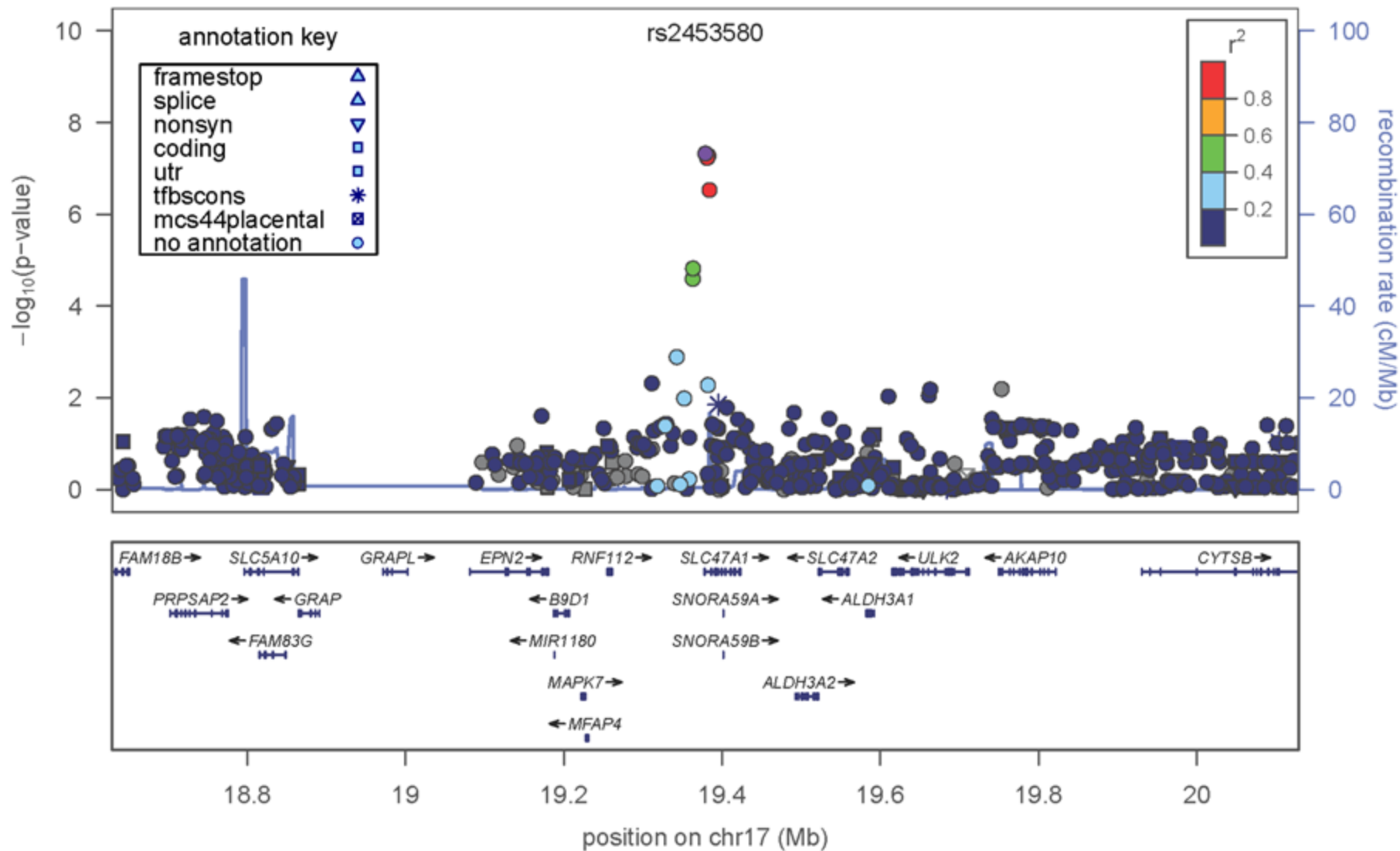

# D

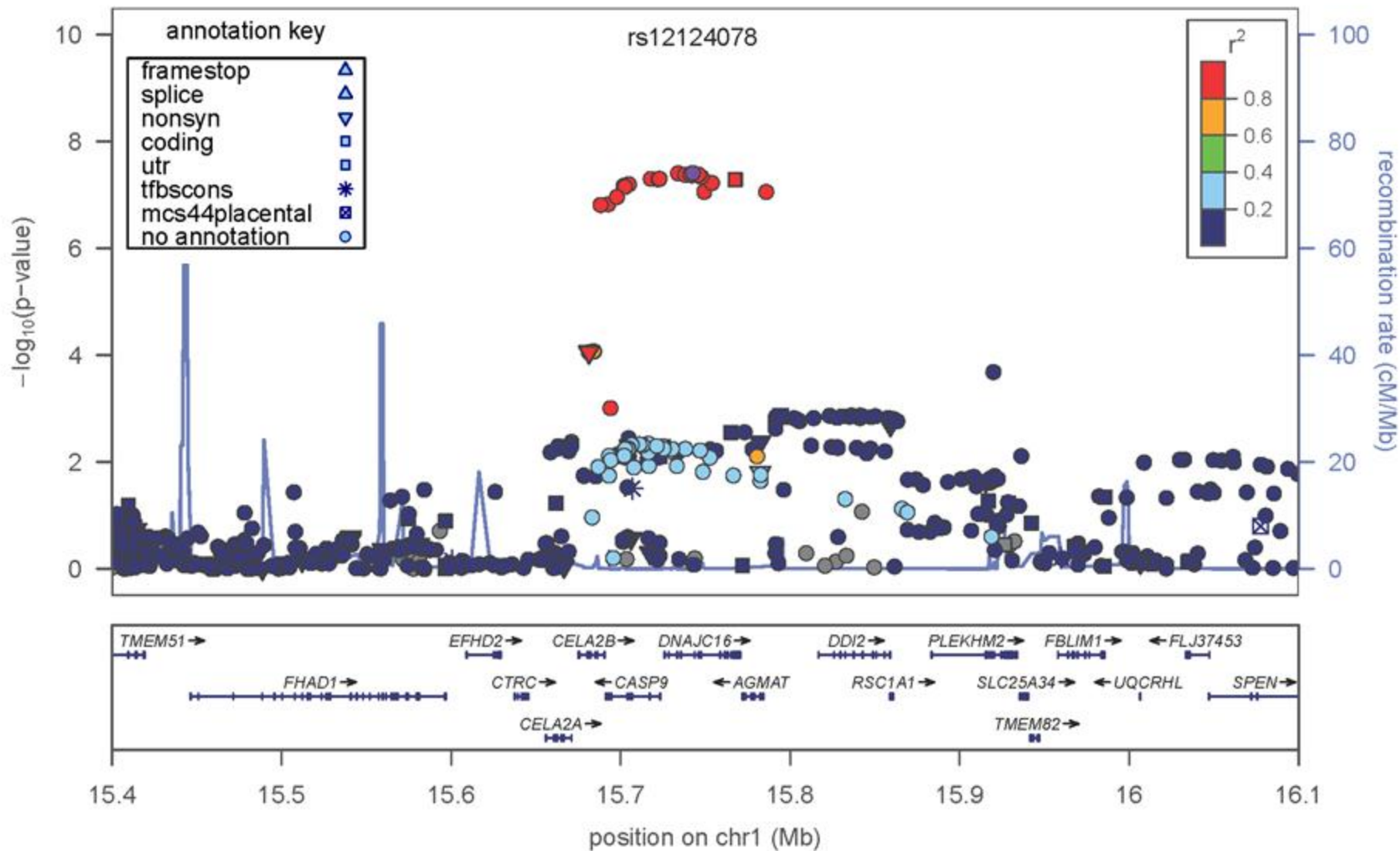

# E

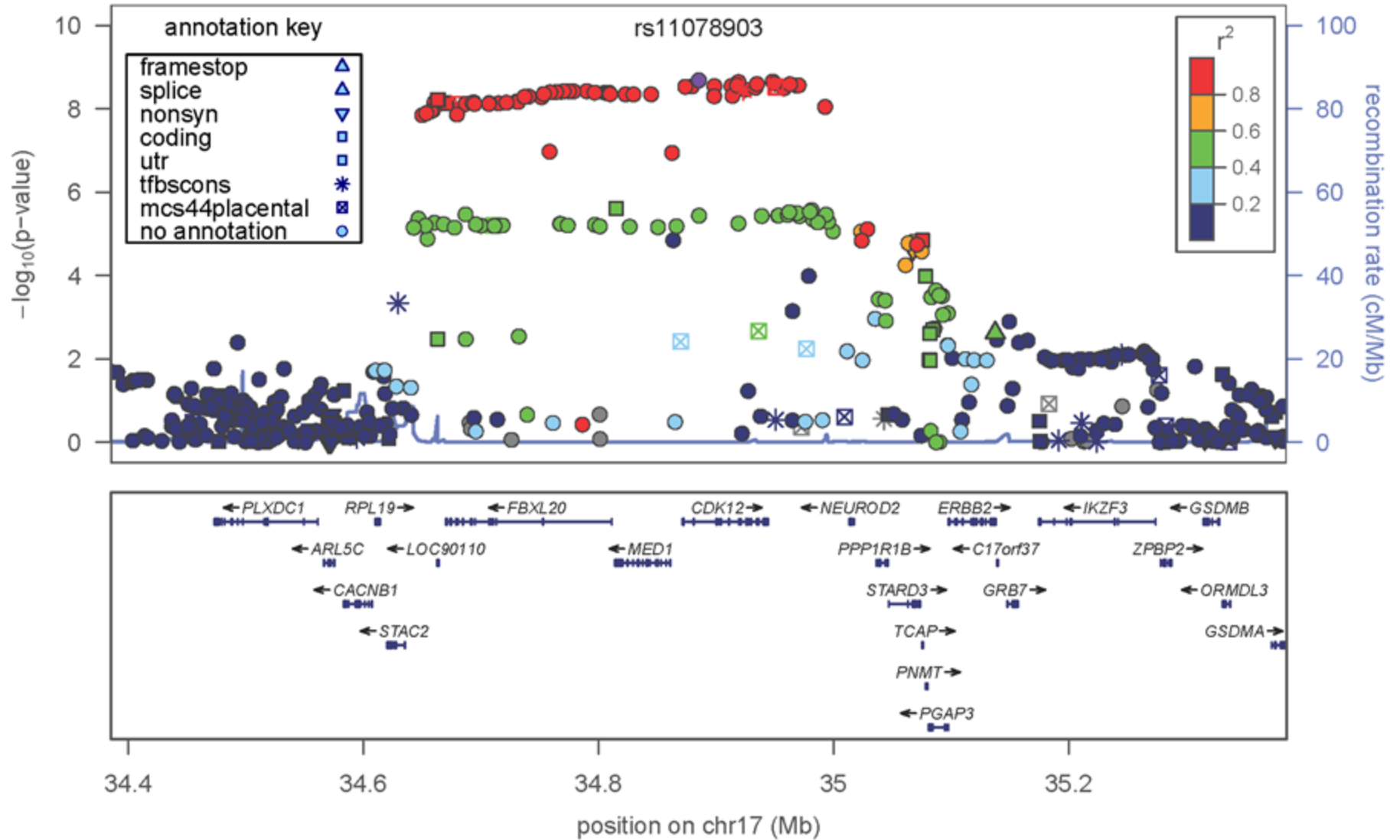

# F

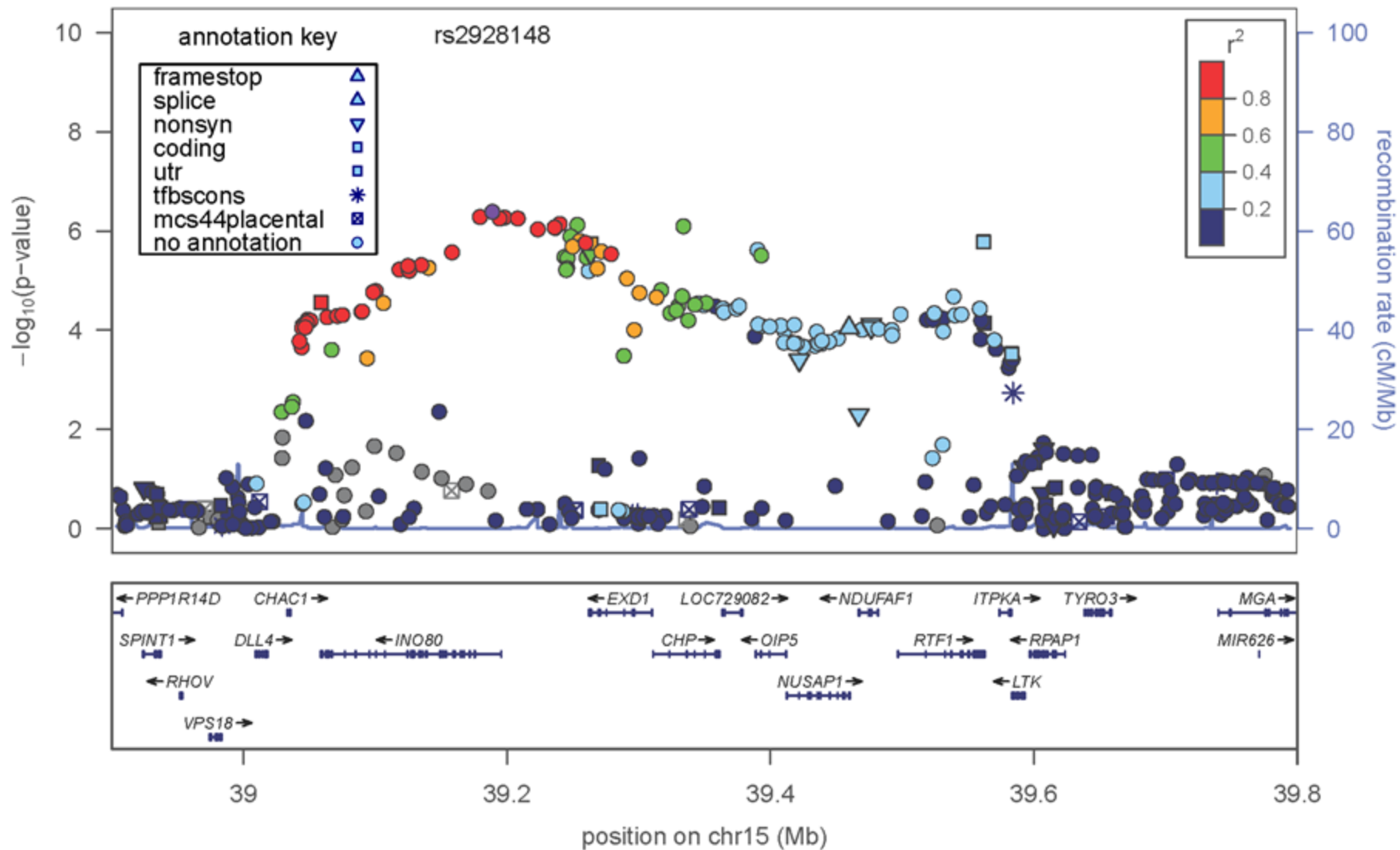

Supplement: Figure S4 — Regional association plots for the six new loci in the European ancestry discovery samples: (A) MPPED2; (B) DDX1; (C) SLC47A1; (D) CASP9; (E) CDK12; (F) INO80. −log10 P values are plotted versus genomic position(build 36). The lead SNP in each region is labeled. Other SNPs in each region are color-coded based on their LD to the lead SNP(LD based on the HapMap CEU, see color legend). Gene annotations are based on UCSC Genome Browser(RefSeq Genes, build 36) and arrows indicate direction of transcription. Graphs were generated using the stand-alone version of LocusZoom [57], version 1.1. (PDF) [file pgen.1002584.s004.pdf]

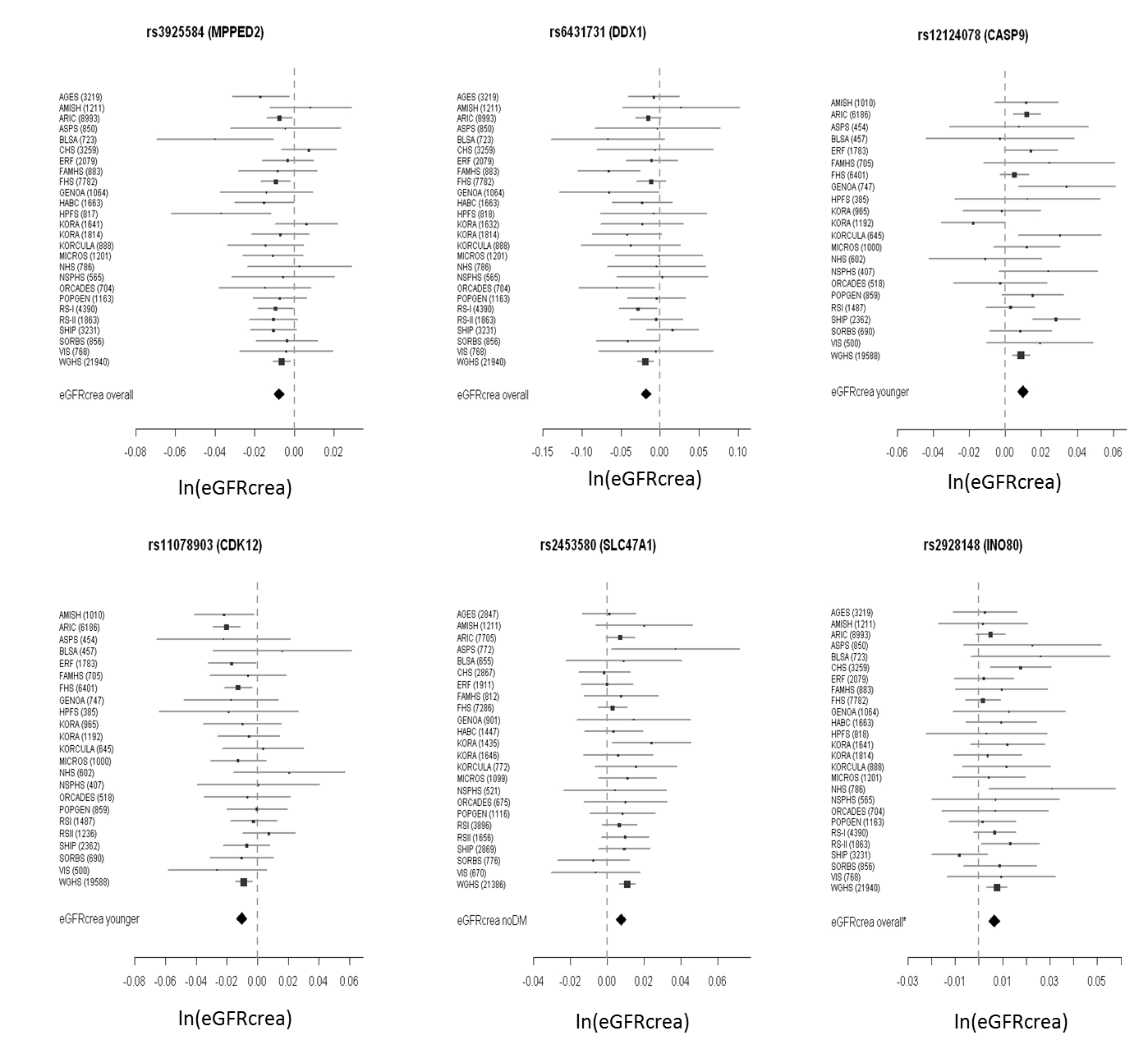

Supplement: Figure S5 — Forest plots of the six novel loci in the discovery phase. (TIF) [file pgen.1002584.s005.tif]

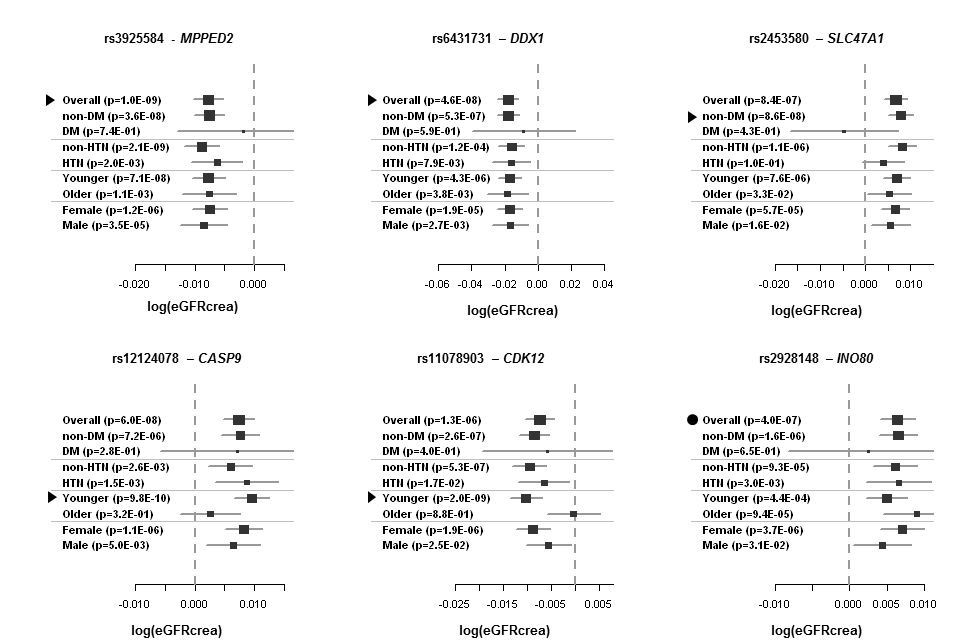

Supplement: Figure S6 — Results from discovery meta-analysis of eGFRcrea for the six new loci: overall sample and all strata are considered. Reported is the effect size on log(eGFRcrea) and its 95% confidence interval. The stratum where the SNP was discovered is marked with a triangle for discovery based on meta-analysis P value or with a circle for discovery based on direction test. (TIF) [file pgen.1002584.s006.tif]

# A

## rs3925584

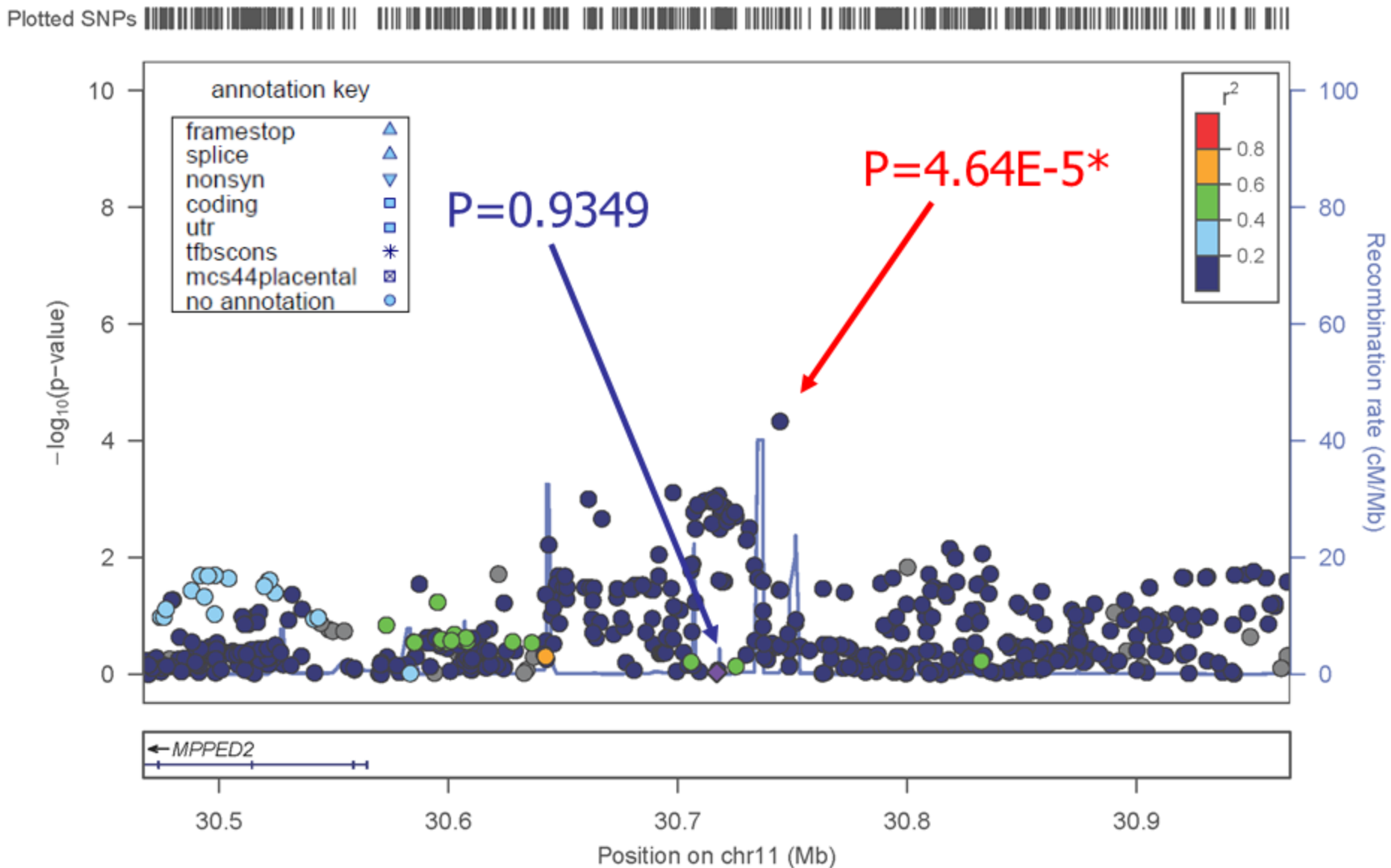

# B

## rs6431731

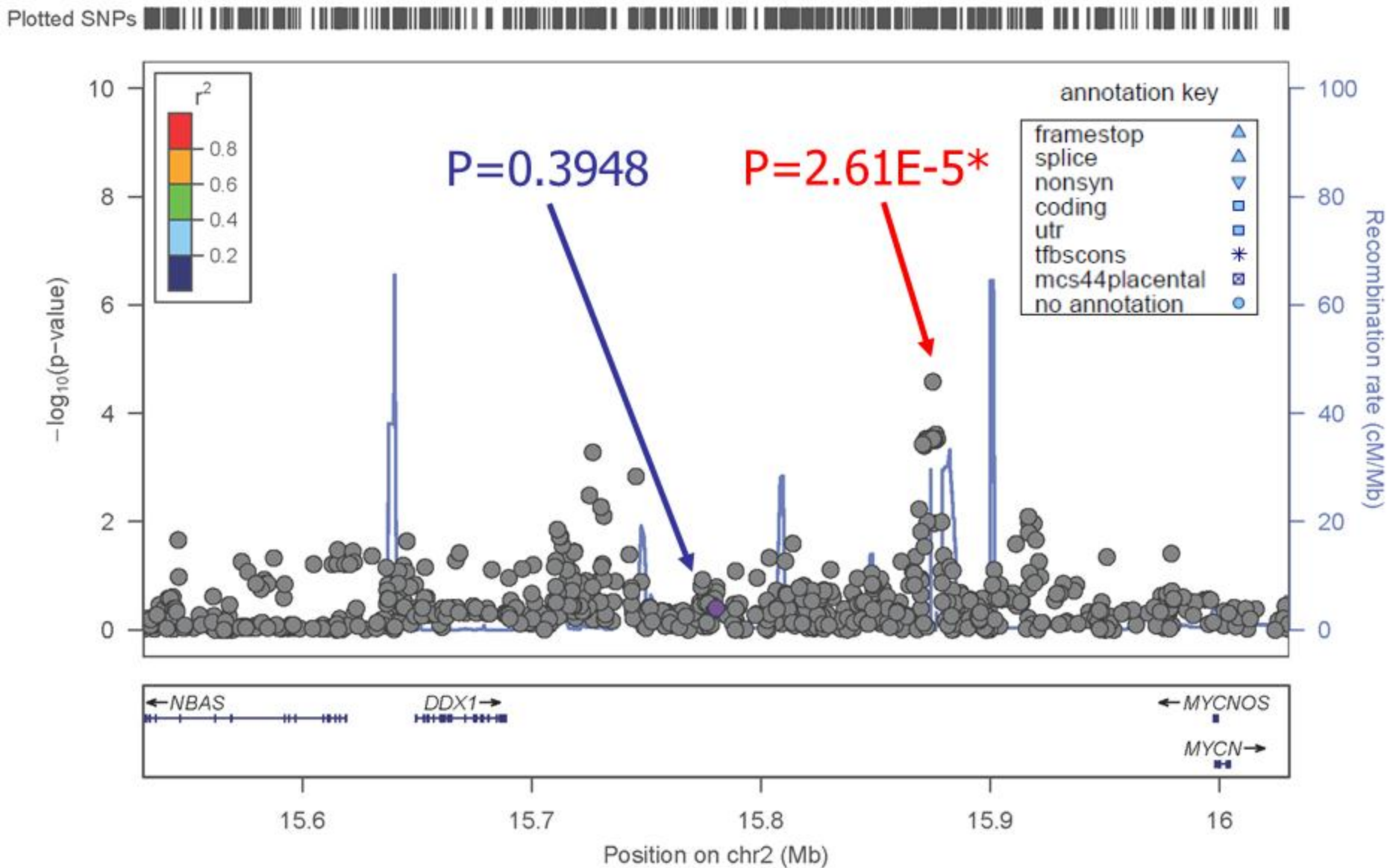

C

rs2453580

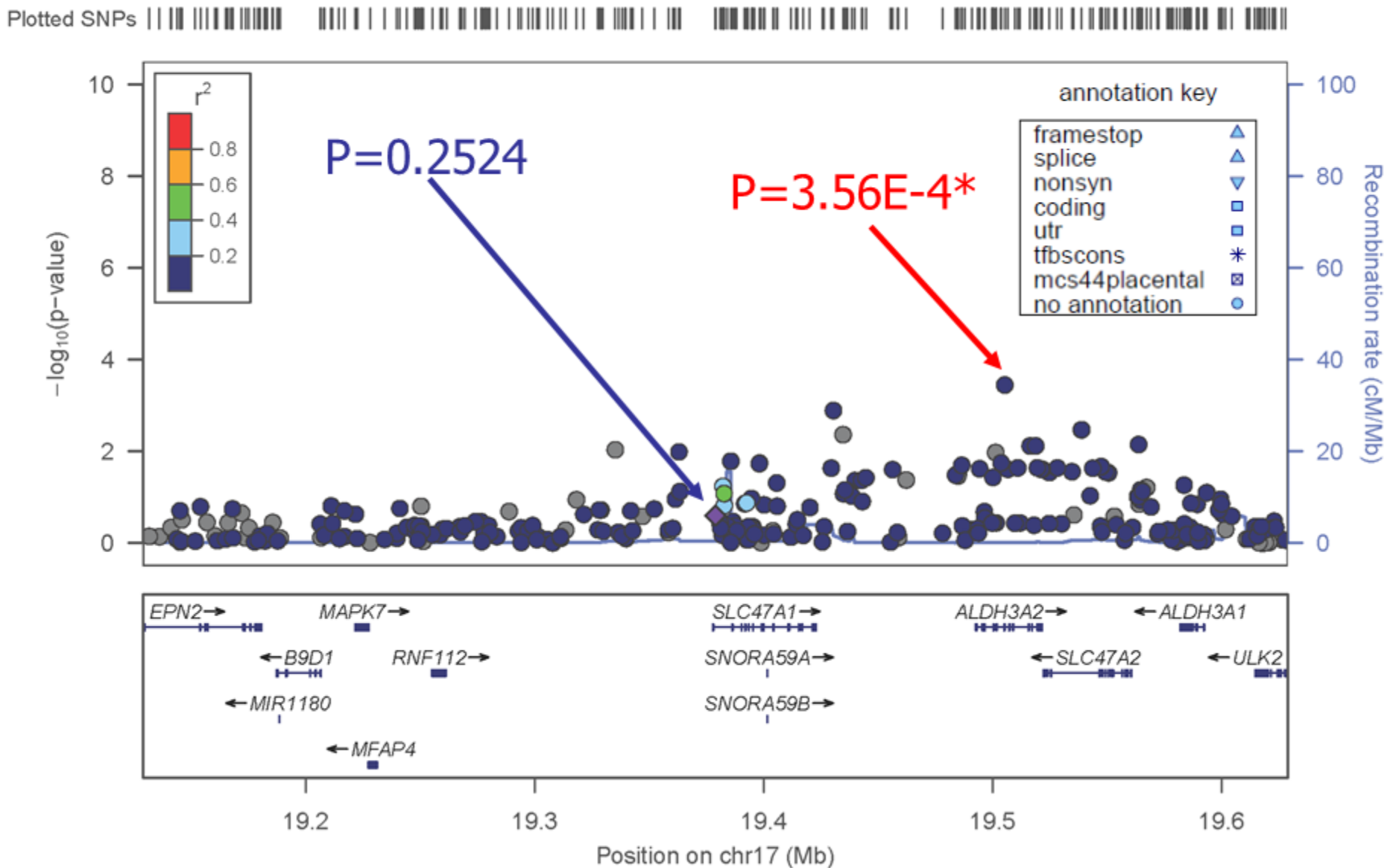

# D

## rs12124078

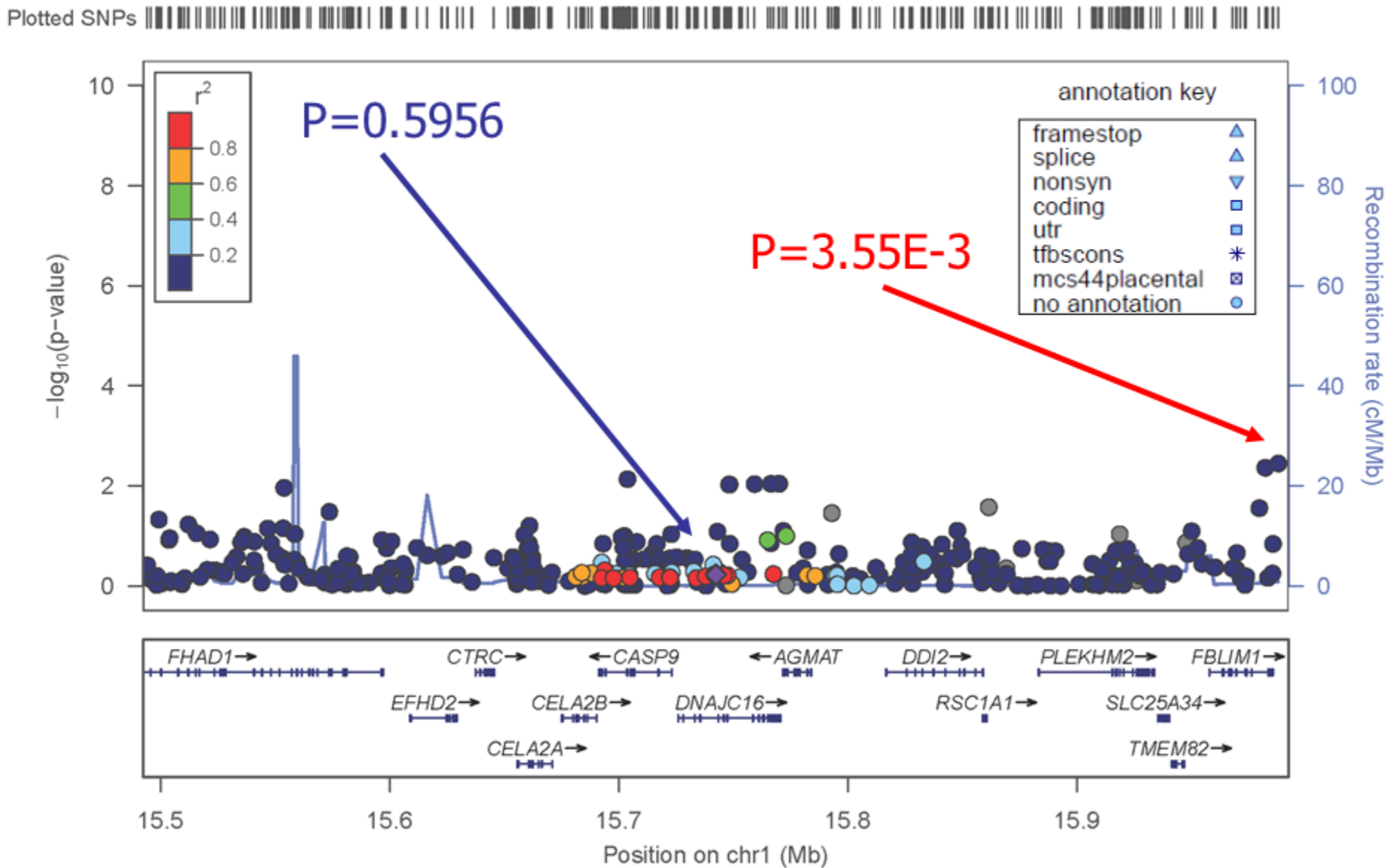

E

rs11078903

Plotted SNPs

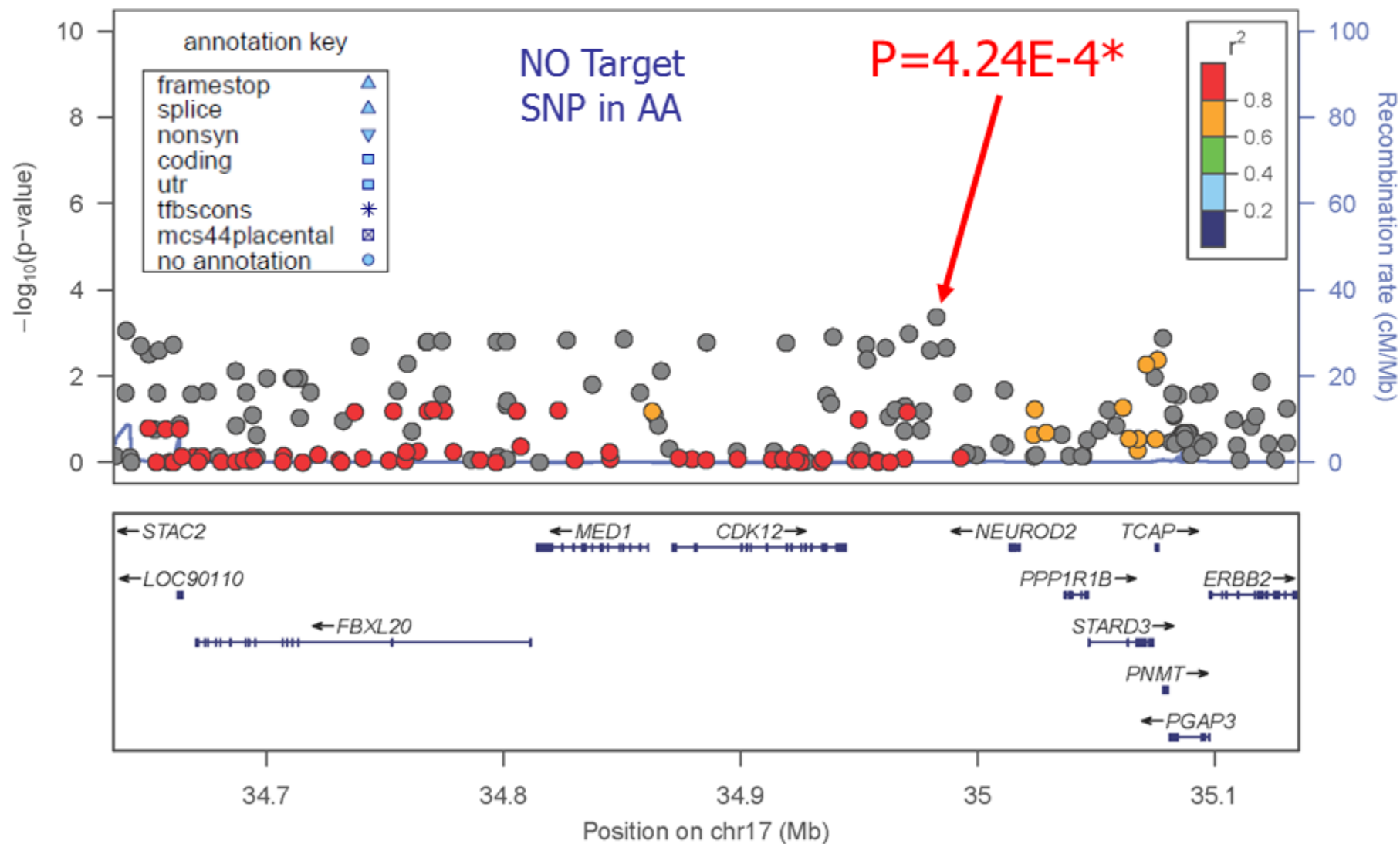

F

rs2928148

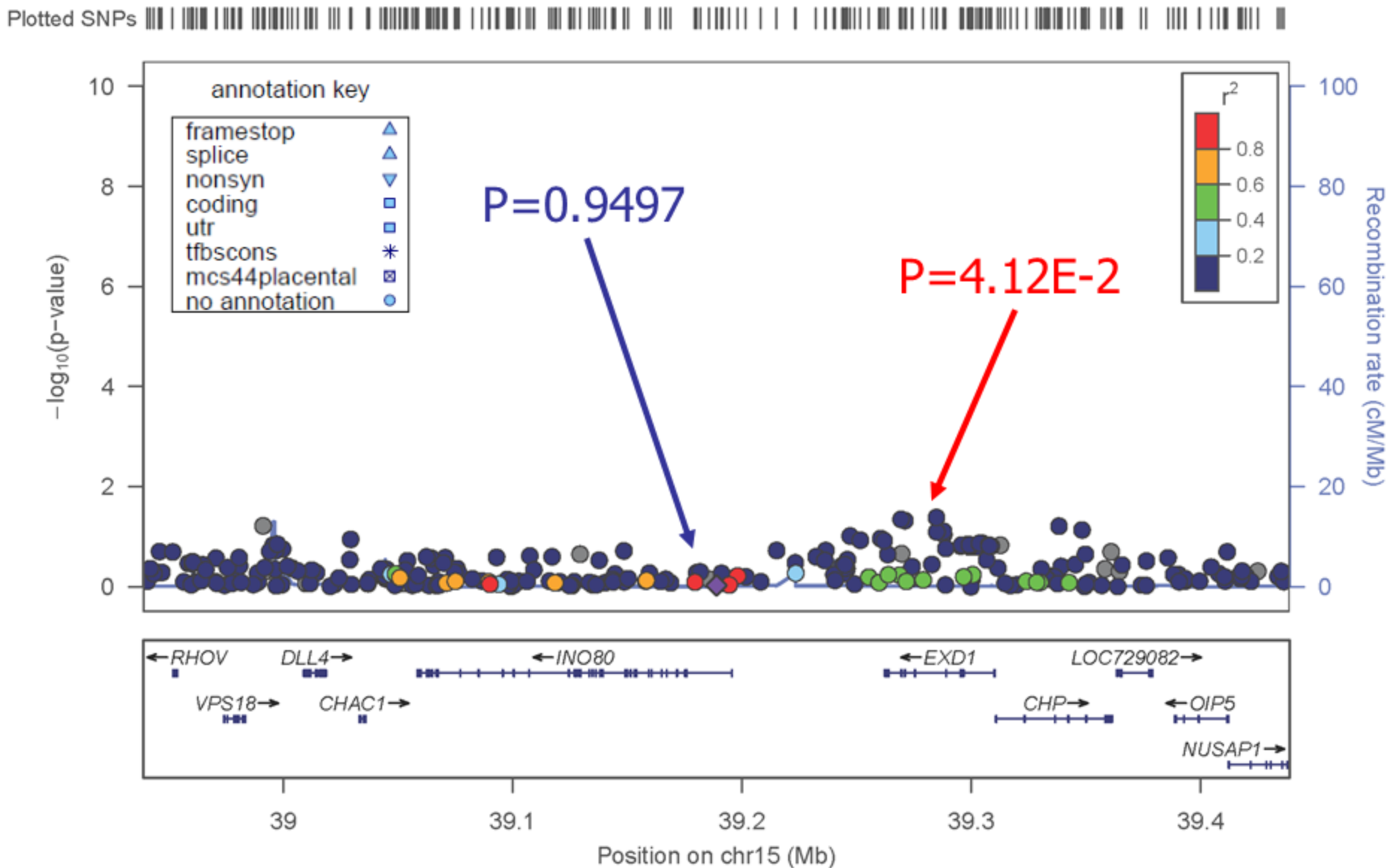

Supplement: Figure S7 — Regional association plots for the six new loci in the African ancestry CARe samples: (A) MPPED2; (B) DDX1; (C) SLC47A1; (D) CASP9; (E) CDK12; (F) INO80. −log10 P values are plotted versus genomic position (build 36). The lead SNP in each region is labeled and identified by a blue arrow and blue P value. The SNP with the smallest P value in the region is indicated by a red arrow. Other SNPs in each region are color-coded based on their LD to the lead SNP (based on the HapMap YRI, see color legend). Gene annotation is based on UCSC Genome Browser (RefSeq Genes, build 36) and arrows indicate direction of transcription. Graphs were generated using the stand-alone version of LocusZoom [57], version 1.1. (PDF) [file pgen.1002584.s007.pdf]

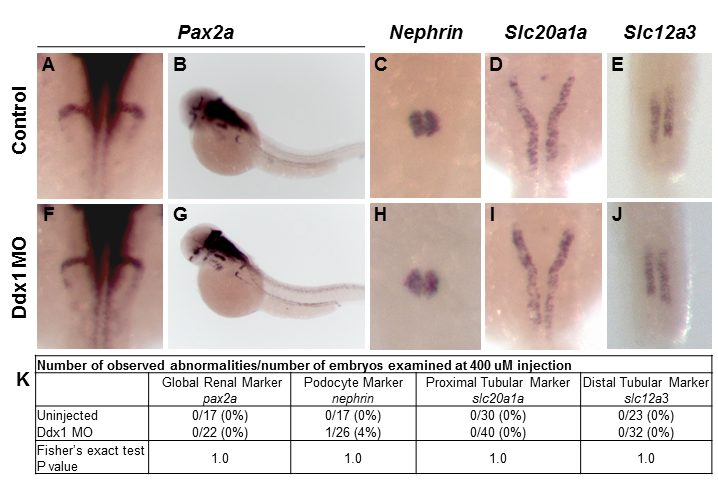

Supplement: Figure S8 — Ddx1 knockdown does not affect kidney gene expression. (A–E) Uninjected control embryos show normal kidney development as demonstrated by in situ hybridization for the renal markers pax2a (A, B), nephrin (C), slc20a1a (D) and slc12a3 (E). (F–J) Ddx1 morpholino(MO)-injected embryos do not show significant changes in renal marker expression. (K) Number of observed abnormalities per number of embryos examined at 400 uM MO injection for renal gene expression analysis. (TIF) [file pgen.1002584.s008.tif]

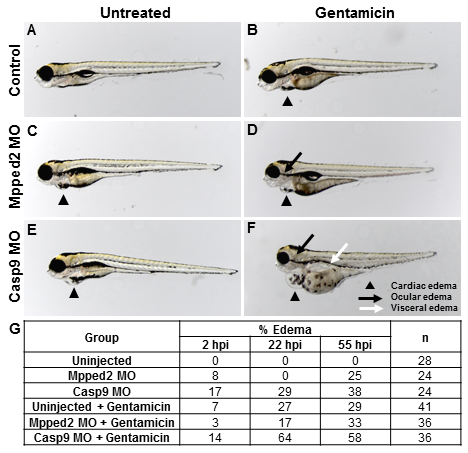

Supplement: Figure S9 — Casp9 and mpped2 knockdown embryos are more susceptible to gentamicin-induced kidney injury. Compared to control embryos (A), casp9 and mpped2 knockdown embryos develop edema at 103 hpf (C, E), suggestive of a renal defect. When injected with gentamicin, a nephrotoxin that reproducibly induces edema in control embryos (B), mpped2 and casp9 knockdown embryos develop edema earlier, more frequently, and in a more severe fashion (D, F). Whereas control embryos primarily develop cardiac edema, mpped2 and casp9 knockdown embryos display cardiac (arrowhead), ocular (black arrow), and visceral (white arrow) edema, demonstrating that mpped2 and casp9 knockdown predisposes embryos to kidney injury. (G) Quantification of edema prevalence in control, mpped2, and casp9 knockdown embryos 2, 22, and 55 hours post-injection (hpi) of gentamicin. These numbers are presented graphically in Figure 2X. (TIF) [file pgen.1002584.s009.tif]

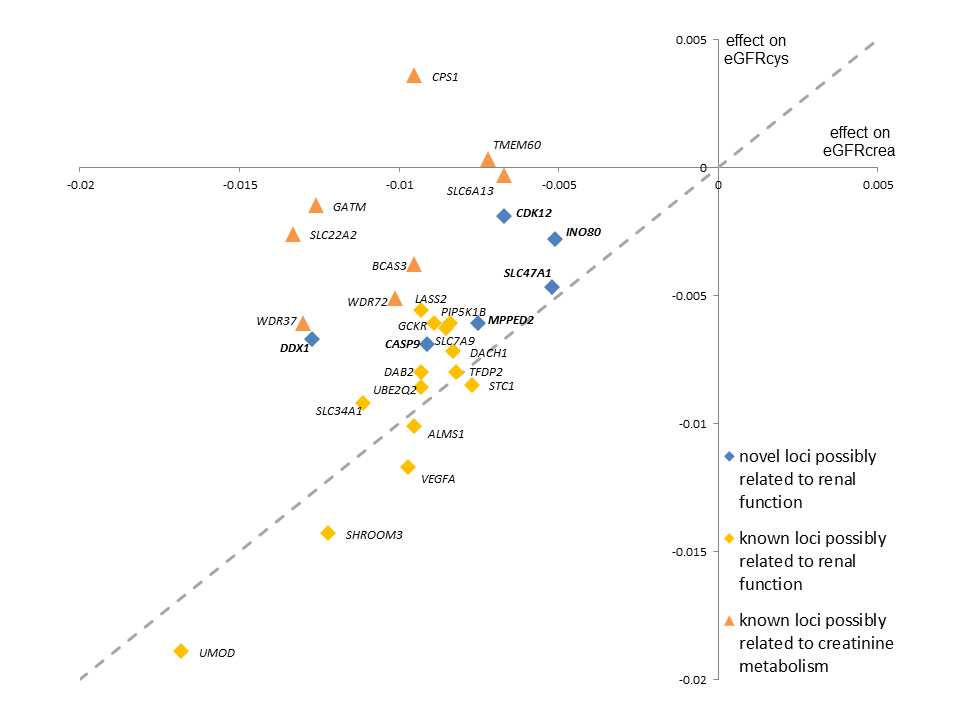

Supplement: Figure S10 — Comparison of the effect size on eGFRcrea and on eGFRcys for the lead SNPs of known and new loci. Results are based on the largest sample size available for each locus, i.e. the combined discovery and replication sample for the novel loci (N = 130,600), the discovery sample only for the known loci (N = 74,354). Sign of effect estimates has been changed to reflect the effects of the eGFRcrea lowering alleles. Original beta coefficients and their standard errors for the two traits can be downloaded from the File S1. (TIF) [file pgen.1002584.s010.tif]

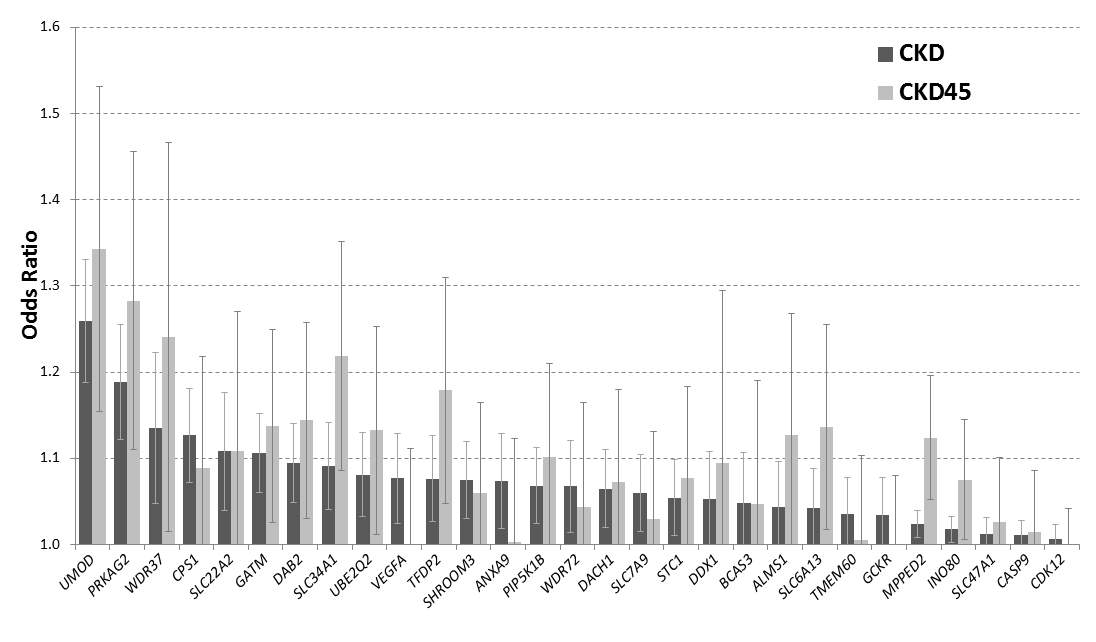

Supplement: Figure S11 — Odds ratios (ORs) and 95% confidence intervals of CKD and CKD45 for the lead SNPs of all known and new loci, sorted by decreasing OR of CKD. (TIF) [file pgen.1002584.s011.tif]
