# Supplementary material for: Genome-Wide Association and Functional Follow-Up Reveals New Loci for Kidney Function
Source: PLoS Genet. 2012 Mar 29;8(3):e1002584. doi: 10.1371/journal.pgen.1002584 (PMC3315455; doi:10.1371/journal.pgen.1002584)
Supplement: Figure S2 — Genome-wide −log10 P values plot from stage 1 discovery meta-analysis. Plots show the discovery analysis of eGFRcrea in the overall group, with known loci [8], [9] highlighted in orange and novel loci highlighted in blue (A), and in strata of the main CKD risk factors (B, C, D, and E), with complementary groups being contrasted each other. The dotted line indicates the genome-wide significance threshold at P value = 5×10^−8^. The unmarked locus is RNASEH2C on chromosome 11, colored in gray despite genome-wide significance. The P value for the current stage 1 discovery for rs4014195 was 2.7×10^−9^. This locus previously did not replicate [9]; when we additionally considered our prior non-overlapping in silico and de novo replication data, the current stage 2 P value was 0.8832, yielding a combined stage 1+stage 2 P value of 2.6×10^−7^. Therefore, we did not submit this SNP for further replication. (PDF) [file pgen.1002584.s002.pdf]

A

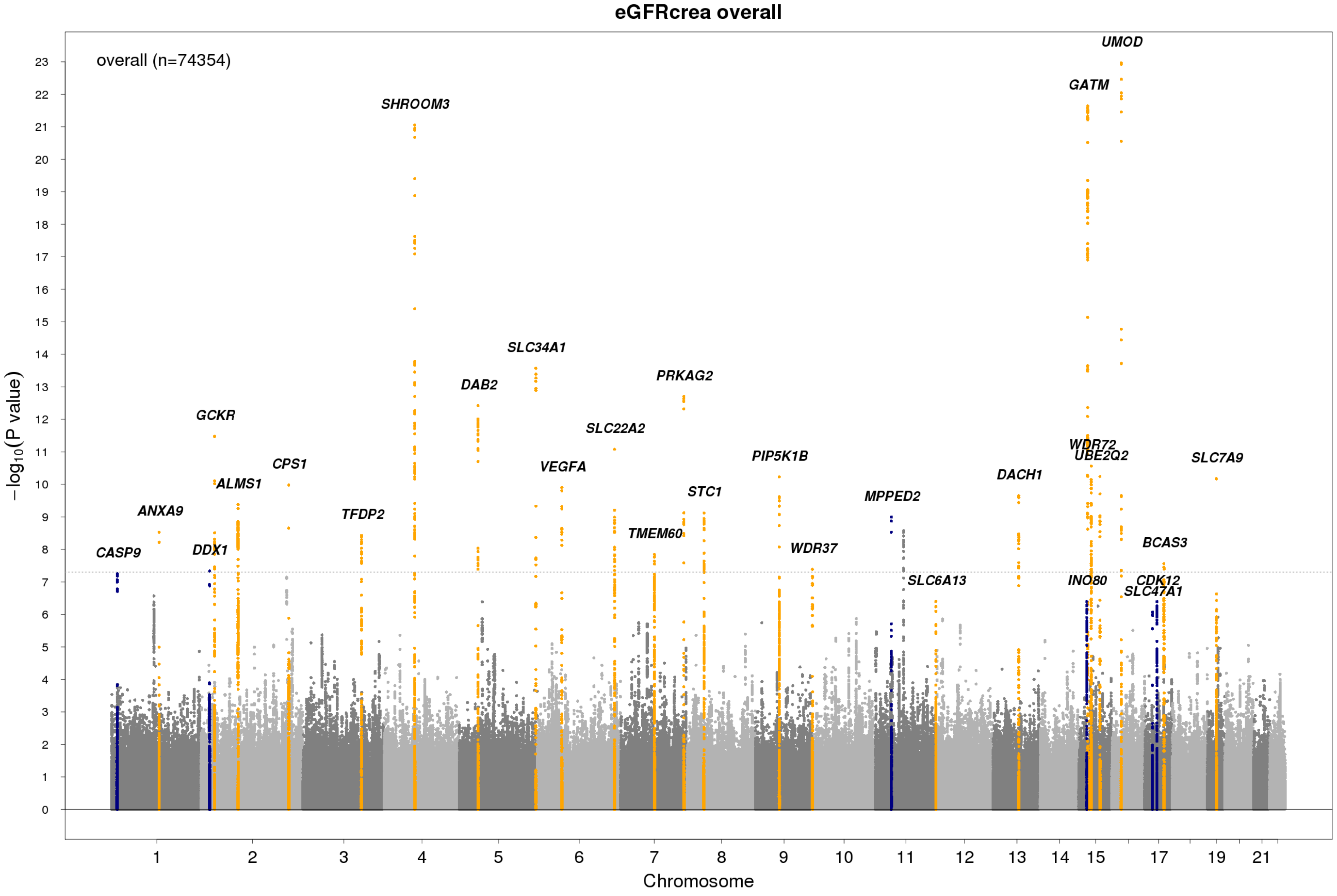

eGFRcrea overall
overall (n=74354)
UMOD
GATM
SHROOM3
SLC34A1
PRKAG2
DAB2
GCKR
SLC22A2
WDR72
UBE2Q2
SLC7A9
PIP5K1B
CPS1
VEGFA
DACH1
ALMS1
ANXA9
STC1
MPPED2
TFDP2
TMEM60
BCAS3
CASP9
DDX1
WDR37
SLC6A13
INO80
CDK12
SLC47A1
$-\log_{10}$(P value)
Chromosome
0
1
2
3
4
5
6
7
8
9
10
11
12
13
14
15
16
17
18
19
20
21
22
23
1
2
3
4
5
6
7
8
9
10
11
12
13
14
15
17
19
21

B

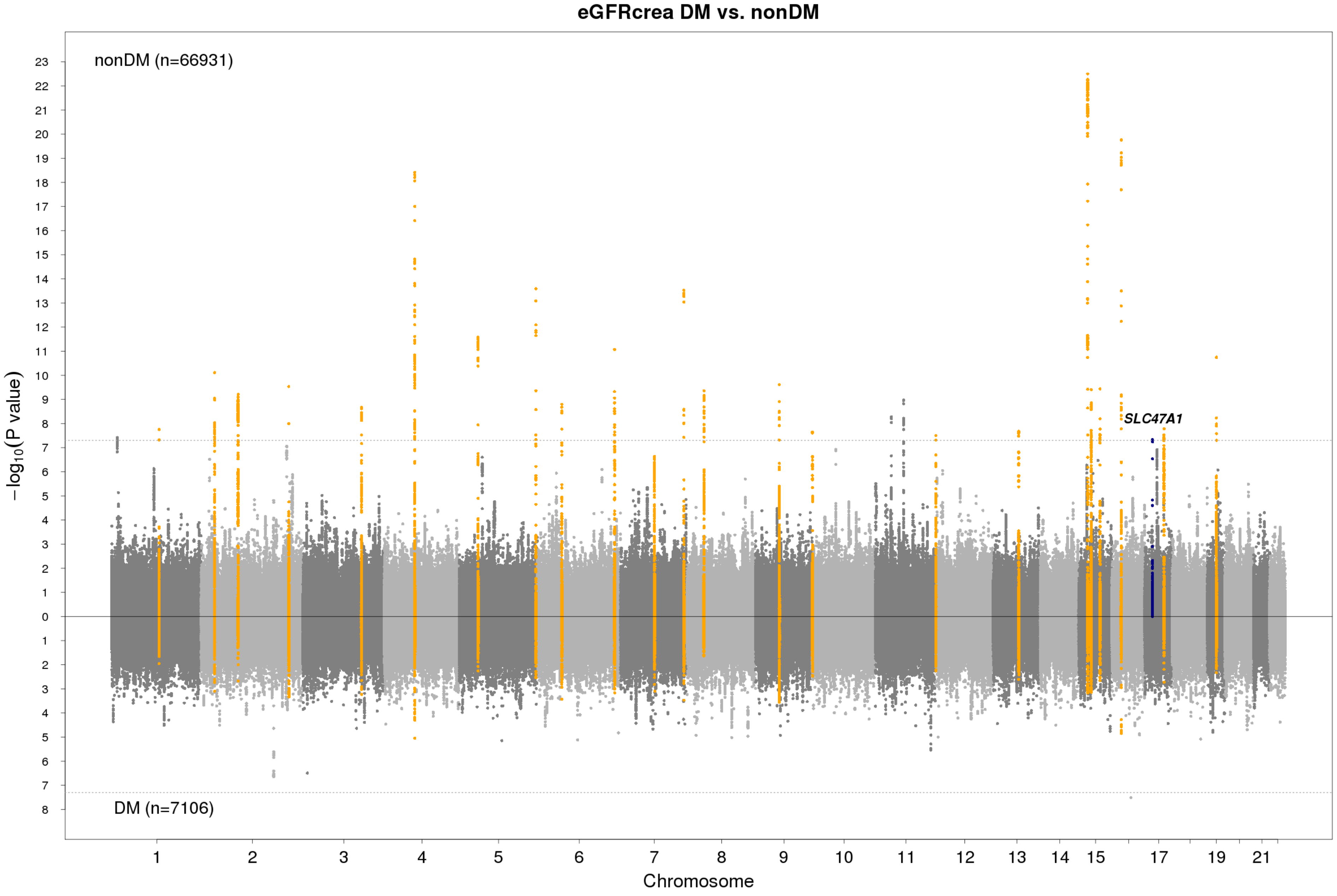
eGFRcrea DM vs. nonDM
nonDM (n=66931)
DM (n=7106)
SLC47A1
−log10(P value)
Chromosome
1
2
3
4
5
6
7
8
9
10
11
12
13
14
15
17
19
21

C

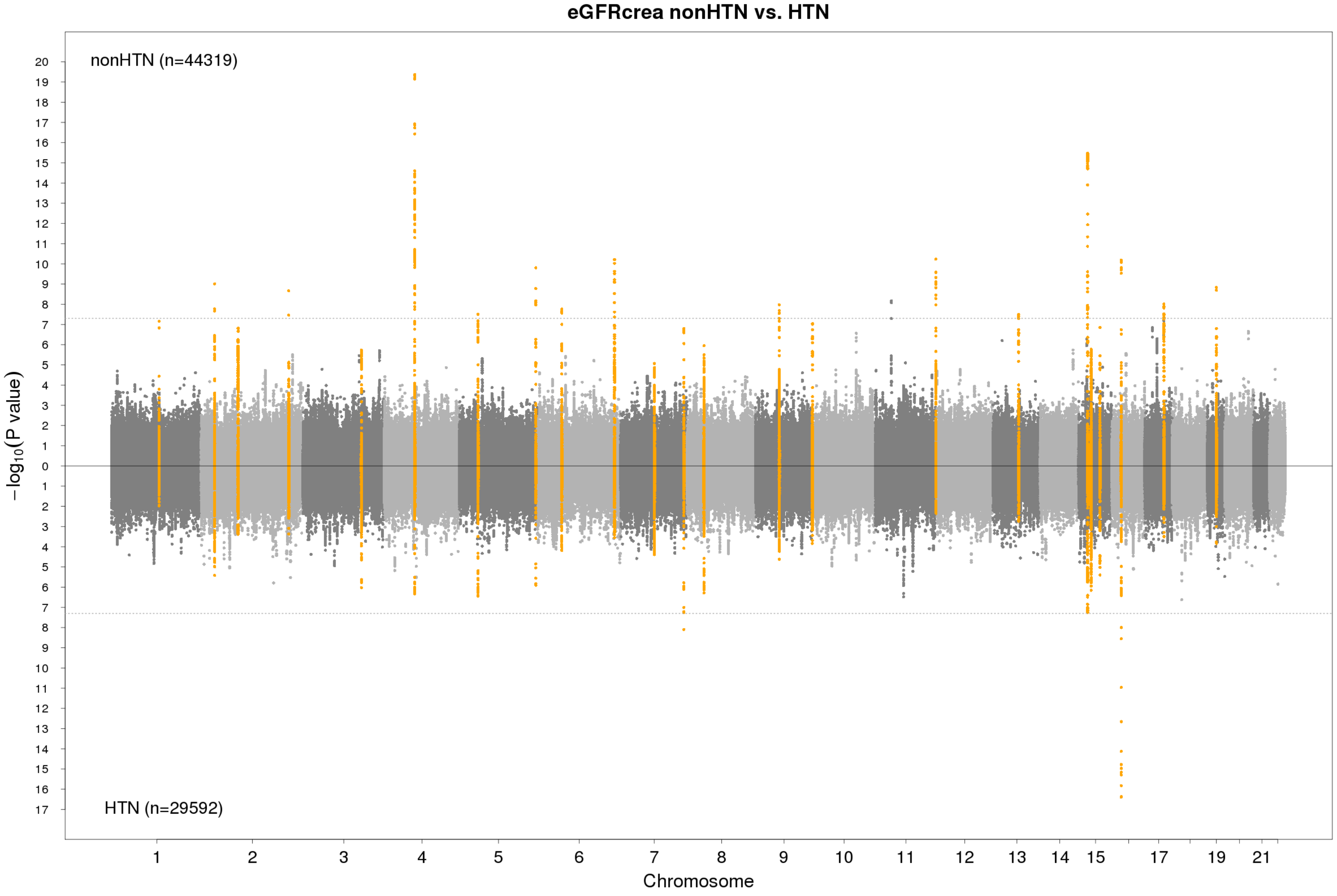
eGFRcrea nonHTN vs. HTN
nonHTN (n=44319)
HTN (n=29592)
−log10(P value)
Chromosome
20
19
18
17
16
15
14
13
12
11
10
9
8
7
6
5
4
3
2
1
0
1
2
3
4
5
6
7
8
9
10
11
12
13
14
15
16
17
1
2
3
4
5
6
7
8
9
10
11
12
13
14
15
17
19
21

D

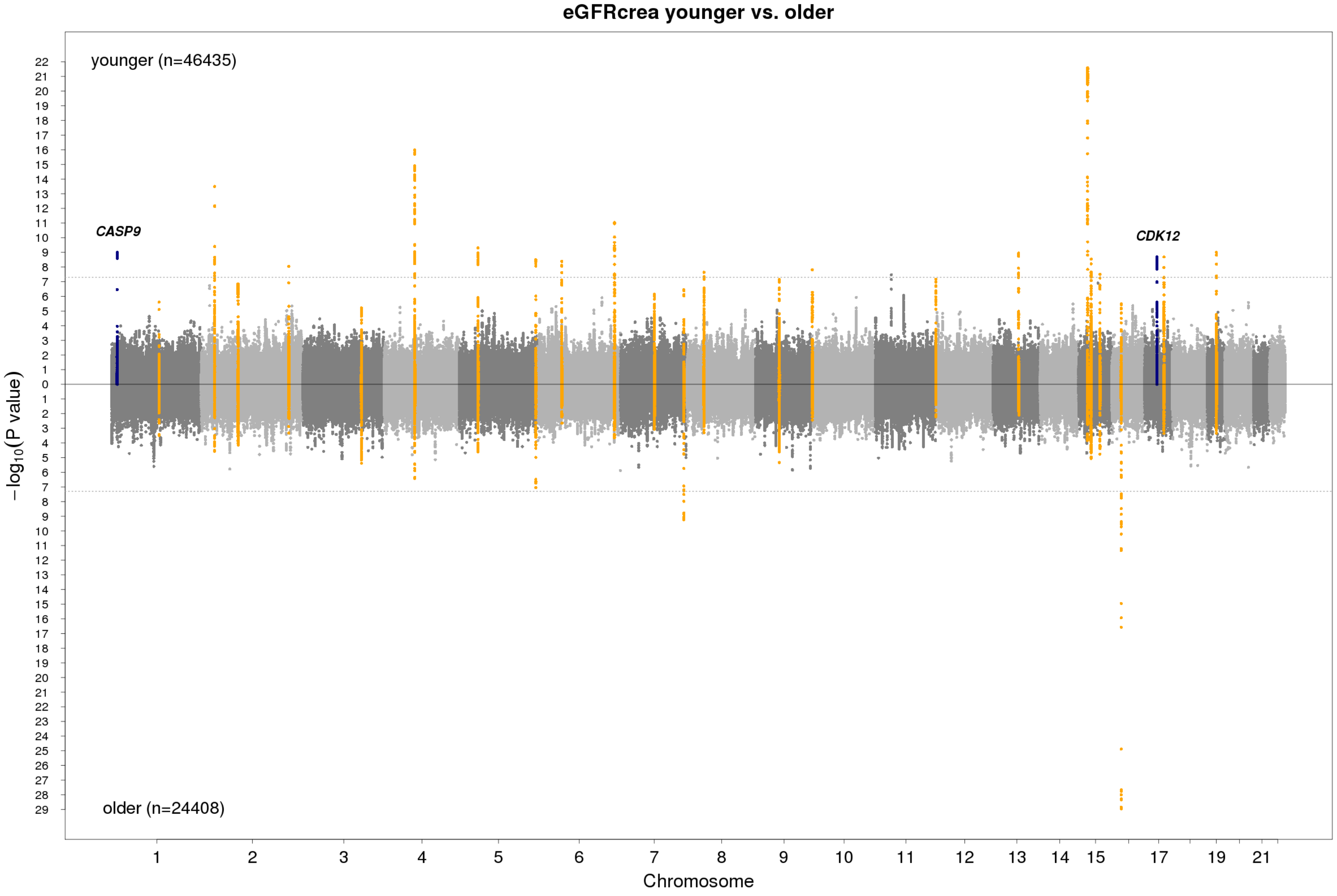

eGFRcrea younger vs. older
younger (n=46435)
older (n=24408)
CASP9
CDK12
−log10(P value)
Chromosome
22
21
20
19
18
17
16
15
14
13
12
11
10
9
8
7
6
5
4
3
2
1
0
1
2
3
4
5
6
7
8
9
10
11
12
13
14
15
16
17
18
19
20
21
22
23
24
25
26
27
28
29
1
2
3
4
5
6
7
8
9
10
11
12
13
14
15
17
19
21

E

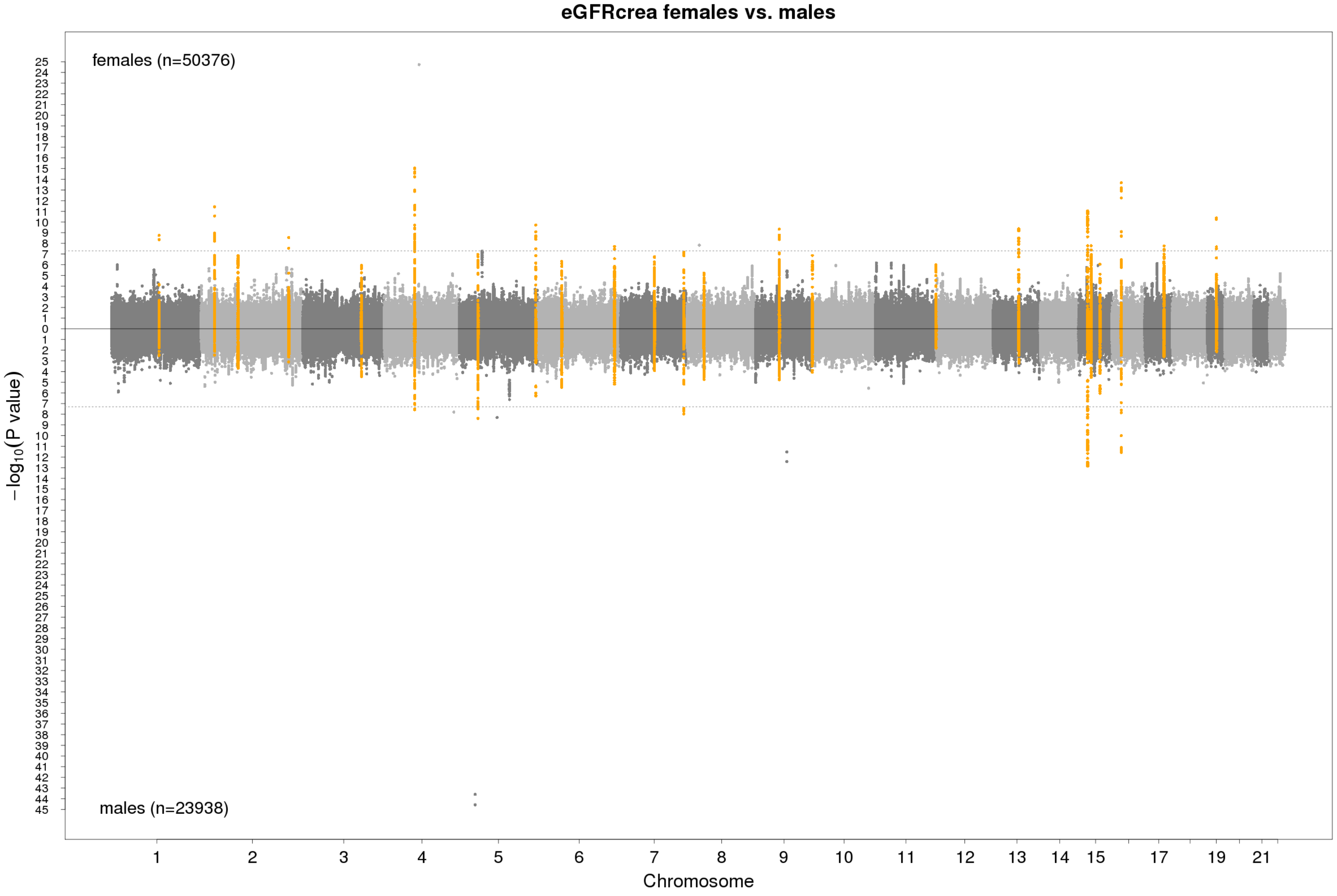

eGFRcrea females vs. males
females (n=50376)
males (n=23938)
−log10(P value)
Chromosome
1
2
3
4
5
6
7
8
9
10
11
12
13
14
15
17
19
21
