# Supplementary material for: Genome-Wide Association and Functional Follow-Up Reveals New Loci for Kidney Function
Source: PLoS Genet. 2012 Mar 29;8(3):e1002584. doi: 10.1371/journal.pgen.1002584 (PMC3315455; doi:10.1371/journal.pgen.1002584)
Supplement: Table S1 — Study-specific methods and full acknowledgments—discovery studies. (DOC) [file pgen.1002584.s013.doc]

**Table S1. Study-specific methods and full acknowledgments – discovery studies.**

| **Study name (key references)** | **Study design** | **Total genotyped sample size** | **Study exclusions or disease enrichment** | **Exclusions** | **Creatinine measurement and QC** | **Cystatin measurement and QC** | **Acknowledgments and funding source** |
| --- | --- | --- | --- | --- | --- | --- | --- |
| AGES^[1]^ | Population-based | 3,664 | None | Sample exclusion criteria included sample failure, genotype mismatch with reference panel, and sex mismatch, resulting in clean genotype data on 3,219 individuals. | Jaffé reaction (serum) | Not done | This study has been funded by NIH contract N01-AG-1-2100, the NIA Intramural Research Program, Hjartavernd (the Icelandic Heart Association), and the Althingi (the Icelandic Parliament). The study is approved by the Icelandic National Bioethics Committee, VSN: 00-063. The researchers are indebted to the participants for their willingness to participate in the study |
| Amish studies^[2, 3]^ | Population-based founder cohort | 1,264 | None | Age<20, severe chronic disease, call rate<95%, pHWE<10E-6 | Modified kinetic Jaffé reaction | Particle-enhanced immuno-nephelometric method (BNII, Dade-Behring) | We thank our Amish research volunteers for their long-standing partnership in research, and the research staff at the Amish Research Clinic for their hard work and dedication. We are supported by grants and contracts from the NIH including R01 AG18728 (Amish Longevity Study), R01 HL088119 (Amish Calcification Study), U01 GM074518-04 (PAPI Study), U01 HL072515-06 (HAPI Study), U01 HL084756 and NIH K12RR023250 (University of Maryland MCRDP), the University of Maryland General Clinical Research Center, grant M01 RR 16500, the Baltimore Veterans Administration Medical Center Geriatrics Research and Education Clinical Center and the Paul Beeson Physician Faculty Scholars in Aging Program. |
| ASPS^[4, 5]^ | Prospective, single center study | 922 | History of neuropsychiatric disease, previous stroke and/or TIA, and dementia | Of the 922 participants who underwent genotyping, 74 with sample call rate < 98% were excluded resulting in a total of 848 genotyped individuals | Modified kinetic Jaffe reaction | Not done | The research reported in this article was funded by the Austrian Science Fond (FWF) grant number P20545-P05 and P13180. The Medical University of Graz supports the databank of the ASPS. The authors thank the staff and the participants of the ASPS for their valuable contributions. We thank Birgit Reinhart for her long-term administrative commitment and Ing Johann Semmler for the technical assistance at creating the DNA-bank. |
| Atherosclerosis Risk in Communities (ARIC) Study^[6]^ | Population-based | 9,713 of European ancestry | None | Of the 9713 genotyped individuals of European ancestry, we excluded 658 individuals based on discrepancies with previous genotypes, disagreement between reported and genotypic sex, one randomly selected member of a pair of first-degree relatives, or outlier based on measures of average DST or more than 8 SD away on any of the first 10 principal components. | Modified kinetic Jaffe reaction (serum) | Particle enhanced immuno-nephelometric assay (N Latex Cystatin C, Dade Behring) (serum) | The Atherosclerosis Risk in Communities Study is carried out as a collaborative study supported by National Heart, Lung, and Blood Institute contracts (HHSN268201100005C, HHSN268201100006C, HHSN268201100007C, HHSN268201100008C, HHSN268201100009C, HHSN268201100010C, HHSN268201100011C, and HHSN268201100012C), R01HL087641, R01HL59367 and R01HL086694; National Human Genome Research Institute contract U01HG004402; and National Institutes of Health contract HHSN268200625226C. The authors thank the staff and participants of the ARIC study for their important contributions. Infrastructure was partly supported by Grant Number UL1RR025005, a component of the National Institutes of Health and NIH Roadmap for Medical Research. A.K. and C.H. were supported by the grant KO3598/2-1 (Emmy Noether Programme) of the German Research Foundation. |
| Baltimore Longitudinal Study of Aging (BLSA)^[7]^ | Population-based | 1,200 | None | Individuals of non-European descent or with missing phenotype information | Modified kinetic Jaffe reaction (serum) | Not done | The BLSA was supported in part by the Intramural Research Program of the NIH, National Institute on Aging. |
| Cardiovascular Health Study (CHS) ^[8, 9]^ | Prospective, population-based | 3,397 | In total, 1908 persons were excluded from the GWAS study sample due to the presence at study baseline of coronary heart disease, congestive heart failure, periphe-ral vascular disease, valvular heart disease, stroke or tran-sient ischemic attack or lack of available DNA | This study is based upon genotyping results from 3,329 CHS Caucasian participants, who were free of clinical cardiovascular disease at baseline, consented to genetic testing, and had DNA available for geno-typing. Genotypes were called using the Illumina BeadStudio software. Genotyping was successful in 3,291 persons. | Colorimetric method (Ektachem 700, Eastman Kodak) (serum) | Particle-enhanced immuno-nephelometric assay [N Latex Cystatin C, Dade Behring(now Siemens), Deerfield, Ill, USA] with a nephelometer [BNII, Dade Behring (now Siemens)] | The CHS research reported in this article was supported by contract numbers N01-HC-85079 through N01-HC-85086, N01-HC-35129, N01 HC-15103, N01 HC-55222, N01-HC-75150, N01-HC-45133, grant numbers U01 HL080295 and R01 HL087652 from the National Heart, Lung, and Blood Institute, with additional contribution from the National Institute of Neurological Disorders and Stroke. A full list of principal CHS investigators and institutions can be found at http://www.chs-nhlbi.org/pi.htm. DNA handling and genotyping was supported in part by National Center for Research Resources grant M01RR00425 to the Cedars-Sinai General Clinical Research Center Genotyping core and National Institute of Diabetes and Digestive and Kidney Diseases grant DK063491 to the Southern California Diabetes Endocrinology Research Center. |
| Erasmus Rucphen Family (ERF) Study^[10]^ | Population-based family study | 2,385 | None | None | Jaffe rate method using a Synchron LX20 | Not done | The ERF study was supported by grants from the Netherlands Organization for Scientific Research (NWO; Pioneergrant), Erasmus Medical Center, the Centre for Medical Systems Biology (CMSB), and the Netherlands Kidney Foundation. We are grateful to all patients and their relatives, general practitioners and neurologists for their contributions and to P. Veraart for her help in genealogy, Jeannette Vergeer for the supervision of the laboratory work and P. Snijders for his help in data collection. |
| Family Heart Study (FamHS)^[11]^ | Family-based. However the subjects contributed to this ana-lysis were largely un-related and drawn from the tails of the CAC distribution. | 974 | Cases and controls based on the upper and lower quartile of CAC, respectively. | Of the 974 participants who underwent genotyping, 883 subjects had creatinine measurement. | Thin film adaptation of the amidohydrolase enzymatic method using the Vitros analyzer (Johnson & Johnson Clinical Diagnostics, Inc. Rochester NY 14650) (serum) | Not done | The Family Heart Study (FHS) work was supported in part by NIH grants 5R01HL08770003, 5R01HL08821502 (Michael A. Province) from the NHLBI and 5R01DK07568102, 5R01DK06833603 from the NIDDK (I.B.B.). The authors thank the staff and participants of the FamHS for their important contributions. |
| Framingham Heart Study (FHS) ^[12-14]^ | Prospective, family-based | 9,300 | None | Of the 9,274 participants who underwent genotyping, we made the following exclusions: sample call rate <97% (n=666), genotype heterozygosity > 5 standard deviations, and ambiguous family data (n=127). This resulted in a total of 8,481 genotyped individuals. | Modified kinetic Jaffe reaction (serum) | Particle-enhanced immuno-nephelometric method (BNII, Dade-Behring). | This research was conducted in part using data and resources from the Framingham Heart Study of the National Heart Lung and Blood Institute of the National Institutes of Health and Boston University School of Medicine. The analyses reflect intellectual input and resource development from the Framingham Heart Study investigators participating in the SNP Health Association Resource (SHARe) project. This work was partially supported by the National Heart, Lung and Blood Institute's Framingham Heart Study (Contract No. N01-HC-25195) and its contract with Affymetrix, Inc for genotyping services (Contract No. N02-HL-6-4278). A portion of this research utilized the Linux Cluster for Genetic Analysis (LinGA-II) funded by the Robert Dawson Evans Endowment of the Department of Medicine at Boston University School of Medicine and Boston Medical Center. |
| GENOA^[15-17]^ | Family-based | 1,553 | 5 Non-white, 1 Missing Exam | For the Affymetrix 6.0 data, we excluded: 25 subjects who failed pre-processing, 123 with Contrast QC < 0.4, 2 for inconsistent relatedness, 11 identical twins. We re-ran the samples that failed pre-processing or the Contrast QC filter on the Affymetrix 6.0 data along with 50 that had passed. Of these samples, 19 failed genotyping completely, 9 had call rate < 0.95, 2 had inconsistent relatedness, and 2 were identical twins. Of these 1509 remaining samples, 346 samples do not have serum creati-nine information. Our final data had 1163 samples with geno-type and phenotype data. | Compensated rate Jaffe reaction (serum) | The Cystatin C phenotype was not available in the phase 2 GENOA lab data. It was run in phase 3, but that data was not available at the time of the CKDGen discovery analysis. | This research was partially supported by the National Heart Lung and Blood Institute of the National Institutes of Health R01 HL-87660. |
| Health ABC | Prospective cohort study | 1,663 | None | Samples were excluded from the dataset for the reasons of sample failure, genotypic sex mismatch, and first-degree relative of an included individual based on genotype data. | Colorimetric technique on a Johnson & Johnson VITROS 950 Chemistry Ana-lyzer (Johnson & Johnson, New Brunswick, NJ, USA) using the enzymatic method. | BNII nephelometer (Dade Behring Inc., Deerfield, Ill., USA) that utilized a particle-enhanced immuno-nephelometric assay (N Latex Cystatin C) | The Health Aging and Body Composition Study (Health ABC) was funded by the National Institutes of Aging. This research was supported by NIA contracts N01AG62101, N01AG62103, and N01AG62106. The genome-wide association study was funded by NIA grant 1R01AG032098-01A1 to Wake Forest University Health Sciences and genotyping services were provided by the Center for Inherited Disease Research (CIDR). CIDR is fully funded through a federal contract from the National Institutes of Health to The Johns Hopkins University, contract number HHSN268200782096C. This research was supported in part by the Intramural Research Program of the NIH, National Institute on Aging. |
| Health Professionals Follow-Up Study (HPFS)^[18]^ | Nested case-control study of type 2 diabetes | 2,487 | Type 2 diabetes | Of the 2487 subjects with genome-wide scans, 818 (all T2D cases) had creatinine measured | Modified kinetic Jaffe reaction (plasma). Creatinine values were not normalized to the Cleveland Clinic standard. Hypertension was defined as having ever reported high blood pressure or use of anti-hypertensive medication on questionnaires administered up to and including the year in which creatinine was measured (1994 for HPFS). | Not done | The NHS/HPFS type 2 diabetes GWAS (U01HG004399) is a component of a collaborative project that includes 13 other GWAS (U01HG004738, U01HG004422, U01HG004402, U01HG004729, U01HG004726, U01HG004735, U01HG004415, U01HG004436, U01HG004423, U01HG004728, RFAHG006033; National Institute of Dental & Craniofacial Research: U01DE018993, U01DE018903) funded as part of the Gene Environment-Association Studies (GENEVA) under the NIH Genes, Environment and Health Initiative (GEI). Assistance with phenotype harmonization and genotype cleaning, as well as with general study coordination, was provided by the GENEVA Coordinating Center (U01HG004446). Assistance with data cleaning was provided by the National Center for Biotechnology Information. Genotyping was performed at the Broad Institute of MIT and Harvard, with funding support from the NIH GEI (U01HG04424), and Johns Hopkins University Center for Inherited Disease Research, with support from the NIH GEI (U01HG004438) and the NIH contract "High throughput genotyping for studying the genetic contributions to human disease”(HHSN268200782096C). Additional funding for the current research was provided by the National Cancer Institute (P01CA087969, P01CA055075), and the National Institute of Diabetes and Digestive and Kidney Diseases (R01DK058845). We thank the staff and participants of the NHS and HPFS for their dedication and commitment. |
| KORA F3 and KORA F4 ^[19, 20]^ | Prospective population-based | F3 (GWAS): 1644; F4 (GWAS): 1814; F3 (de novo): 1498; F4 (de novo): 1202 | None | None | Modified kinetic Jaffe reaction (serum) | Particle-enhanced immunonephelometric method (BNII, Dade-Behring). | The genetic epidemiological work was funded by the NIH subcontract from the Children’s Hospital, Boston, US, (H.E.W., I.M.H, prime grant 1 R01 DK075787-01A1), the German National Genome Research Net NGFN2 and NGFNplus (H.E.W. 01GS0823; WK project A3, number 01GS0834), the Munich Center of Health Sciences (MC Health) as part of LMUinnovativ, and by the Else Kröner-Fresenius-Stiftung (P48/08//A11/08; C.A.B., B.K.K.). The kidney parameter measurements in F3 were funded by the Else Kröner-Fresenius-Stiftung (C.A.B., B.K.K.) and the Regensburg University Medical Center, Germany; in F4 by the University of Ulm, Germany (W.K.). Genome wide genotyping costs in F3 and F4 were in part funded by the Else Kröner-Fresenius-Stiftung (C.A.B., B.K.K.). De novo genotyping in F3 and F4 were funded by the Else Kröner-Fresenius-Stiftung (C.A.B., B.K.K.). The KORA research platform and the MONICA Augsburg studies were initiated and financed by the Helmholtz Zentrum München, German Research Center for Environmental Health, by the German Federal Ministry of Education and Research and by the State of Bavaria. Geno-typing was performed in the Genome Analysis Center (GAC) of the Helmholtz Zentrum München. The LINUX platform for computation was funded by the University of Regensburg for the Department of Epidemiology and Preventive Medicine at the Regensburg University Medical Center. |
| Korcula ^[21]^ | Cross-sectional, family-based. | NA | None | Subjects were excluded if they fulfilled any of the following criteria: genotypic call rate <97%, mismatch between reported and genotypic sex, un-expectedly low geno-mic sharing with first degree relatives, excess autosomal heterozygosity, outliers identified by IBS clustering analysis, pregnant women. | Jaffé rate method (plasma) | Not done | The Korcula study in the Croatian island of Vis was supported through the grants from the Medical Research Council UK to H.C., A.F.W. and I.R.; and Ministry of Science, Education and Sport of the Republic of Croatia to I.R. (number 108-1080315-0302). We would like to acknowledge the invaluable contributions of the recruitment team in Korcula, the administrative teams in Croatia and Edinburgh (Rosa Bisset) and the people of Korcula. |
| Microisolates in South Tyrol study (MICROS)^[22, 23]^ | Cross-sectional, population-based study using extended pedigrees | 1,391 | None | Samples with overall SNP call rate<95%, showing excess of heterozygosity, or being classified as outliers by IBS clustering analysis were excluded prior to further analyses. | Enzymatic photometric assay using an ADVIA1650 clinical chemistry analyzer (Siemens Healthcare Diagnostics GmbH, Eschborn, Germany) (serum). | BN-ProSpec analyzer (Dade Behring, Marburg, Germany) at the Institute for Clinical Chemistry and Laboratory Medicine, Regensburg University Me-dical Center, Germany. | We owe a debt of gratitude to all participants. We thank the primary care practitioners Raffaela Stocker, Stefan Waldner, Toni Pizzecco, Josef Plangger, Ugo Marcadent and the personnel of the Hospital of Silandro (Department of Laboratory Medicine) for their participation and collaboration in the research project. We thank Dr. Peter Riegler (Hemodialysis Unit, Hospital of Merano) for the important discussions. In South Tyrol, the study was supported by the Ministry of Health and Department of Educational Assistance, University and Research of the Autonomous Province of Bolzano, the South Tyrolean Sparkasse Foundation, and the European Union framework program 6 EUROSPAN project (contract no. LSHG-CT-2006-018947). |
| Northern Sweden Population Health Study (NSPHS)^[24, 25]^ | Cross-sectional, family-based | NA | None | Subjects were excluded if they fulfilled any of the following criteria: genotypic call rate <97%, mismatch between reported and genotypic sex, un-expectedly low genomic sharing with 1st degree relatives, excess autosomal heterozygosity, or outliers identified by IBS clustering analysis. | Enzymatic photometric assay using an ADVIA1650 clinical chemistry analyzer (Siemens Healthcare Diagnostics GmbH, Eschborn, Germany) (plasma). | Not done | The Northern Swedish Population Health Study was supported by grants from the Swedish Natural Sciences Research Council, the European Union through the EUROSPAN project (contract no. LSHG-CT-2006-018947), the Foundation for Strategic Research (SSF) and the Linneaus Centre for Bioinformatics (LCB). We are also grateful for the contribution of samples from the Medical Biobank in Umeå and for the contribution of the district nurse Svea Hennix in the Karesuando study. |
| Nurses' Health Study (NHS)^[26]^ | Nested case-control study of type 2 diabetes | 3,286 | Type 2 diabetes | Of the 3286 subjects with genome-wide scans, 784 (all T2D cases) had creatinine measured. | Modified kinetic Jaffe reaction. Creatinine values were not normalized to the Cleveland Clinic standard. Hypertension was defined as having ever reported high blood pressure or use of anti-hypertensive medication on questionnaires administered up to and including the year in which creatinine was measured (1990 for NHS). (plasma) | Not done | The NHS/HPFS type 2 diabetes GWAS (U01HG004399) is a component of a collaborative project that includes 13 other GWAS (U01HG004738, U01HG004422, U01HG004402, U01HG004729, U01HG004726, U01HG004735, U01HG004415, U01HG004436, U01HG004423, U01HG004728, RFAHG006033; National Institute of Dental & Craniofacial Research: U01DE018993, U01DE018903) funded as part of the Gene Environment-Association Studies (GENEVA) under the NIH Genes, Environment and Health Initiative (GEI). Assistance with phenotype harmonization and genotype cleaning, as well as with general study coordination, was provided by the GENEVA Coordinating Center (U01HG004446). Assistance with data cleaning was provided by the National Center for Biotechnology Information. Genotyping was performed at the Broad Institute of MIT and Harvard, with funding support from the NIH GEI (U01HG04424), and Johns Hopkins University Center for Inherited Disease Research, with support from the NIH GEI (U01HG004438) and the NIH contract "High throughput genotyping for studying the genetic contributions to human disease”(HHSN268200782096C). The NHS renal function and albuminuria work was supported by DK66574. Additional funding for the current research was provided by the National Cancer Institute (P01CA087969, P01CA055075), and the National Institute of Diabetes and Digestive and Kidney Diseases (R01DK058845). We thank the staff and participants of the NHS and HPFS for their dedication and commitment. |
| Orkney Complex Disease Study (ORCADES) ^[27]^ | Cross-sectional, family-based | 704 | None | Subjects were excluded if they fulfilled any of the following criteria: genotypic call rate <97%, mismatch between reported and genotypic sex, unexpectedly low genomic sharing with first degree relatives, excess autosomal heterozygosity, or outliers identified by IBS clustering analysis. | Enzymatic pho-tometric assay using an ADVIA 1650 clinical chemistry ana-lyzer (Siemens Healthcare Dia-gnostics GmbH, Eschborn, Ger-many)^[28]^ at the Institute for Clinical Chemi-stry and Labo-ratory Medicine, Regensburg University Medi-cal Center, Ger-many. (plasma) | Not done | ORCADES was supported by the Chief Scientist Office of the Scottish Government , the Royal Society and the European Union framework program 6 EUROSPAN project (contract no. LSHG-CT-2006-018947). DNA extractions were performed at the Wellcome Trust Clinical Research Facility in Edinburgh. We would like to acknowledge the invaluable contributions of Lorraine Anderson, the research nurses in Orkney, the administrative team in Edinburgh and the people of Orkney. |
| popgen^[29]^ | Prospective, population-based. | 1317 | None | Samples with >5% missing genotypes, showing excess genetic dissimilarity to the remaining subjects, or with evidence for a cryptic relatedness to other participants were removed. These quality control measures left 1241 samples for inclusion in the study. Of them, 1163 had serum creatinine available. All sex assignments could be verified by reference to the proportion of hetero-zygous SNPs on the X chromosome. | Enzymatic in vitro assay (CREAplus, Cobas®, Roche Diagnostics, Indianapolis, IN) | Not done | The popgen study was supported by the German Ministry of Education and Research (BMBF) through the National Genome Research Network (NGFN) and the Ministry of Science, Commerce and Transportation of the State of Schleswig-Holstein. The project has also received infrastructure support through the DFG excellence cluster “Inflammation at Interfaces”. |
| Sorbs^[30-32]^ | Population-based | 1,097 | None | Sample call rate<94%; Ethnic outliers; duplicates; gender mismatch; IBS>0.2 | kinetic enzymatic method (serum) | Not done | The study was funded by grants from the German Research Council KFO-152 (to Michael Stumvoll) and the IFB (Integrated Research and Treatment Center) AdiposityDiseases (K7-37 to Michael Stumvoll and Anke Tönjes). We also thank Dr. Knut Krohn (Microarray Core Facility of the Interdisciplinary Centre for Clinical Research, University of Leipzig, Germany) for providing the genotyping platform. The research of Inga Prokopenko is funded in part through the European Community's Seventh Framework Programme (FP7/2007-2013), ENGAGE project, grant agreement HEALTH-F4-2007- 201413. Reedik Magi acknowledges financial support from the European Commission under a Marie Curie Intra-European Fellowship. |
| The Rotterdam Study-I and Rotterdam Study-II^[33-35]^ | Prospective, population-based. | RS-I: 5,974; RS-II: 1,895 | None | Any samples with a call rate<97.5%, excess autosomal heterozygosity >0.336 (~FDR <0.1%), mismatch between called and phenotypic gender, or if there were outliers identified by the IBS clustering analysis (see below) with >3 standard deviations from population mean or IBS probabilities >97% were excluded from the analysis. | Modified kinetic Jaffe reaction (serum) | Not done | The GWA study was funded by the Netherlands Organisation of Scientific Research NWO Investments (nr. 175.010.2005.011, 911-03-012), the Research Institute for Diseases in the Elderly (014-93-015; RIDE2), the Netherlands Genomics Initiative (NGI)/Netherlands Consortium for Healthy Aging (NCHA) project nr. 050-060-810. We thank Pascal Arp, Mila Jhamai, Dr Michael Moorhouse, Marijn Verkerk, and Sander Bervoets for their help in creating the GWAS database. The Rotterdam Study is funded by Erasmus Medical Center and Erasmus University, Rotterdam, Netherlands Organization for the Health Research and Development (ZonMw), the Research Institute for Diseases in the Elderly (RIDE), the Ministry of Education, Culture and Science, the Ministry for Health, Welfare and Sports, the European Commission (DG XII), and the Municipality of Rotterdam. The authors are very grateful to the participants and staff from the Rotterdam Study, the participating general practitioners and the pharmacists. We would like to thank Dr. Tobias A. Knoch, Luc V. de Zeeuw, Anis Abuseiris, and Rob de Graaf as well as their institutions the Erasmus Computing Grid, Rotterdam, The Netherlands, and especially the national German MediGRID and Services@MediGRID part of the German D-Grid, both funded by the German Bundes-ministerium für Forschung und Technology under grants #01 AK 803 A-H and #01 IG 07015 G, for access to their grid resources. Abbas Dehghan is supported by NWO grant (vici, 918-76-619). |
| The Study of Health in Pomerania (SHIP)^[36, 37]^ | Prospective population-based | 4,105 | None | The following samples were excluded: Affymetrix QC call rate <86%, sample call rate <92%, duplicate samples (by IBD estimation), individuals with reported/genotyped gender mismatch. | A blood sample was drawn from the cubital vein in the supine position (parti-cipants were non-fasting due to the duration of the cumu-lative examina-tions, 4-6 hours in total). Serum creatinine levels were determined with the Jaffé method. | Siemens N Latex Cystatin C assay, a particle-enhanced nephelometric immunoassay, on the BN ProSpec® System (serum). | SHIP is part of the Community Medicine Research net of the University of Greifswald, Germany, which is funded by the Federal Ministry of Education and Research (grants no. 01ZZ9603, 01ZZ0103, and 01ZZ0403), the Ministry of Cultural Affairs as well as the Social Ministry of the Federal State of Mecklenburg-West Pomerania. Genome-wide data have been supported by the Federal Ministry of Education and Research (grant no. 03ZIK012) and a joint grant from Siemens Healthcare, Erlangen, Germany and the Federal State of Mecklenburg- West Pomerania. The University of Greifswald is a member of the ‘Center of Knowledge Interchange’ program of the Siemens AG. |
| Vis^[38, 39]^ | Cross-sectional, family-based | NA | None | Subjects were excluded if they fulfilled any of the following criteria: genotypic call rate <97%, mismatch between reported and genotypic sex, unexpectedly low genomic sharing with first degree relatives, excess autosomal heterozygosity, or outliers identified by IBS clustering analysis. Pregnant women were excluded. | Enzymatic photometric assay using an ADVIA1650 clinical chemi-stry analyzer (Siemens Healthcare Dia-gnostics GmbH, Eschborn, Ger-many)^[28]^ at the Institute for Cli-nical Chemistry and Laboratory Medicine, Re-gensburg Uni-versity Medical Center, Ger-many (serum). | Not done | The Vis study in the Croatian island of Vis was supported through the grants from the Medical Research Council UK to H.C., A.F.W. and I.R.; and Ministry of Science, Education and Sport of the Republic of Croatia to I.R. (number 108-1080315-0302) and the European Union framework program 6 EUROSPAN project (contract no. LSHG-CT-2006-018947). We would like to acknowledge the invaluable contributions of the recruitment team (including those from the Institute of Anthropological Research in Zagreb) in Vis, the administrative teams in Croatia and Edinburgh (Rosa Bisset) and the people of Vis. |
| Women’s Genome Health Study (WGHS)^[40]^ | Prospective, population-based | 21,940 | Non-European ancestry was an exclusion criterion | Samples with <98% successful SNP calls were excluded. Data had been collected on 21940 individuals had successful genotype information and verified European ancestry at the time of the analysis. | Rate-blanked method based on the Jaffé reaction using Roche Diagno-stics rea-gents with reproducibi-lity of 3.67% and 1.60% at con-centrations of 1.17 and 6.40 mg/dL, respectively. | Not done | The WGHS is supported by HL 043851 and HL69757 from the National Heart, Lung, and Blood Institute and CA 047988 from the National Cancer Institute, the Donald W. Reynolds Foundation and the Fondation Leducq, with collaborative scientific support and funding for genotyping provided by Amgen. |

References

1.     Harris TB, Launer LJ, Eiriksdottir G, Kjartansson O, Jonsson PV, et al. (2007) Age, Gene/Environment susceptibility-reykjavik study: Multidisciplinary applied phenomics. Am J Epidemiol 165(9): 1076-1087.

2.     Mitchell BD, McArdle PF, Shen H, Rampersaud E, Pollin TI, et al. (2008) The genetic response to short-term interventions affecting cardiovascular function: Rationale and design of the heredity and phenotype intervention (HAPI) heart study. Am Heart J 155(5): 823-828.

3.     Rampersaud E, Bielak LF, Parsa A, Shen H, Post W, et al. (2008) The association of coronary artery calcification and carotid artery intima-media thickness with distinct, traditional coronary artery disease risk factors in asymptomatic adults. Am J Epidemiol 168(9): 1016-1023.

4.     Schmidt R, Lechner H, Fazekas F, Niederkorn K, Reinhart B, et al. (1994) Assessment of cerebrovascular risk profiles in healthy persons: Definition of research goals and the austrian stroke prevention study (ASPS). Neuroepidemiology 13(6): 308-313.

5.     Schmidt R, Fazekas F, Kapeller P, Schmidt H, Hartung HP. (1999) MRI white matter hyperintensities: Three-year follow-up of the austrian stroke prevention study. Neurology 53(1): 132-139.

6.     The ARIC investigators. (1989) The atherosclerosis risk in communities (ARIC) study: Design and objectives. Am J Epidemiol 129(4): 687-702.

7.     Shock NW, et al. (1984) Normal human aging: The baltimore longitudinal study of aging. Superintendent of Documents, U.S. Government Printing Office, Washington, DC 20402. .

8.     Fried LP, Borhani NO, Enright P, Furberg CD, Gardin JM, et al. (1991) The cardiovascular health study: Design and rationale. Ann Epidemiol 1(3): 263-276.

9.     Heard-Costa NL, Zillikens MC, Monda KL, Johansson A, Harris TB, et al. (2009) NRXN3 is a novel locus for waist circumference: A genome-wide association study from the CHARGE consortium. PLoS Genet 5(6): e1000539.

10.     Aulchenko YS, Heutink P, Mackay I, Bertoli-Avella AM, Pullen J, et al. (2004) Linkage disequilibrium in young genetically isolated dutch population. Eur J Hum Genet 12(7): 527-534.

11.     Higgins M, Province M, Heiss G, Eckfeldt J, Ellison RC, et al. (1996) NHLBI family heart study: Objectives and design. Am J Epidemiol 143(12): 1219-1228.

12.     Feinleib M, Kannel WB, Garrison RJ, McNamara PM, Castelli WP. (1975) The framingham offspring study. design and preliminary data. Prev Med 4(4): 518-525.

13.     Kannel WB, Feinleib M, McNamara PM, Garrison RJ, Castelli WP. (1979) An investigation of coronary heart disease in families. the framingham offspring study. Am J Epidemiol 110(3): 281-290.

14.     Splansky GL, Corey D, Yang Q, Atwood LD, Cupples LA, et al. (2007) The third generation cohort of the national heart, lung, and blood institute's framingham heart study: Design, recruitment, and initial examination. Am J Epidemiol 165(11): 1328-1335.

15.     Turner ST, Kardia SL, Mosley TH, Rule AD, Boerwinkle E, et al. (2006) Influence of genomic loci on measures of chronic kidney disease in hypertensive sibships. J Am Soc Nephrol 17(7): 2048-2055.

16.     Rule AD, Jacobsen SJ, Schwartz GL, Mosley TH, Scott CG, et al. (2006) A comparison of serum creatinine-based methods for identifying chronic kidney disease in hypertensive individuals and their siblings. Am J Hypertens 19(6): 608-614.

17.     Daniels PR, Kardia SL, Hanis CL, Brown CA, Hutchinson R, et al. (2004) Familial aggregation of hypertension treatment and control in the genetic epidemiology network of arteriopathy (GENOA) study. Am J Med 116(10): 676-681.

18.     Rimm EB, Giovannucci EL, Willett WC, Colditz GA, Ascherio A, et al. (1991) Prospective study of alcohol consumption and risk of coronary disease in men. Lancet 338(8765): 464-468.

19.     Baumeister SE, Boger CA, Kramer BK, Doring A, Eheberg D, et al. (2010) Effect of chronic kidney disease and comorbid conditions on health care costs: A 10-year observational study in a general population. Am J Nephrol 31(3): 222-229.

20.     Wichmann HE, Gieger C, Illig T, MONICA/KORA Study Group. (2005) KORA-gen--resource for population genetics, controls and a broad spectrum of disease phenotypes. Gesundheitswesen 67 Suppl 1: S26-30.

21.     Polasek O, Marusic A, Rotim K, Hayward C, Vitart V, et al. (2009) Genome-wide association study of anthropometric traits in korcula island, croatia. Croat Med J 50(1): 7-16.

22.     Pattaro C, Marroni F, Riegler A, Mascalzoni D, Pichler I, et al. (2007) The genetic study of three population microisolates in south tyrol (MICROS): Study design and epidemiological perspectives. BMC Med Genet 8: 29.

23.     Marroni F, Grazio D, Pattaro C, Devoto M, Pramstaller P. (2008) Estimates of genetic and environmental contribution to 43 quantitative traits support sharing of a homogeneous environment in an isolated population from south tyrol, italy. Hum Hered 65(3): 175-182.

24.     Johansson A, Vavruch-Nilsson V, Cox DR, Frazer KA, Gyllensten U. (2007) Evaluation of the SNP tagging approach in an independent population sample--array-based SNP discovery in sami. Hum Genet 122(2): 141-150.

25.     Johansson A, Vavruch-Nilsson V, Edin-Liljegren A, Sjolander P, Gyllensten U. (2005) Linkage disequilibrium between microsatellite markers in the swedish sami relative to a worldwide selection of populations. Hum Genet 116(1-2): 105-113.

26.     Colditz GA, Hankinson SE. (2005) The nurses' health study: Lifestyle and health among women. Nat Rev Cancer 5(5): 388-396.

27.     McQuillan R, Leutenegger AL, Abdel-Rahman R, Franklin CS, Pericic M, et al. (2008) Runs of homozygosity in european populations. Am J Hum Genet 83(3): 359-372.

28.     Guder WG, Hoffmann GE, Hubbuch A, Poppe WA, Siedel J, et al. (1986) Multicentre evaluation of an enzymatic method for creatinine determination using a sensitive colour reagent. J Clin Chem Clin Biochem 24(11): 889-902.

29.     Krawczak M, Nikolaus S, von Eberstein H, Croucher PJ, El Mokhtari NE, et al. (2006) PopGen: Population-based recruitment of patients and controls for the analysis of complex genotype-phenotype relationships. Community Genet 9(1): 55-61.

30.     Tonjes A, Zeggini E, Kovacs P, Bottcher Y, Schleinitz D, et al. (2010) Association of FTO variants with BMI and fat mass in the self-contained population of sorbs in germany. Eur J Hum Genet 18(1): 104-110.

31.     Veeramah KR, Tonjes A, Kovacs P, Gross A, Wegmann D, et al. (2011) Genetic variation in the sorbs of eastern germany in the context of broader european genetic diversity. Eur J Hum Genet 19(9): 995-1001.

32.     Tonjes A, Koriath M, Schleinitz D, Dietrich K, Bottcher Y, et al. (2009) Genetic variation in GPR133 is associated with height: Genome wide association study in the self-contained population of sorbs. Hum Mol Genet 18(23): 4662-4668.

33.     Hofman A, Grobbee DE, de Jong PT, van den Ouweland FA. (1991) Determinants of disease and disability in the elderly: The rotterdam elderly study. Eur J Epidemiol 7(4): 403-422.

34.     Hofman A, Breteler MM, van Duijn CM, Krestin GP, Pols HA, et al. (2007) The rotterdam study: Objectives and design update. Eur J Epidemiol 22(11): 819-829.

35.     Hofman A, Breteler MM, van Duijn CM, Janssen HL, Krestin GP, et al. (2009) The rotterdam study: 2010 objectives and design update. Eur J Epidemiol 24(9): 553-572.

36.     John U, Greiner B, Hensel E, Ludemann J, Piek M, et al. (2001) Study of health in pomerania (SHIP): A health examination survey in an east german region: Objectives and design. Soz Praventivmed 46(3): 186-194.

37.     Volzke H, Alte D, Schmidt CO, Radke D, Lorbeer R, et al. (2011) Cohort profile: The study of health in pomerania. Int J Epidemiol 40(2): 294-307.

38.     Rudan I, Campbell H, Rudan P. (1999) Genetic epidemiological studies of eastern adriatic island isolates, croatia: Objective and strategies. Coll Antropol 23(2): 531-546.

39.     Rudan I, Biloglav Z, Vorko-Jovic A, Kujundzic-Tiljak M, Stevanovic R, et al. (2006) Effects of inbreeding, endogamy, genetic admixture, and outbreeding on human health: A (1001 dalmatians) study. Croat Med J 47(4): 601-610.

40.     Ridker PM, Chasman DI, Zee RY, Parker A, Rose L, et al. (2008) Rationale, design, and methodology of the women's genome health study: A genome-wide association study of more than 25,000 initially healthy american women. Clin Chem 54(2): 249-255.
