# Supplementary material for: Genome-Wide Association and Functional Follow-Up Reveals New Loci for Kidney Function
Source: PLoS Genet. 2012 Mar 29;8(3):e1002584. doi: 10.1371/journal.pgen.1002584 (PMC3315455; doi:10.1371/journal.pgen.1002584)
Supplement: Table S2 — Study-specific methods and full acknowledgments—replication studies and functional follow-up studies. (DOC) [file pgen.1002584.s014.doc]

**Table S2. Study-specific methods and full acknowledgments – replication studies and functional follow-up studies.**

| **Study name (key references)** | **Study design** | **Total genotyped sample size** | **Study exclusions or disease enrichment** | **Exclusions** | **Creatinine measurement and QC** | **Cystatin measurement and QC** | **Acknowledgments and funding source** |
| --- | --- | --- | --- | --- | --- | --- | --- |
| 3 City Study^[1, 2]^ | Prospective population-based | 6,393 | None | DNA samples were transferred to the French Centre National de Génotypage (CNG) for genotyping. First stage samples that passed DNA quality control were genotyped with Illumina Human 610-Quad BeadChips. Genotype data were retained in the study for samples that had been successfully genotyped for >98% of the SNP markers. SNPs with call rate <98%, minor allele frequency<1% or exhi-biting departure from the Hardy-Weinberg equilibrium in the 3C population (p<10^-6^) were excluded. We also removed 980 samples because they were found to be 1st or 2nd-degree relatives of other study partici-pants or were assessed non-European descent based on genetic analysis. This led us to retain 5,413 individuals. | Modified kinetic Jaffe reaction | Particle-enhanced immuno-nephelometric method (BNII, Dade-Behring/ Siemens) | The work was made possible by the generous participation of the control subjects, the patients, and their families. We thank Dr. Anne Boland (CNG) for her technical help in preparing the DNA samples for analyses. This work was supported by the National Foundation for Alzheimer’s disease and related disorders, the Institut Pasteur de Lille and the Centre National de Génotypage. The Three-City Study was performed as part of a collaboration between the Institut National de la Santé et de la Recherche Médicale (Inserm), the Victor Segalen Bordeaux II University and Sanofi-Synthélabo. The Fondation pour la Recherche Médicale funded the preparation and initiation of the study. The 3C Study was also funded by the Caisse Nationale Maladie des Travailleurs Salariés, Direction Générale de la Santé, MGEN, Institut de la Longévité, Agence Française de Sécurité Sanitaire des Produits de Santé, the Aquitaine and Bourgogne Regional Councils, Fondation de France and the joint French Ministry of Research/INSERM “Cohortes et collections de données biologiques” programme. Lille Génopôle received an unconditional grant from Eisai. |
| Blue Mountains Eye Study (BMES)^[3-5]^ | Prospective cohort study | 2,761 | None | Of the 2761 partici-pants who underwent genotyping, we made the following exclu-sions: sample call rate <95% (n=9), outlying autosomal hetero-zygosity (n=28), sex discrepancies or ambi-guous sample identi-fication (n=69), cryptic relatedness (average IBD sharing proportion >0.1875: n=121), non-European ancestry (n=13). This resulted in a total of 2534 genotyped individuals. | Measured within 4 hours of collection using a Hitachi 747 Biochemistry analyzer (Roche reagents, modified kinetic Jaffe) in the serum | Not done | The Blue Mountains Eye Study has been supported by the Australian RADGAC grant (1992-94) and Australian National Health & Medical Research Council, Canberra Australia (Grant Nos: 974159, 211069, 991407, 457349). The GWAS studies of Blue Mountains Eye Study population are supported by the Australian National Health & Medical Research Council (Grant Nos: 512423, 475604, 529912) and the Wellcome Trust, UK (2008) as part of Wellcome Trust Case Control Consortium 2 (A Viswanathan, P McGuffin, P Mitchell, F Topouzis, P Foster, Grant numbers 085475/B/08/Z and 085475/08/Z). EGH and JJW are funded by the Australian National Health & Medical Research Council Fellowship Schemes. |
| CoLaus^[6]^ | Population based study | 5,636 | None | Call rate <90%, related individuals, resulting in 5435 genotyped individuals in total | Modified kinetic Jaffe reaction (maximum inter and intra-batch CVs: 2.9% – 0.7%) (Roche Diagnostics, Switzerland) in the serum | Not done | The CoLaus study received financial contributions from GlaxoSmithKline, the Faculty of Biology and Medicine of Lausanne, and the Swiss National Science Foundation (33CSCO-122661). The authors thank Gérard Waeber, Vincent Mooser and Dawn Waterworth, Co-PIs of the CoLaus study. Special thanks to Yolande Barreau, Mathieu Firmann, Vladimir Mayor, Anne-Lise Bastian, Binasa Ramic, Martine Moranville, Martine Baumer, Marcy Sagette, Jeanne Ecoffey and Sylvie Mermoud for data collection. M Bochud is supported by the Swiss School of Public Health Plus (SSPH+). |
| Cardiovascular risk in Young Finns Study (YFS)^[7]^ | Cohort study | 2,442 | None | After Sanger genotyping pipeline QC criteria (ie: duplicated samples, heterozygo-sity, low call rate, or Sequenom fingerprint discrepancy) the geno-typing pipeline con-tained 2500 subjects, after genotype filtering and relativity check (pi-hat>0.2) there were 2442 genotyped YFS subjects. Of these, 2023 with full genotype and kidney function data formed the final study sample. | Spectrophoto-metrically by the Jaffé method (picric acid; Olympus Diagnostica GmbH) from frozen plasma samples in 2007. | Not done | The Cardiovascular Risk in Young Finns study (YFS) is supported by the Academy of Finland (grant no. 117797, 121584 and 126925), the Social Insurance Institution of Finland, University Hospital Medical funds to Tampere, and Turku University Hospitals, the Finnish Foundation of Cardiovascular Research. The Emil Aaaltonen Foundation (T.L.). |
| Estonian Genome Center of University of Tartu (EGCUT)^[8, 9]^ | Longitudinal population-based | 1,000 | None | Sample call rate <95%; sex mismatch; relatedness; Sample size after cleaning 893 | Modified Jaffe protein compensated method in the serum | Not done | EGCUT received support from FP7 grants ((201413 ENGAGE, 212111 BBMRI, 205419 ECOGENE, 245536 OPENGENE). EGCUT also received targeted financing from Estonian Government SF0180142s08 and from the European Union through the European Regional Development Fund, in the frame of Centre of Excellence in Genomics. EGC authors want to acknowledge EGCUT per-sonnel, especially Ms. Merli Hass. The genotyping of the EGCUT samples were performed in Estonian Biocentre Genotyping Core Facility, EGC authors want to acknowledge Dr.Mari Nelis and Mr. Viljo Soo for their contribution in that. EGCUT data analyzes were carried out in part in the High Perfor-mance Computing Center of University of Tartu. |
| Family Heart Study-II ^[10]^ | Family-based cohort | 1,495 | None | Of the 1495 individuals genotyped success-fully, we made the following exclusions: ancestry ambiguity (n=1) and sex inconsistency (n=8). This resulted in a total of 1486 genotyped subjects, of which 1426 had both genotype and creatinine phenotype. | Creatinine iminohydrolase procedure were done in the Ektachem 400 Analyzer (Eastman Kodak Co., Rochester, NY, 14650) | Not done | This research was conducted in part using data and resources from the NHLBI Family Heart Study supported in part by NIH grant 5R01HL08770002. |
| GoDARTs^[11, 12]^ | Longitudinal Population based study | 3,028 | None | Call rate < 98% | Modified kinetic Jaffe reaction in the serum | Not done | We are grateful to all the participants who took part in this study, the general practitioners and Scottish School of Primary Care for their help in recruiting them, and the whole team, which includes interviewers, computer and laboratory technicians, clerical workers, research scientists, volunteers, managers, receptionists and nurses. The Wellcome Trust provides support for Wellcome Trust United Kingdom Type 2Diabetes Case Control Collection and the informatics support is provided by the Chief Scientist Office, and the Wellcome Trust funded Scottish Health Informatics Programme (SHIP). |
| INGI-Carlantino Project ^[13-15]^ | Isolated population | 679 | None | We obtained creatinine levels for 447 participants and excluded samples with call rate <97% | Jaffè reaction in the serum | Not done | The INGI-Carlantino and INGI-FVG studies were supported by grants from Telethon, FVG region and Fondo Trieste. |
| INGI-Cilento Study ^[15-21]^ | Population based with extended pedigree | 855 | None | Of the 855 participants who underwent genotyping, none was excluded | Kinetic Jaffe method in the serum | Not done | We thank the populations of Cilento for their participation in the study. This work was supported by grants from the EU (Vasoplus-037254), the Italian Ministry of Universities (FIRB -RBIN064YAT), the Assessorato Ricerca Regione Campania, the Ente Parco Nazionale del Cilento e Vallo di Diano and the Fondazione Banco di Napoli to MC. |
| INGI-FVG Project ^[13-15]^ | Isolated population | 1,376 | None | We obtained creatinine levels for 874 participants and excluded samples with call rate <97% | Jaffè reaction in the serum | Not done | The INGI-Carlantino and INGI-FVG studies were supported by grants from Telethon, FVG region and Fondo Trieste. |
| INGI – Val Borbera Study^[19]^ | Prospective Population based | 1,665 | None | Of the 1665 genotyped participants, we excluded 1 with sample call rate <95% | Synchron LX Beckman-Coulter using a kinetic Jaffe reaction on serum | Not done | The research was done using data obtained thanks to funds from Compagnia di San Paolo, Torino, Italy, Cariplo Fundation, Milano, Italy and Italian Mi-nistry of Health Progetto Finalizzato 2007 and 2009. |
| JUPITER ^[22]^ | Clinical trial | 12,649 | None | Successfully genotyped samples had >98.5% of SNPs genotyped. In JUPITER, the total number genotyped samples was 12,649 among which 8,782 had self-reported European ancestry that could be confirmed by the MDS procedure in PLINK. | Creatinine analyses were performed in JUPITER core central laboratories using the Roche Modular Analytics Chemistry System with Roche creatinine reagents (modified Jaffé reaction with rate blanking). Intra- and inter-assay coefficients of variation were 4.1% and 3.9%, respectively. | Not done | The JUPITER trial and the genotyping were supported by AstraZeneca. |
| Ogliastra Genetic Park (OGP) - Replication Study and OGP - Talana study^[23-25]^ | Population-based study with pedigree information | OGP-replication study: 3,000; OGP-Talana study: 860 | None | None | Modified kinetic Jaffe reaction or enzymatic reaction in the serum | Not done | We thank the Ogliastra population and all the individuals who participated in this study. We are very grateful to the municipal administrators for their collaboration to the project and for economic and logistic support. This work was supported by grants from the Italian Ministry of Education, University and Research (MIUR) no.5571/DSPAR/2002 and (FIRB) D. M. no. 718/Ric/2005. |
| PROSPER Study ^[26-28]^ | Randomized controlled clinical trial | 5,804 | Subjects were included when they had had a vascular event or were at increased risk of vascular disease, age between 70-82 | 5763 samples underwent genotyping; after QC (sample rate<97.5, genotype heterozygosity, ambiguous family data), 5,244 genotyped individuals were available for analysis | Measured at central laboratories, one in each of the three participating countries in the serum | Not done | The Prospective Study of Pravastatin in the Elderly at Risk (PROSPER) trial was supported by an investigator initiated grant from Bristol-Myers Squibb, USA. The study was conducted, analyzed, and reported independently of the company. |
| SAPALDIA cohort ^[29-33]^ | Prospective population-based | 1,444 | Enriched for self-reported asthma | Sample call rate<97%; non-Caucasian ethnicity; sex inconsistency; exclusion of SNPs with call rate<97% | Jaffé reaction (Roche) and calibrated to the Roche enzymatic gold standard reference in the serum | Not done | The SAPALDIA team: Study directorate: T Rochat (p), JM Gaspoz (c), N Künzli (e/exp), LJS Liu (exp), NM Probst Hensch (e/g), C Schindler (s). Scientific team: U Ackermann-Liebrich (e), JC Barthélémy (c), W Berger (g), R Bettschart (p), A Bircher (a), G Bolognini (p), O Brändli (p), C Brombach (n), M Brutsche (p), L Burdet (p), M Frey (p), U Frey (pd), MW Gerbase (p), D Gold (e/c/p), E de Groot (c), W Karrer (p), R Keller (p), B Knöpfli (p), B Martin (pa), D Miedinger (o), U Neu (exp), L Nicod (p), M Pons (p), F Roche (c), T Rothe (p), E Russi (p), P Schmid-Grendelmeyer (a), M Tamm (P), A Schmidt-Trucksäss (pa), A Turk (p), J Schwartz (e), D. Stolz (p), P Straehl (exp), JM Tschopp (p), A von Eckardstein (cc), JP Zellweger (p), E Zemp Stutz (e). Scientific team at coordinating centers: M Adam (e/g), E Boes (g), PO Bridevaux (p), D Carballo (c), E Corradi (e), I Curjuric (e), J Dratva (e), A Di Pasquale (s), L Grize (s), D Keidel (s), S Kriemler (pa), A Kumar (g), M Imboden (g), N Maire (s), A Mehta (e), F Meier (e), H Phuleria (exp), E Schaffner (s), GA Thun (g) A Ineichen (exp), M Ragettli (e), M Ritter (exp), T Schikowski (e), G Stern (pd), M Tarantino (s), M Tsai (e), M Wanner (pa) [(a) allergology, (c) cardiology, (cc) clinical chemistry, (e) epidemiology, (exp) exposure, (g) genetic and molecular biology, (m) meteorology, (n) nutrition, (o) occupational health, (p) pneumology, (pa) physical activity, (pd) pediatrics, (s) statistics ] Local fieldworkers: Aarau: S Brun, G Giger, M Sperisen, M Stahel, Basel: C Bürli, C Dahler, N Oertli, I Harreh, F Karrer, G Novicic, N Wyttenbacher, Davos: A Saner, P Senn, R Winzeler, Geneva: F Bonfils, B Blicharz, C Landolt, J Rochat, Lugano: S Boccia, E Gehrig, MT Mandia, G Solari, B Viscardi, Montana: AP Bieri, C Darioly, M Maire, Payerne: F Ding, P Danieli A Vonnez, Wald: D Bodmer, E Hochstrasser, R Kunz, C Meier, J Rakic, U Schafroth, A Walder. Administrative staff: C Gabriel, R Gutknecht. The Swiss National Science Foundation (grants no 33CSCO-108796, 3247BO-104283, 3247BO-104288, 3247BO-104284, 3247-065896, 3100-059302, 3200-052720, 3200-042532, 4026-028099), the Federal Office for Forest, Environment and Landscape, the Federal Office of Public Health, the Federal Office of Roads and Transport, the canton's government of Aargau, Basel-Stadt, Basel-Land, Geneva, Luzern, Ticino, Zurich, the Swiss Lung League, the canton's Lung League of Basel Stadt/ Basel Landschaft, Geneva, Ticino and Zurich. |
| Saphir ^[33, 34]^ | Healthy working population | 1,726 | None | None | Modified kinetic Jaffé reaction (CREA®, Roche Diagnostics GmbH, Mannheim, Ger-many) in the serum | Not done | The SAPHIR-study was partially supported by a grant from the Kamillo Eisner Stiftung to B. Paulweber and by grants from the "Genomics of Lipid-associated Disorders – GOLD" of the "Austrian Genome Research Programme GEN-AU" to F. Kronenberg. |
| eQTL analysis | NA | NA | NA | NA | NA | NA | We acknowledge the advice and support from Eric L. van Nostrand, Jesse Karmazin, Frederick G. Mann, Sarah J. Aerni, the Stanford PAN Facility, the Stanford Tissue Bank and the Bio-X2 cluster supercomputer (NSF award CNS-0619926). HJG received support from the AFAR/EMF postdoctoral fellowship and the Stanford Dean’s postdoctoral fellowship. HEW and SKK were supported by grants from the NIA, NHGRI and NIGMS. |

References

1.     3C Study Group. (2003) Vascular factors and risk of dementia: Design of the three-city study and baseline characteristics of the study population. Neuroepidemiology 22(6): 316-325.

2.     Lambert JC, Heath S, Even G, Campion D, Sleegers K, et al. (2009) Genome-wide association study identifies variants at CLU and CR1 associated with alzheimer's disease. Nat Genet 41(10): 1094-1099.

3.     Mitchell P, Smith W, Attebo K, Wang JJ. (1995) Prevalence of age-related maculopathy in australia. the blue mountains eye study. Ophthalmology 102(10): 1450-1460.

4.     Attebo K, Mitchell P, Smith W. (1996) Visual acuity and the causes of visual loss in australia. the blue mountains eye study. Ophthalmology 103(3): 357-364.

5.     Leeder SR, Mitchell P, Liew G, Rochtchina E, Smith W, et al. (2006) Low hemoglobin, chronic kidney disease, and risk for coronary heart disease-related death: The blue mountains eye study. J Am Soc Nephrol 17(1): 279-284.

6.     Firmann M, Mayor V, Vidal PM, Bochud M, Pecoud A, et al. (2008) The CoLaus study: A population-based study to investigate the epidemiology and genetic determinants of cardiovascular risk factors and metabolic syndrome. BMC Cardiovasc Disord 8: 6.

7.     Raitakari OT, Juonala M, Ronnemaa T, Keltikangas-Jarvinen L, Rasanen L, et al. (2008) Cohort profile: The cardiovascular risk in young finns study. Int J Epidemiol 37(6): 1220-1226.

8.     Nelis M, Esko T, Magi R, Zimprich F, Zimprich A, et al. (2009) Genetic structure of europeans: A view from the north-east. PLoS One 4(5): e5472.

9.     Metspalu A. (2004) The estonian genome project. Drug Dev Res 62: 97-101.

10.     Higgins M, Province M, Heiss G, Eckfeldt J, Ellison RC, et al. (1996) NHLBI family heart study: Objectives and design. Am J Epidemiol 143(12): 1219-1228.

11.     Morris AD, Boyle DI, MacAlpine R, Emslie-Smith A, Jung RT, et al. (1997) The diabetes audit and research in tayside scotland (DARTS) study: Electronic record linkage to create a diabetes register. DARTS/MEMO collaboration. BMJ 315(7107): 524-528.

12.     Morris AD, Boyle DI, McMahon AD, Greene SA, MacDonald TM, et al. (1997) Adherence to insulin treatment, glycaemic control, and ketoacidosis in insulin-dependent diabetes mellitus. the DARTS/MEMO collaboration. diabetes audit and research in tayside scotland. medicines monitoring unit. Lancet 350(9090): 1505-1510.

13.     D'Adamo P, Ulivi S, Beneduci A, Pontoni G, Capasso G, et al. (2010) Metabonomics and population studies: Age-related amino acids excretion and inferring networks through the study of urine samples in two italian isolated populations. Amino Acids 38(1): 65-73.

14.     Tepper BJ, Koelliker Y, Zhao L, Ullrich NV, Lanzara C, et al. (2008) Variation in the bitter-taste receptor gene TAS2R38, and adiposity in a genetically isolated population in southern italy. Obesity (Silver Spring) 16(10): 2289-2295.

15.     Sala C, Ciullo M, Lanzara C, Nutile T, Bione S, et al. (2008) Variation of hemoglobin levels in normal italian populations from genetic isolates. Haematologica 93(9): 1372-1375.

16.     Ciullo M, Bellenguez C, Colonna V, Nutile T, Calabria A, et al. (2006) New susceptibility locus for hypertension on chromosome 8q by efficient pedigree-breaking in an italian isolate. Hum Mol Genet 15(10): 1735-1743.

17.     Colonna V, Nutile T, Ferrucci RR, Fardella G, Aversano M, et al. (2009) Comparing population structure as inferred from genealogical versus genetic information. Eur J Hum Genet 17(12): 1635-1641.

18.     Ciullo M, Nutile T, Dalmasso C, Sorice R, Bellenguez C, et al. (2008) Identification and replication of a novel obesity locus on chromosome 1q24 in isolated populations of cilento. Diabetes 57(3): 783-790.

19.     Traglia M, Sala C, Masciullo C, Cverhova V, Lori F, et al. (2009) Heritability and demographic analyses in the large isolated population of val borbera suggest advantages in mapping complex traits genes. PLoS One 4(10): e7554.

20.     Heid IM, Huth C, Loos RJ, Kronenberg F, Adamkova V, et al. (2009) Meta-analysis of the INSIG2 association with obesity including 74,345 individuals: Does heterogeneity of estimates relate to study design? PLoS Genet 5(10): e1000694.

21.     Colonna V, Nutile T, Astore M, Guardiola O, Antoniol G, et al. (2007) Campora: A young genetic isolate in south italy. Hum Hered 64(2): 123-135.

22.     Ridker PM, Danielson E, Fonseca FA, Genest J, Gotto AM,Jr, et al. (2008) Rosuvastatin to prevent vascular events in men and women with elevated C-reactive protein. N Engl J Med 359(21): 2195-2207.

23.     Portas L, Murgia F, Biino G, Concas MP, Casula L, et al. (2010) History, geography and population structure influence the distribution and heritability of blood and anthropometric quantitative traits in nine sardinian genetic isolates. Genet Res (Camb) 92(3): 199-208.

24.     Biino G, Casula L, de Terlizzi F, Adamo M, Vaccargiu S, et al. (2010) Genetic architecture of hand quantitative ultrasound measures: A population-based study in a sardinian genetic isolate. Bone 46(4): 1197-1203.

25.     Pistis G, Piras I, Pirastu N, Persico I, Sassu A, et al. (2009) High differentiation among eight villages in a secluded area of sardinia revealed by genome-wide high density SNPs analysis. PLoS One 4(2): e4654.

26.     Shepherd J, Blauw GJ, Murphy MB, Bollen EL, Buckley BM, et al. (2002) Pravastatin in elderly individuals at risk of vascular disease (PROSPER): A randomised controlled trial. Lancet 360(9346): 1623-1630.

27.     Shepherd J, Blauw GJ, Murphy MB, Cobbe SM, Bollen EL, et al. (1999) The design of a prospective study of pravastatin in the elderly at risk (PROSPER). PROSPER study group. PROspective study of pravastatin in the elderly at risk. Am J Cardiol 84(10): 1192-1197.

28.     Ford I, Bezlyak V, Stott DJ, Sattar N, Packard CJ, et al. (2009) Reduced glomerular filtration rate and its association with clinical outcome in older patients at risk of vascular events: Secondary analysis. PLoS Med 6(1): e16.

29.     Downs SH, Schindler C, Liu LJ, Keidel D, Bayer-Oglesby L, et al. (2007) Reduced exposure to PM10 and attenuated age-related decline in lung function. N Engl J Med 357(23): 2338-2347.

30.     Nitsch D, Felber Dietrich D, von Eckardstein A, Gaspoz JM, Downs SH, et al. (2006) Prevalence of renal impairment and its association with cardiovascular risk factors in a general population: Results of the swiss SAPALDIA study. Nephrol Dial Transplant 21(4): 935-944.

31.     Imboden M, Downs SH, Senn O, Matyas G, Brandli O, et al. (2007) Glutathione S-transferase genotypes modify lung function decline in the general population: SAPALDIA cohort study. Respir Res 8: 2.

32.     Probst-Hensch NM, Imboden M, Felber Dietrich D, Barthelemy JC, Ackermann-Liebrich U, et al. (2008) Glutathione S-transferase polymorphisms, passive smoking, obesity, and heart rate variability in nonsmokers. Environ Health Perspect 116(11): 1494-1499.

33.     Heid IM, Wagner SA, Gohlke H, Iglseder B, Mueller JC, et al. (2006) Genetic architecture of the APM1 gene and its influence on adiponectin plasma levels and parameters of the metabolic syndrome in 1,727 healthy caucasians. Diabetes 55(2): 375-384.

34.     Kollerits B, Coassin S, Kiechl S, Hunt SC, Paulweber B, et al. (2010) A common variant in the adiponutrin gene influences liver enzyme values. J Med Genet 47(2): 116-119.
