# Supplementary material for: Genome-Wide Association and Functional Follow-Up Reveals New Loci for Kidney Function
Source: PLoS Genet. 2012 Mar 29;8(3):e1002584. doi: 10.1371/journal.pgen.1002584 (PMC3315455; doi:10.1371/journal.pgen.1002584)
Supplement: Table S3 — Characteristics of stage 1 discovery studies. (DOC) [file pgen.1002584.s015.doc]

**Table S3. Characteristics of stage 1 discovery studies.**

| **Study** | **Stratum (include only subjects from the specified stratum)** | **Sample Size eGFRcrea / CKD / eGFRcys** | **Women:**  **%(n)** | **Age**  **[years]**  **mean(SD)** | **eGFRcrea [ml/min/ 1.73 m^2^]**  **mean(SD)** | **eGFRcys [ml/min/ 1.73 m^2^]**  **mean(SD)** | **CKD**  **%(n)** | **DM**  **%(n)** | **HTN**  **%(n)** | **CKD45**  **%(n)** |
| --- | --- | --- | --- | --- | --- | --- | --- | --- | --- | --- |
| **AGES** | all | 3219/3219/NA | 58.0(1867) | 76.4(5.46) | 73.0(20.0) | NA | 24.2(781) | 11.5(368) | 80.6(2595) | 7.5(199) |
|  | no HTN | 623/623/NA | 55.5(346) | 74.8(5.23) | 78.8(19.2) | NA | 12.8(80) | 5.5(343) | - | 2.3(13) |
|  | HTN | 2595/2595/NA | 58.6(185) | 76.8(5.44) | 71.6(20.0) | NA | 27.0(700) | 12.9(334) | 100(2595) | 8.9(185) |
|  | no DM | 2847/2847/NA | 59.7(1701) | 76.4(5.49) | 73.3(19.8) | NA | 23.6(671) | - | 79.3(2257) | 6.8(160) |
|  | DM | 368/368/NA | 44.8(165) | 76.3(5.13) | 70.7(21.5) | NA | 29.4(108) | 100(368) | 90.8(334) | 12.8(38) |
|  | older age | 3219/3219/NA | 58.0(1867) | 76.4(5.46) | 73.0(20.0) | NA | 24.2(781) | 11.5(368) | 80.6(2595) | 7.5(199) |
|  | younger age | 0/0/NA | NA | NA | NA | NA | NA | NA | NA | NA |
|  | women | 1867/1867/NA | 100(1867) | 76.3(5.55) | 72.8(20.8) | NA | 25.9(483) | 8.8(165) | 81.4(1520) | 7.5(113) |
|  | men | 1352/1352/NA | - | 76.5(5.32) | 73.2(18.9) | NA | 22.0(298) | 15.1(203) | 79.5(1075) | 7.5(86) |
| **Amish Studies** | all | 1211/NA/783 | 48.9(592) | 49.5(16.9) | 93.7(19.7) | 114.9(18.0) | 3.1(37) | 1.7(20) | 18.9(229) | 0.4(5) |
|  | no HTN | 982/NA/656 | 47.2(463) | 46.7(16.1) | 95.3(19.2) | 116.0(18.1) | 1.8(18) | 0.6(6) | - | 0.3(3) |
|  | HTN | 229/NA/127 | 56.3(129) | 61.3(15.1) | 86.7(20.2) | 108.8(16.6) | 8.74(19) | 6.3(14) | 100(229) | 0.9(2) |
|  | no DM | 1191/NA/779 | 48.7(580) | 49.2(16.7) | 93.8(19.6) | 115.0(18.0) | 2.9(34) | - | 18.1(215) | 0.3(4) |
|  | DM | 20/NA/4 | 60.0(12) | 70.1(15.2) | 83.5(23.9) | 92.0(11.7) | 16.7(3) | 100(20) | 70.0(14) | 5.6(1) |
|  | older age | 230/NA/55 | 48.7(112) | 73.7(8.9) | 82.2(20.9) | 103.7(17.5) | 11.2(25) | 5.8(13) | 42.2(97) | 1.8(4) |
|  | younger age | 981/NA/728 | 48.9(480) | 43.8(12.8) | 96.3(18.4) | 115.7(17.8) | 1.2(12) | 0.7(7) | 13.5(132) | 0.1(1) |
|  | women | 592/NA/358 | 100(592) | 50.6(16.3) | 88.7(18.3) | 117.6(19.8) | 4.6(27) | 2.1(12) | 21.8(129) | 0.3(2) |
|  | men | 619/NA/425 | - | 48.4(17.4) | 98.5(19.8) | 112.6(16.1) | 1.6(10) | 1.3(8) | 16.2(100) | 0.5(3) |
| **ARIC** | all | 8982/8982/7145 | 53.1(4767) | 61.8(6.1) | 81.4(17.5) | 84.2(19.7) | 8.7(782) | 14.2(1276) | 40.7(3643) | 1.5(121) |
|  | no HTN | 5302/5302/4097 | 53.9(2857) | 60.8(6.0) | 82.7(16.5) | 87.0(19.0) | 5.9(314) | 10.0(532) | - | 0.7(33) |
|  | HTN | 3643/3643/2964 | 52.1(1897) | 63.2(5.9) | 79.5(18.7) | 80.5(19.9) | 12.8(467) | 20.3(739) | 100(3643) | 2.7(87) |
|  | no DM | 7697/6900/6122 | 54.6(4197) | 61.6(6.1) | 81.1(16.9) | 84.7(19.3) | 8.3(636) | - | 37.8(2896) | 1.1(79) |
|  | DM | 1276/1276/959 | 44.4(566) | 62.6(5.9) | 83.0(20.9) | 81.3(21.8) | 11.4(145) | 100(1276) | 58.1(739) | 3.6(42) |
|  | older age | 2658/2658/2458 | 48.9(1300) | 69.1(2.3) | 78.6(16.6) | 79.3(18.0) | 12.6(335) | 16.8(445) | 52.8(1398) | 1.5(35) |
|  | younger age | 6324/6324/4635 | 53.8(3467) | 58.7(4.3) | 82.6(17.7) | 86.9(20.0) | 7.1(447) | 13.2(831) | 35.6(2245) | 1.4(86) |
|  | women | 4767/4767/3801 | 100(4767) | 61.4(6.1) | 80.5(17.4) | 86.9(20.3) | 9.0(431) | 11.9(566) | 39.9(1897) | 1.4(63) |
|  | men | 4215/4215/3292 | - | 62.2(6.1) | 82.5(17.5) | 81.1(17.4) | 8.3(351) | 16.9(710) | 41.7(1746) | 1.5(58) |
| **ASPS** | all | 848/848/NA | 56.8(482) | 65.2(8.0) | 96.5(39.9) | NA | 8.1(69) | 9.2(78) | 72.5(615) | 0.9(8) |
|  | no HTN | 233/233/NA | 57.9(135) | 61.2(6.9) | 91.7(29.7) | NA | 6.8(16) | 5.1(12) | - | 0.8(2) |
|  | HTN | 615/615/NA | 56.4(347) | 66.6(7.9) | 98.4(43.0) | NA | 8.6(53) | 10.7(66) | 100(615) | 0.9(6) |
|  | no DM | 770/770/NA | 57.1(440) | 64.7(7.9) | 95.7(38.4) | NA | 7.4(57) | - | 71.3(549) | 0.7(6) |
|  | DM | 78/78/NA | 53.8(42) | 70.0(7.3) | 104.8(51.5) | NA | 15.3(12) | 100(78) | 84.6(66) | 2.5(2) |
|  | older age | 394/394/NA | 60.4(238) | 72.3(4.7) | 104.7(52.3) | NA | 12.6(50) | 14.4(57) | 85.7(338) | 1.2(5) |
|  | younger age | 454/454/NA | 53.7(244) | 58.9(4.2) | 89.5(22.3) | NA | 4.1(19) | 4.6(21) | 61.0(277) | 0.6(3) |
|  | women | 482/482/NA | 100(482) | 65.5(8.2) | 82.7(22.8) | NA | 11.8(57) | 8.7(42) | 71.9(347) | 1.2(6) |
|  | men | 366/366/NA | - | 64.6(7.8) | 114.7(49.2) | NA | 3.2(12) | 9.8(36) | 73.2(268) | 0.5(2) |
| **Baltimore Longitudinal Study of Aging (BLSA)** | all | 723/723/NA | 46.1(333) | 70.4(15.2) | 80.3(23.1) | NA | 17.4(126) | 7.7(55) | 21.9(147) | 5.5(40) |
|  | no HTN | 525/525/NA | 48.0(252) | 68.1(15.4) | 82.7(22.6) | NA | 14.3(75) | 0.0(0) | - | 3.6(19) |
|  | HTN | 147/147/NA | 36.7(54) | 75.2(12.8) | 73.8(24.3) | NA | 27.2(40) | 36.6(53) | 100(147) | 12.2(18) |
|  | no DM | 662/662/NA | 47.9(317) | 70.1(15.5) | 80.9(23.0) | NA | 16.3(108) | - | 15.0(92) | 5(33) |
|  | DM | 55/55/NA | 23.6(13) | 75.0(10.6) | 73.2(24.2) | NA | 30.9(17) | 100(55) | 96.4(53) | 12.7(7) |
|  | older age | 457/457/NA | 40.0(183) | 80.0(7.8) | 75.0(22.7) | NA | 24.1(110) | 10.2(46) | 28.9(119) | 8.8(40) |
|  | younger age | 266/266/NA | 56.4(150) | 53.9(9.7) | 89.5(21.0) | NA | 6.0(16) | 3.4(9) | 10.8(28) | 0.0(0) |
|  | women | 333/333/NA | 100(333) | 68.4(15.8) | 80.9(23.1) | NA | 16.8(56) | 3.9(13) | 17.7(54) | 3.3(11) |
|  | men | 390/390/NA | - | 72.1(14.6) | 79.8(23.2) | NA | 18.0(70) | 10.9(42) | 25.4(93) | 7.4(29) |
| **Cardiovascular Health Study (CHS)** | all | 2820/2353/2475 | 61.3(1729) | 71.9(5.0) | 77.3(20.8) | 81.0(17.9) | 9.5(224) | 11.0(307) | 51.4(1441) | 2.8(67) |
|  | no HTN | 1356/1189/1180 | 59.4(809) | 71.4 (4.8) | 78.6(20.2) | 84.2(17.5) | 6.2(74) | 6.7(91) | - | 1.6(19) |
|  | HTN | 1441/1151/1280 | 37.0(533) | 72.4(5.2) | 76.1(21.3) | 78.1(17.7) | 13.0(149) | 15.1(216) | 100(1441) | 4.0(48) |
|  | no DM | 2488/2078/2191 | 37.6(935) | 71.9(5.0) | 76.7(20.1) | 81.2(17.7) | 9.3(194) | - | 49.0(1219) | 2.7(58) |
|  | DM | 307/255/261 | 48.2(148) | 72.1(5.1) | 82.6(25.7) | 79.3(19.0) | 11.4(29) | 100(307) | 70.4(216) | 3.4(9) |
|  | older age | 2820/2353/2475 | 61.3(1729) | 71.9(5.0) | 77.3(20.8) | 81.0(17.9) | 9.5(224) | 11.0(307) | 51.4(1441) | 2.8(67) |
|  | younger age | 0/0/NA | NA | NA | NA | NA | NA | NA | NA | NA |
|  | women | 1729/1426/1624 | 100(1729) | 71.6(4.8) | 76.4(20.4) | 82.6(18.4) | 10.0(142) | 9.3(159) | 52.9(908) | 2.9(42) |
|  | men | 1091/927/851 | - | 72.5(5.3) | 78.7(21.4) | 77.9(16.4) | 8.9(82) | 13.7(148) | 49.1(533) | 2.7(25) |
| **ERF** | all | 2079/2079/NA | 56.3(1171) | 49.2(14.0) | 93.5(21.4) | NA | 3.7(76) | 6.6(138) | 52.5(1092) | 0.6(12) |
|  | no HTN | 984/984/NA | 64.7(637) | 42.4(11.7) | 98.7(20.8) | NA | 1.2(12) | 1.5(15) | - | 0.1(1) |
|  | HTN | 1092/1092/NA | 48.6(531) | 55.3(13.1) | 88.7(20.8) | NA | 5.9(64) | 11.2(122) | 100(1092) | 1.0(11) |
|  | no DM | 1911/1911/NA | 57.4(1096) | 48.5(13.9) | 93.7(21.2) | NA | 3.4(65) | - | 50.0(955) | 0.5(9) |
|  | DM | 138/138/NA | 47.1(65) | 60.0(11.3) | 89.5(23.5) | NA | 8.0(11) | 100(138) | 88.4(122) | 2.2(3) |
|  | older age | 296/296/NA | 52.4(155) | 70.8(4.7) | 74.9(16.5) | NA | 17.2(51) | 15.2(45) | 88.9(263) | 3.0(9) |
|  | younger age | 1783/1783/NA | 57.0(1016) | 45.6(11.6) | 96.5(20.5) | NA | 1.4(25) | 5.2(93) | 46.5(829) | 0.2(3) |
|  | women | 1171/1171/NA | 100(1171) | 48.6(13.9) | 93.5(22.5) | NA | 4.2(49) | 5.6(65) | 45.3(531) | 0.3(4) |
|  | men | 908/908/NA | - | 49.9(14.1) | 93.4(19.9) | NA | 3.0(27) | 8.0(73) | 61.8(561) | 0.9(8) |
| **Family Heart Study (FamHS)** | all | 883/883/NA | 51.1(451) | 55.5(11.1) | 88.5(19.4) | NA | 4.4(39) | 7.1(62) | 30.1(266) | 0.2(2) |
|  | no HTN | 617/617/NA | 48.1(297) | 53.3(11.4) | 90.2(18.9) | NA | 2.6(16) | 4.4(27) | - | 0.0(0) |
|  | HTN | 266/266/NA | 57.9(154) | 60.4(8.4) | 84.5(19.8) | NA | 8.7(23) | 13.3(35) | 100(266) | 0.8(2) |
|  | no DM | 812/812/NA | 51.4(417) | 55.2(11.1) | 88.0(18.5) | NA | 4.4(36) | - | 4.4(27) | 0.1(1) |
|  | DM | 62/62/NA | 50.0(31) | 59.7(9.8) | 93.8(27.3) | NA | 4.8(3) | 100(62) | 13.3(35) | 1.6(1) |
|  | older age | 178/178/NA | 55.6(99) | 69.0(3.7) | 78.1(14.9) | NA | 10.1(18) | 11.2(20) | 46.1(82) | 1.1(2) |
|  | younger age | 705/705/NA | 49.9( 352) | 52.0(9.6) | 91.1(19.5) | NA | 3.0(21) | 6.0(42) | 26.1(184) | 0.0(0) |
|  | women | 451/451/NA | 100(451) | 57.8(9.4) | 86.2(19.6) | NA | 6.9(31) | 6.9(31) | 34.2(154) | 0.0(0) |
|  | men | 432/432/NA | - | 53.0(12.1) | 90.9(18.9) | NA | 1.9(8) | 7.3(31) | 25.9(112) | 0.4(2) |
| **Framingham Heart Study (FHS)** | all | 7782/4140/2992 | 54.3(4229) | 51.2(14.0) | 91.7(21.7) | 83.8(17.8) | 10.8(445) | 6.4(496) | 29.3(2283) | 1.3(103) |
|  | no HTN | 5499/1766/1635 | 56.7(3118) | 47.8(13.4) | 94.8(20.8) | 88.4(16.3) | 6.2(110) | 3.0(164) | - | 0.6(33) |
|  | HTN | 2283/2374/1357 | 48.7(1111) | 59.4 911.9) | 85.7(22.5) | 78.2(17.9) | 14.1(335) | 14.5(332) | 100(2283) | 3.1(70) |
|  | no DM | 7286/3743/2662 | 55.1(4012) | 50.5(13.9) | 92.6(21.4) | 84.7(17.3) | 10.1(377) | - | 26.8(1951) | 1.1(82) |
|  | DM | 496/406/330 | 43.8(217) | 60.9(11.6) | 86.0(25.0) | 76.2(19.4) | 16.8(68) | 100(496) | 66.9(332) | 4.2(21) |
|  | older age | 1378/2089/1002 | 57.0(785) | 71.5(4.4) | 77.8(22.7) | 74.1(17.0) | 16.8(350) | 14.0(193) | 56.2(775) | 5.2(71) |
|  | younger age | 6404/1051/1990 | 53.8(3444) | 46.8(11.3) | 95.2(20.2) | 88.7(16.0) | 4.6(95) | 4.7(303) | 23.6(1508) | 0.5(32) |
|  | women | 4229/2258/1609 | 100(4229) | 51.4(14.1) | 91.4(22.7) | 86.2(18.3) | 12.9(292) | 5.1(217) | 26.7(1111) | 1.7(72) |
|  | men | 3553/1882/1383 | - | 50.9(13.9) | 93.0(20.3) | 81.1(16.7) | 8.1(153) | 7.9(279) | 33.0(1132) | 0.9(31) |
| **GENOA** | all | 1163/1163/NA | 56.3(655) | 59(10.2) | 87.7(24) | NA | 10.7(125) | 15.3(178) | 73.3(852) | 2.3(1163) |
|  | no HTN | 311/311/NA | 59.8(186) | 53.2(10.1) | 94.3(21.9) | NA | 4.5(14) | 6.1(19) | - | 0.3(311) |
|  | HTN | 852/852/NA | 55(469) | 61.2(9.4) | 85.3(24.3) | NA | 13(111) | 18.7(159) | 100(852) | 3.1(852) |
|  | no DM | 985/985/NA | 57.7(568) | 58.4(10.2) | 88.7(23) | NA | 8.6(85) | - | 70.4(693) | 1.3(985) |
|  | DM | 178/178/NA | 48.9(87) | 62.5(9.3) | 82.4(28.1) | NA | 22.5(40) | 100(178) | 89.3(159) | 7.9(178) |
|  | older age | 347/347/NA | 52.2(181) | 70.9(4.3) | 77.7(25.2) | NA | 23.9(83) | 22.2(77) | 88.2(306) | 5.8(347) |
|  | younger age | 816/816/NA | 58.1(474) | 54(7.4) | 92(22.2) | NA | 5.1(42) | 12.4(101) | 66.9(546) | 0.9(816) |
|  | women | 655/655/NA | 100(655) | 58.6(10.3) | 87.1(24.1) | NA | 11.5(75) | 13.3(87) | 71.6(469) | 2.9(655) |
|  | men | 508/508/NA | - | 59.6(10.1) | 88.5(23.8) | NA | 9.8(50) | 17.9(91) | 75.4(383) | 1.6(508) |
| **Health ABC** | all | 1663/1663/1663 | 47.1(784) | 73.8(2.8) | 71.2(14.8) | 77.0(19.9) | 25.0(415) | 13.0(216) | 63.7(1060) | 4.3(56) |
|  | no HTN | 603/603/603 | 45.8(276) | 73.6(2.8) | 73.0(13.6) | 79.8(19.1) | 19.4(117) | 9.3(56) | - | 2.4(12) |
|  | HTN | 1060/1060/1060 | 47.9(508) | 73.9(2.9) | 70.2(15.3) | 75.5(20.2) | 28.1(298) | 15.1(160) | 100(1060) | 5.5(44) |
|  | no DM | 1447/1447/1447 | 50.2(726) | 73.8(2.8) | 71.2(14.4) | 77.8(19.8) | 24.5(355) | - | 62.2(900) | 3.9(44) |
|  | DM | 216/216/216 | 26.9(58) | 73.8(2.9) | 71.3(17.4) | 71.6(20.2) | 27.8(60) | 100(216) | 74.1(160) | 7.1(12) |
|  | older age | 1663/1663/1663 | 47.1(784) | 73.8(2.8) | 71.2(14.8) | 77.0(19.9) | 25.0(415) | 13.0(216) | 63.7(1060) | 4.3(56) |
|  | younger age | 0/0/NA | NA | NA | NA | NA | NA | NA | NA | NA |
|  | women | 784/784/784 | 100(784) | 73.6(2.8) | 70.5(14.2) | 80.7(21.1) | 29.6(232) | 7.4(58) | 64.8(508) | 4.2(24) |
|  | men | 879/879/879 | - | 73.9(2.9) | 71.9(15.3) | 73.7(18.2) | 20.8(183) | 18.0(158) | 62.8(552) | 4.4(32) |
| **Health Professionals Follow-Up Study (HPFS)** | all | 818/818/NA | - | 64.7(8.3) | 85.2(22.7) | NA | 9.5(78) | 100(818) | 59(479) | NA |
|  | no HTN | 339/339/NA | - | 63.2(8.8) | 88.4(21.5) | NA | 5.0(17) | 100(339) | - | NA |
|  | HTN | 479/479/NA | - | 65.8(7.7) | 82.9(23.2) | NA | 12.7(61) | 100(479) | 100(479) | NA |
|  | no DM | 0/0/NA | NA | NA | NA | NA | NA | NA | NA | NA |
|  | DM | 818/818/NA | - | 64.7(8.3) | 85.2(22.7) | NA | 9.5(78) | 100(818) | 59(479) | NA |
|  | older age | 433/433/NA | - | 71.2(3.9) | 76.9(18.5) | NA | 14.6(63) | 100(433) | 64.4(279) | NA |
|  | younger age | 385/385/NA | - | 57.4(5.3) | 94.5(23.4) | NA | 3.9(15) | 100(385) | 52.0(200_ | NA |
|  | women | 0/0/NA | NA | NA | NA | NA | NA | NA | NA | NA |
|  | men | 818/818/NA | 0.0(0) | 64.7(8.3) | 85.2(22.7) | NA | 9.5(78) | 100(818) | 59(479) | NA |
| **KORA F3** | all | 1641/1641/1642 | 50.5(831) | 62.5(10.1) | 83.9(21) | 111.8(26.3) | 10.8(177) | 11.1(179) | 41.1(674) | 2.5(41) |
|  | no HTN | 963/963/964 | 55.9(539) | 61.3(10.7) | 85.6(21.7) | 113.9(27.1) | 10.7(103) | 10.7(102) | - | 2.5(24) |
|  | HTN | 673/673/673 | 42.6(287) | 64.3(8.8) | 81.5(19.6) | 108.8(24.7) | 11(74) | 11.5(76) | 100(674) | 2.5(17) |
|  | no DM | 1435/1435/1436 | 51.8(745) | 61.9(10.2) | 85.2(20.6) | 113.5(25.2) | 8.9(127) | - | 40.7(584) | 1.8(26) |
|  | DM | 179/179/179 | 43(77) | 67.9(7.3) | 73.9(21.7) | 98(30.1) | 26.3(47) | 100(179) | 42.7(76) | 8.4(15) |
|  | older age | 676/676/676 | 49.5(335) | 71.6(4.1) | 74.8(17.5) | 97.3(22.8) | 19.1(129) | 16.2(108) | 46.7(315) | 4.3(29) |
|  | younger age | 965/965/966 | 51.3(496) | 56.1(7.9) | 90.3(20.8) | 122(23.7) | 5(48) | 7.5(71) | 37.2(359) | 1.2(12) |
|  | women | 828/828/829 | 100(831) | 62.1(10.1) | 82.6(20.8) | 115.1(27) | 12(99) | 9.4(77) | 34.7(287) | 2.7(22) |
|  | men | 813/813/813 | - | 63(10.1) | 85.2(21.1) | 108.5(25.2) | 9.6(78) | 12.8(102) | 47.6(387) | 2.3(19) |
| **KORA F4** | all | 1814/1814/1811 | 51.3(930) | 60.9(8.9) | 85.1(20.2) | 109.7(26.2) | 7(127) | 9.2(167) | 20.9(379) | 1.5(28) |
|  | no HTN | 1432/1432/1431 | 55.9(801) | 60.4(8.9) | 85.4(20.3) | 110.7(25.9) | 7(100) | 8.5(121) | - | 1.7(24) |
|  | HTN | 379/379/377 | 33.5(127) | 62.6(8.6) | 83.8(19.9) | 105.9(27.2) | 6.9(26) | 12.1(46) | 100(379) | 1.1(4) |
|  | no DM | 1646/1646/1643 | 51.9(854) | 60.3(8.8) | 85.9(19.8) | 111(26) | 5.8(96) | - | 20.3(333) | 1.3(21) |
|  | DM | 167/167/167 | 45.5(76) | 66.8(7.4) | 76.7(22.7) | 96.8(25.6) | 18.6(31) | 100(167) | 27.5(46) | 4.2(7) |
|  | older age | 622/622/620 | 50.3(313) | 70.9(3.3) | 74.9(18.2) | 97.9(26.5) | 16.4(102) | 16.9(105) | 24.2(150) | 3.5(22) |
|  | younger age | 1192/1192/1191 | 51.8(617) | 55.7(5.9) | 90.4(19.1) | 115.8(23.9) | 2.1(25) | 5.2(62) | 19.2(229) | 0.5(6) |
|  | women | 930/930/928 | 100(930) | 60.6(8.8) | 84.1(20.2) | 112.2(26.3) | 7.5(70) | 8.2(76) | 13.7(127) | 1.7(16) |
|  | men | 884/884/883 | - | 61.2(8.9) | 86.1(20.2) | 107(25.9) | 6.4(57) | 10.3(91) | 28.5(252) | 1.4(12) |
| **Korcula** | all | 888/888/NA | 64.0(568) | 56.3(13.9) | 87.3(20.6) | NA | 7.5(67) | 13.1(116) | 54.2(474) | 0.8(7) |
|  | no HTN | 400/400/NA | 71.3(285) | 49.3(13.1) | 91.0(20.4) | NA | 4.8(19) | 5.2(21) | - | 0.3(1) |
|  | HTN | 474/474/NA | 57.6(273) | 62.1(11.5) | 84.2(20.2) | NA | 9.5(45) | 19.8(94) | 100(474) | 1.3(6) |
|  | no DM | 772/772/NA | 66.1(510) | 54.9(13.8) | 88.0(20.4) | NA | 6.5(50) | - | 50.1(380) | 0.6(5) |
|  | DM | 116/116/NA | 50.0(58) | 65.2(10.5) | 82.1(21.5) | NA | 14.7(17) | 100(116) | 81.7(94) | 1.7(2) |
|  | older age | 243/243/NA | 54.7(133) | 73.0(5.7) | 71.8(14.5) | NA | 21.4(52) | 23.9(58) | 82.3(195) | 2.5(6) |
|  | younger age | 645/645/NA | 67.4(435) | 50.0(10.4) | 93.1(19.5) | NA | 2.3(15) | 9.0(58) | 43.8(279) | 0.2(1) |
|  | women | 568/568/NA | 100(568) | 55.5(13.6) | 87.3(20.8) | NA | 7.0(40) | 10.2(58) | 48.9(273) | 1.1(6) |
|  | men | 320/320/NA | - | 57.6(14.3) | 87.1(20.3) | NA | 8.4(27) | 18.1(58) | 63.6(201) | 0.3(1) |
| **Microisolates in South Tyrol (MICROS)** | all | 1201/1201/1198 | 56.5(678) | 46.2(16.1) | 94.6(20.9) | 107.4(23.8) | 3.8(46) | 4.3(49) | 37.7(437) | 0.6(7) |
|  | no HTN | 721/721/720 | 59.6(430) | 40.8(13.6) | 98.8(20.3) | 112.3(21.8) | 2.1(15) | 1.7(12) | - | 0.1(1) |
|  | HTN | 437/437/435 | 51.3(224) | 54.9(15.8) | 87.8(19.8) | 99.3(24.7) | 6.4(28) | 8.5(36) | 100(437) | 1.1(5) |
|  | no DM | 1099/1099/1096 | 56.6(622) | 45.3(15.7) | 94.9(20.6) | 108.1(23.3) | 3.7(41) | - | 35.5(387) | 0.6(7) |
|  | DM | 49/49/49 | 53.1(26) | 63.5(12.1) | 86.8(24.4) | 93.8(26.1) | 6.1(3) | 100(49) | 75.0(36) | 0.0(0) |
|  | older age | 201/201/201 | 59.2(119) | 72.7(5.3) | 73.5(15.5) | 81.6(21.8) | 18.9(38) | 14.7(28) | 73.6(142) | 2.5(5) |
|  | younger age | 1000/1000/997 | 55.9(559) | 40.9(11.6) | 98.8(19.2) | 112.6(20.6) | 0.8(8) | 2.2(21) | 30.6(295) | 0.2(2) |
|  | women | 678/678/675 | 100(678) | 46.5(16.4) | 94.1(22.0) | 109.8(25.5) | 4.0(27) | 4.0(26) | 34.3(224) | 0.7(5) |
|  | men | 523/523/523 | - | 45.8(15.6) | 95.1(19.4) | 104.4(21.1) | 3.6(19) | 4.6(23) | 42.3(213) | 0.4(2) |
| **Northern Sweden Population Health Survey (NSPHS)** | all | 565/565/NA | 53.1(300) | 51.7(18.3) | 91.0(22.1) | NA | 5.7(32) | 7.8(44) | 43.4(242) | 1.9(11) |
|  | no HTN | 316/316/NA | 56.0(177) | 41.0(14.0) | 99.1(20.2) | NA | 0.9(3) | 2.5(8) | - | 0.3(1) |
|  | HTN | 242/242/NA | 50.4(122) | 65.8(13.0) | 80.1(20.1) | NA | 12.0(29) | 14.9(36) | 100(242) | 4.1(10) |
|  | no DM | 521/521/NA | 53.0(276) | 50.4(18.1) | 91.7(21.9) | NA | 5.0(26) | - | 40.1(206) | 1.5(8) |
|  | DM | 44/44/NA | 54.5(24) | 66.8(12.3) | 82.3(23.0) | NA | 13.6(6) | 100(44) | 81.8(36) | 6.8(3) |
|  | older age | 158/158/NA | 52.5(83) | 74.7(6.0) | 72.9(16.1) | NA | 17.1(27) | 17.1(27) | 88.6(140) | 5.7(9) |
|  | younger age | 407/407/NA | 53.3(217) | 42.8(12.8) | 97.9(20.1) | NA | 1.2(5) | 4.2(17) | 25.5(102) | 0.5(2) |
|  | women | 300/300/NA | 100(300) | 50.9(18.4) | 90.9(22.7) | NA | 4.7(14) | 8.0(24) | 40.8(122) | 1.7(5) |
|  | men | 265/265/NA | - | 52.5(18.1) | 91.0(21.5) | NA | 6.8(18) | 7.5(20) | 46.3(120) | 2.3(6) |
| **Nurses' Health Study (NHS)** | all | 786/786/NA | 100(786) | 59.5(6.5) | 86.2(22.1) | NA | 10.7(84) | 100(786) | 70(554) | NA |
|  | no HTN | 232/232/NA | 100(232) | 57.9(6.7) | 88.2(21.6) | NA | 5.6(13) | 100(232) | - | NA |
|  | HTN | 554/554/NA | 100(554) | 60.2(6.3) | 85.4(22.2) | NA | 12.87(71) | 100(554) | 100(554) | NA |
|  | no DM | 0/0/NA | NA | NA | NA | NA | NA | NA | NA | NA |
|  | DM | 786/786/NA | 100(786) | 59.5(6.5) | 86.2(22.1) | NA | 10.7(84) | 100(786) | 70.0(554) | NA |
|  | older age | 184/184/NA | 100(184) | 67.0(1.3) | 77.2(19.3) | NA | 17.9(33) | 100(184) | 81.0(149) | NA |
|  | younger age | 602/602/NA | 100(602) | 57.2(5.6) | 89.0(22.1) | NA | 8.5(51) | 100(602) | 67.3(405) | NA |
|  | women | 786/786/NA | 100(786) | 59.5(6.5) | 86.2(22.1) | NA | 10.7(84) | 100(786) | 70.0(554) | NA |
|  | men | 0/0/NA | NA | NA | NA | NA | NA | NA | NA | NA |
| **Orkney Complex Disease Study (ORCADES)** | all | 704/704/NA | 53.6(377) | 54.2(15.2) | 89.4(20.7) | NA | 6.8(48) | 4.0(28) | 41.8(287) | 1.4(10) |
|  | no HTN | 399/399/NA | 56.9(227) | 46.9(12.9) | 95.0(18.9) | NA | 1.5(6) | 1.3(5) | - | 0.3(1) |
|  | HTN | 287/287/NA | 48.4(139) | 64.6(10.7) | 81.4(20.3) | NA | 13.9(40) | 8.0(23) | 100(287) | 2.8(8) |
|  | no DM | 675/675/NA | 54.5(368) | 53.7(15.2) | 89.7(20.4) | NA | 6.1(41) | - | 40.1(264) | 1.0(7) |
|  | DM | 28/28/NA | 32.1(9) | 62.8(10.6) | 82.2(26.7) | NA | 25.0(7) | 100(28) | 82.1(23) | 10.7(3) |
|  | older age | 186/186/NA | 51.1(95) | 72.8(5.9) | 73.9(19.2) | NA | 21.0(39) | 7.0(13) | 77.9(141) | 3.8(7) |
|  | younger age | 518/518/NA | 54.4(282) | 47.5(11.5) | 95.0(18.3) | NA | 1.7(9) | 2.9(15) | 28.9(146) | 0.6(3) |
|  | women | 377/377/NA | 100(377) | 53.7(15.1) | 89.9(21.9) | NA | 7.4(28) | 2.4(9) | 38.0(139) | 2.1(8) |
|  | men | 327/327/NA | - | 54.7(15.3) | 88.8(19.3) | NA | 6.1(20) | 5.8(19) | 46.3(148) | 0.6(2) |
| **Popgen** | all | 1163/1163/NA | 44.4(516) | 54.8(13.9) | 88.1(18.8) | NA | 5.1(59) | 3.8(44) | 46.8(541) | 0.3(4) |
|  | no HTN | 614/614/NA | 52.9(325) | 51.7(14.6) | 89.9(19.2) | NA | 4.7(29) | 2.6(16) | - | 0.2(1) |
|  | HTN | 541/541/NA | 34.9(189) | 58.2(12.2) | 86.2(18.2) | NA | 5.5(30) | 5.2(28) | 100(614) | 0.6(3) |
|  | no DM | 1116/1116/NA | 45.2(504) | 54.3(13.9) | 88.4(18.8) | NA | 4.7(53) | - | 46.0(510) | 0.4(4) |
|  | DM | 44/44/NA | 25.0(11) | 66.0(6.3) | 82.0(18.7) | NA | 11.4(5) | 100(44) | 63.6(28) | 0.0(0) |
|  | older age | 304/304/NA | 47.0(143) | 69.9(3.3) | 74.6(14.7) | NA | 13.2(40) | 9.9(30) | 56.7(170) | 1.3(4) |
|  | younger age | 859/859/NA | 43.4(373) | 49.5(12.1) | 92.9(17.8) | NA | 2.2(19) | 1.6(14) | 43.4(371) | 0.0(0) |
|  | women | 516/516/NA | 100(516) | 54.5(14.6) | 86.9(19.5) | NA | 6.2(32) | 2.1(11) | 36.8(189) | 0.4(2) |
|  | men | 647/647/NA | - | 55.1(13.3) | 89.0(18.3) | NA | 4.2(27) | 5.1(33) | 54.9(352) | 0.3(2) |
| **Rotterdam Study – I** | all | 4390/4390/NA | 61.4(2696) | 70.0(9.0) | 77.1(17.2) | NA | 13.7(600) | 10.7(470) | 34.1(1497) | 2.5(108) |
|  | no HTN | 2823/2823/NA | 59.0(1666) | 69.0(8.9) | 78.9(16.6) | NA | 10.8(306) | 7.9(222) | - | 1.6(46) |
|  | HTN | 1497/1497/NA | 65.9(986) | 71.6(8.7) | 74.1(17.8) | NA | 18.5(277) | 15.6(233) | 100(1497) | 3.9(59) |
|  | no DM | 3896/3896/NA | 61.2(2386) | 69.5(8.9) | 77.8(16.8) | NA | 12.3(478) | - | 32.8(1258) | 2.0(77) |
|  | DM | 470/470/NA | 62.6(294) | 73.8(9.0) | 71.6(19.8) | NA | 25.5(120) | 100(470) | 51.2(233) | 6.6(31) |
|  | older age | 2903/2903/NA | 62.4(1811) | 74.8(7.0) | 72.9(16.4) | NA | 19.1(554) | 13.5(390) | 38.7(1101) | 3.6(103) |
|  | younger age | 1487/1487/NA | 59.5(885) | 60.6(2.8) | 85.3(15.7) | NA | 3.1(46) | 5.4(80) | 26.8(396) | 0.3(5) |
|  | women | 2696/2696/NA | 100(2696) | 69.9(9.5) | 76.1(17.5) | NA | 15.6(421) | 110(294) | 37.2(986) | 2.7(74) |
|  | men | 1694/1694/NA | - | 68.9(8.1) | 78.6(16.7) | NA | 10.6(179) | 10.4(176) | 30.6(511) | 2.0(34) |
| **Rotterdam Study – II** | all | 1863/1863/NA | 54.5(1015) | 64.8(8.0) | 81.3(17.2) | NA | 9.1(169) | 11.1(207) | 28.4(530) | 1.2(22) |
|  | no HTN | 1333/1333/NA | 54.5(727) | 64.0(7.6) | 83.1(16.5) | NA | 5.6(75) | 8.0(106) | - | 0.6(8) |
|  | HTN | 530/530/NA | 54.4(288) | 66.9(8.8) | 76.8(18.0) | NA | 17.7(94) | 19.1(101) | 100(530) | 2.6(14) |
|  | no DM | 1565/1565/NA | 55.9(925) | 64.6(7.9) | 81.1(16.8) | NA | 9.1(150) | - | 25.9(429) | 1.1(18) |
|  | DM | 207/207/NA | 43.5(90) | 66.4(8.6) | 83.2(20.1) | NA | 9.2(19) | 100(207) | 48.8(101) | 1.9(4) |
|  | older age | 627/627/NA | 54.2(340) | 74.4(6.4) | 72.1(15.2) | NA | 20.4(128) | 14.5(91) | 38.6(242) | 2.9(18) |
|  | younger age | 1236/1236/NA | 54.6(675) | 59.9(2.5) | 86.0(16.2) | NA | 3.3(41) | 9.4(116) | 23.3(288) | 0.3(4) |
|  | women | 1015/1015/NA | 100(1015) | 65.1(8.3) | 79.5(16.4) | NA | 10.1(102) | 8.9(90) | 28.4(288) | 0.9(9) |
|  | men | 848/848/NA | - | 64.5(7.6) | 83.5(17.9) | NA | 7.9(67) | 13.8(117) | 28.5(242) | 1.5(13) |
| **SHIP** | all | 3228/3228/3228 | 51.7(1670) | 54.5(15.3) | 90.4(23.6) | 97.1(25.3) | 7.7(248) | 11.2(362) | 51.1(1649) | 2.1(67) |
|  | no HTN | 1576/1576/1576 | 63.1(994) | 47.8(14.1) | 96.1(22.5) | 104.5(22.8) | 3.2(51) | 4.0(63) | - | 0.4(6) |
|  | HTN | 1649/1649/1649 | 40.9(674) | 60.8(13.6) | 85.1(23.3) | 89.9(25.5) | 11.9(197) | 18.1(299) | 100(1649) | 3.7(61) |
|  | no DM | 2866/2866/2866 | 52.8(1514) | 52.9(15.0) | 91.8(23.0) | 98.9(24.4) | 6.0(171) | - | 47.1(1350) | 1.3(37) |
|  | DM | 362/362/362 | 43.1(156) | 67.0(11.3) | 79.5(25.0) | 82.3(27.7) | 21.3(77) | 100(362) | 82.6(299) | 8.3(30) |
|  | older age | 868/868/868 | 46.0(399) | 73.6(5.5) | 75.9(21.3) | 76.7(21.8) | 21.2(184) | 22.9(199) | 75.5(655) | 6.5(56) |
|  | younger age | 2360/2360/2360 | 53.9(1271) | 47.4(11.1) | 95.8(22.0) | 104.5(22.2) | 2.7(64) | 6.9(163) | 42.1(994) | 0.5(11) |
|  | women | 1670/1670/1670 | 100(1670) | 53.4(15.0) | 89.8(24.2) | 100.9(25.8) | 8.7(145) | 9.3(156) | 40.4(674) | 1.7(28) |
|  | men | 1558/1558/1558 | - | 55.6(15.5) | 91.1(22.8) | 93.0(24.1) | 6.6(103) | 13.2(206) | 62.6(975) | 2.5(39) |
| **Sorbs** | all | 856/856/NA | 58.5(501) | 48.8(15.7) | 92.2(19.0) | NA | 4.1(35) | NA | NA | NA |
|  | no HTN | 401/401/NA | 67.1(269) | 40.5(13.3) | 98.5(18.2) | NA | 1.5(6) | NA | NA | NA |
|  | HTN | 455/455/NA | 51.0(232) | 56.2(14.0) | 86.6(17.9) | NA | 6.4(29) | NA | NA | NA |
|  | no DM | 776/776/NA | 59.1(459) | 47.4(15.3) | 93.2(18.6) | NA | 3.2(25) | NA | NA | NA |
|  | DM | 80/80/NA | 52.5(42) | 63.0(12.1) | 82.6(20.3) | NA | 12.5(10) | NA | NA | NA |
|  | older age | 166/166/NA | 56.0(93) | 71.4(4.5) | 73.5(14.2) | NA | 16.3(27) | NA | NA | NA |
|  | younger age | 690/690/NA | 59.1(408) | 43.4(12.2) | 96.7(17.2) | NA | 1.2(8) | NA | NA | NA |
|  | women | 501/501/NA | 100(501) | 48.9(15.3) | 92.2(19.9) | NA | 4.2(21) | NA | NA | NA |
|  | men | 355/355/NA | - | 48.8(16.2) | 92.2(17.6) | NA | 3.9(14) | NA | NA | NA |
| **Vis** | all | 768/768/NA | 58.6(450) | 56.9(15.2) | 88.2(22.1) | NA | 6.9(53) | 12.0(91) | 52.2(396) | 1.4(11) |
|  | no HTN | 362/362/NA | 60.8(220) | 49.3(14.6) | 94.8(21.2) | NA | 1.4(5) | 6.4(23) | - | 0.0(0) |
|  | HTN | 396/396/NA | 56.3(223) | 64.0(11.9) | 82.1(21.3) | NA | 12.1(48) | 17.0(67) | 100(396) | 2.8(11) |
|  | no DM | 670/670/NA | 59.7(400) | 55.8(15.2) | 89.1(21.9) | NA | 6.1(41) | - | 49.2(328) | 1.2(8) |
|  | DM | 91/91/NA | 49.5(45) | 65.7(12.0) | 82.2(22.7) | NA | 13.2(12) | 100(91) | 74.4(67) | 3.3(3) |
|  | older age | 268/268/NA | 62.3(167) | 73.0(5.3) | 75.6(18.2) | NA | 16.4(44) | 19.9(53) | 77.1(205) | 3.0(8) |
|  | younger age | 500/500/NA | 56.6(283) | 48.2(11.2) | 94.9(21.0) | NA | 1.8(9) | 7.7(38) | 38.8(191) | 0.6(3) |
|  | women | 450/450/NA | 100(450) | 57.3(15.5) | 87.3(23.0) | NA | 7.3(33) | 10.1(45) | 50.3(223) | 1.1(5) |
|  | men | 318/318/NA | - | 56.2(14.8) | 89.4(20.7) | NA | 6.3(20) | 14.6(46) | 54.9(173) | 1.9(6) |
| **Women’s Genome Health Study (WGHS)** | all | 21940/21940/NA | 100(21940) | 55(7.1) | 90(22.5) | NA | 6.1(1329) | 2.5(554) | 24.5(5374) | 0.9(188) |
|  | no HTN | 16560/16560/NA | 100(16560) | 54(6.7) | 91(22.1) | NA | 5.4(887) | 1.3(221) | - | 0.7(116) |
|  | HTN | 5374/5374/NA | 100(5374) | 57(7.8) | 88(23.5) | NA | 8.2(442) | 6.2(333) | 100(5374) | 1.3(72) |
|  | no DM | 21386/21386/NA | 100(21386) | 55(7.1) | 90(22.5) | NA | 6.0(1285) | - | 23.6(5041) | 0.8(181) |
|  | DM | 554/554/NA | 100(554) | 57(7.4) | 90(24.0) | NA | 7.9(44) | 100(554) | 60.1(333) | 1.3(7) |
|  | older age | 2352/2352/NA | 100(2352) | 69(3.5) | 76(19.0) | NA | 15.3(359) | 4.3(101) | 42.6 (1003) | 2.3(55) |
|  | younger age | 16560/16560/NA | 100(19588) | 54(6.7) | 91(22.1) | NA | 5.4(887) | 1.3(221) | 0.0(0) | 0.7(116) |
|  | women | 21940/21940/NA | 100(21940) | 55(7.1) | 90(22.5) | NA | 6.1(1329) | 2.5(554) | 24.5(5374) | 0.9(188) |
|  | men | 0/0/NA | NA | NA | NA | NA | NA | NA | NA | NA |

**Abbreviations:** DM = diabetes mellitus; HTN = hypertension
