# Supplementary material for: Genome-Wide Association and Functional Follow-Up Reveals New Loci for Kidney Function
Source: PLoS Genet. 2012 Mar 29;8(3):e1002584. doi: 10.1371/journal.pgen.1002584 (PMC3315455; doi:10.1371/journal.pgen.1002584)
Supplement: Table S4 — Study-specific genotyping information for stage 1 discovery studies. (DOC) [file pgen.1002584.s016.doc]

**Table S4. Study-specific genotyping information for stage 1 discovery studies.**

| **Study** | **Array type** | **Genotype calling** | **Quality control filters for genotyped SNPs used for imputation** | **No. of SNPs used for imputation** | **Imputation** | **Imputation backbone for phased CEU haplotypes (NCBI build)** | **Filtering of imputed genotypes^1^** | **Data management and statistical analysis** | **Population stratification or principal components (PCs)** |
| --- | --- | --- | --- | --- | --- | --- | --- | --- | --- |
| **AGES** | Illumina Hu370CNV | Illumina | call rate<97%; MAF<1%; pHWE<1e-6  mishap p<1e-9,  SNPs not in Hapmap or strandedness issues merging with Hapmap | 329,804 | MACH version 1.0.16 | HapMap release 22 (build 36) | none | R, ProbABEL, Linear and Logistic Regression | We observed no association for the tested traits with the 10 PCs estimated using Eigenstrat.^[1]^ |
| **Amish Studies** | Affymetrix 500K | BRLMM | call rate<95%  MAF<1%,  pHWE<10E-6  SNPs not in Hapmap | 338,598 | MACH version 1.0.15 | phased CEU haplotypes, HapMap release 22 (build 36) | none | Measured genotype accounting for polygenic component | NA |
| **ARIC** | Affymetrix 6.0 | Birdseed | call rate <95%  MAF<1%  pHWE <10E-5 | 669,450 | MACH version 1.0.16 | HapMap release 22 (build 36) | none | ProbABEL, PLINK, R | Significant association was observed between some of the top 10 PCs estimated using Eigenstrat. The appropriate PCs were therefore included in the respective stratum as covariates in the association analyses for eGFRcys, eGFRcrea and CKD. |
| **ASPS** | Illumina Human610-Quad BeadChip | Illumina | call rate<98%  MAF<0.01  pHWE<1e-6  mishap p<1e-9 Mendelian errors>100, SNPs not in Hapmap or strandedness issues merging with Hapmap | 550,635 | MACH version 1.0.15 | HapMap release 22 (build 36) | none | R, linear and logistic fixed effects model | NA |
| **Baltimore Longitudinal Study of Aging (BLSA)** | Illumina Infinium HumanHap 550K | Beadstudio | call rate <99%  MAF <1%  pHWE <10E-4 | 501,764 | MACH version 1.0.15 | Phased CEU haplotyped, HapMap release 21 (build 35) | MAF<1%, Rsq<0.3 | SAS, MERLIN, R | Include top 2 PCs as covariates in model |
| **Cardio-vascular Health Study (CHS)** | Illumina 370CNV | Illumina BeadStudio | call rate<97%, MAF<1%  pHWE<10E-5  heterozygotes=0; SNPs not in HapMap | 306,655 | BimBam version 0.99 | HapMap CEU release 22 (build 36) | dosage variance < 0.01 | Linear and logistic regression using R, robust SE estimation | Study sites (clinic sites) were included as covariates in the regression to account for population stratification. |
| **ERF** | Illumina 6K/318K/380K, Affymetrix 250K | BeadStudio (Affymetrix) | call rate<98%, MAF<1%  pHWE<1E-6  gender mismatch, excess of heterozygosity | 487,573 | MACH version 1.0.15 | HapMap release 22 (build 36) | none | R, GenABEL, ProbABEL (linear mixed effect models) | None (single large family) |
| **Family Heart Study (FamHS)** | Illumina550K | Illumina | MAF<1%, pHWE<10E-6 | 456,293 | MACH version 1.0.15 | phased CEU haplotypes, HapMap release 22 (build 26) | none | SAS | NA |
| **Framingham Heart Study (FHS)** | Affymetrix 500K  Affymetrix 50K supplemental | Affymetrix | call rate<97%  pHWE<1e-6  MAF<1%  mishap p<1e-9; Mendelian errors>100;  SNPs not in Hapmap or strandedness issues merging with Hapmap | 378,163 | MACH version 1.0.15 | HapMap release 22 (build 36) | none | R, linear mixed effect models and GEE models, robust variance option to account for relatedness | We observed no association with CKD with the 10 PCs estimated using Eigenstrat.^[1]^ Significant association between eGFR and the 10 PCs was observed therefore, PCs were included in the analysis for association between genotype and eGFR. |
| **GENOA** | Affymetrix 6.0 (primary), Illumina 610-Quad, Illumina 660-Quad, Illumina 1M-Duo | Birdseed (Affymetrix data), Genome Studio (Illumina data) | Call Rate<95%,  MAF<1%;  pHWE < 0.001 | 1,233,495 (because of the different platforms, some SNPs may have had many missings) | MACH version 1.0.16 | HapMap release 22 (build 36), CEU founders | none | R, multic and GEE | Using the PLINK --cluster option, we did not observe any population stratification (all subjects placed within the same cluster). We did not adjust for PCs in the association analysis. |
| **Health ABC** | Illumina 1M | BeadStudio v3.3.7 | MAF<1%  call rate<97%  pHWE<10^-6^ | 914,263 | MACH version 1.0.16 | HapMap CEPH release 22 (build 36) | none | R, linear and logistic regression models | Adjust for the 1st PC |
| **Health Professionals Follow-Up Study (HPFS)** | Affymetrix Genome-Wide Human 6.0 array | Birdseed | call rate<97%, MAF<2%  pHWE<10E-4,  >1 discordance / 29 replicates; significant plate associations | 607,569 (autosomal) | MACH version 1.0.15 | phased CEU haplotypes, HapMap release 22 (build 36) | none | ProbABEL (R), SAS 9.0, PLINK | Population structure was investigated by PC analysis.^[2]^ The top 4 eigenvectors were included in all CKDGen analyses. |
| **KORA F3** | Affymetrix 500K | BRLMM | per-chip call rate <93%; MAF<5%; discrepancy for one of the 50 SNPs common on both chips; gender checks | 380,407 | MACH | HapMap release 22 (build 35) | none | MACH2QTL, PROBABEL, R, VISUAL BASIC | NA |
| **KORA F4** | Affymetrix 6.0 | BRLMM | per-chip call rate <93%; per SNP call rate <93%; MAF<1%; gender checks | 629,893 | MACH | HapMap release 22 (build 36) | none | MACH2QTL, PROBABEL, R, VISUAL BASIC | NA |
| **Korcula** | Illumina Infinium HumanCNV370v1 SNP bead microarrays | Beadstudio | call rate <98%  MAF<0.01  pHWE<10^-6^ | 317,896 | MACH version 1.0.16 | HapMap release 22 (build 36) | none | R, GenABEL, ProbABEL; | For the eGFR analysis, population structure was modeled by fitting a polygenic linear model, with the variance/covariance matrix was based on the genetic kinship matrix. For the CKD analysis, population structure was adjusted for by including in the logistic regression model the first 3 PCs of the genetic kinship matrix. |
| **Microisolates in South Tyrol (MICROS)** | Illumina Infinium HumanHap300 v2 SNP bead microarrays | Beadstudio | call rate <98%, MAF < 1%, pHWE<10E-6 | 292,917 | MACH version 1.0.16 | HapMap release 22 (build 36) | none | R, GenABEL, ProbABEL; | For the eGFR analysis, population structure was modeled by fitting a polygenic linear model, with the variance/covariance matrix was based on the genetic kinship matrix. For the CKD analysis, population structure was adjusted for by including in the logistic regression model the first 3 PCs of the genetic kinship matrix. |
| **Northern Sweden Population Health Study (NSPHS)** | Illumina 300K | Beadstudio | call rate≤97%  MAF≤1%;  pHWE≤1E-5; | 318,049 | MACH version 1.0.15 | HapMap release 22 (build 36) | none | R, GenABEL, ProbABEL | For the eGFR analysis, population structure was modeled by fitting a polygenic linear model, with the variance/covariance matrix was based on the genetic kinship matrix. For the CKD analysis, population structure was adjusted for by including in the logistic regression model the first 3 PCs of the genetic kinship matrix. |
| **Nurses' Health Study (NHS)** | Affymetrix Genome-Wide Human 6.0 array | Birdseed | call rate<97%;  MAF<2%  pHWE<10E-4;  >1 discordance / 12 replicates, significant plate associations | 606,626 (autosomal) | MACH version 1.0.15 | phased CEU haplotypes, HapMap release 22 (build 36) | none | ProbABEL (R), SAS 9.0, PLINK | Population structure was investigated by PC analysis.^[2]^ The top 3 eigenvectors were included in all CKDGen analyses. |
| **Orkney Complex Disease Study (ORCADES)** | Illumina 300K | Beadstudio | call rate≤98%  MAF≤1%;  pHWE≤1E-6; | 306,207 | MACH version 1.0.15 | HapMap release 22 (build 36) | none | R, GenABEL, ProbABEL; | For the eGFR analysis, population structure was modeled by fitting a polygenic linear model, with the variance/covariance matrix was based on the genetic kinship matrix. For the CKD analysis, population structure was adjusted for by including in the logistic regression model the first 3 PCs of the genetic kinship matrix. |
| **Popgen** | Affymetrix 6.0 | Birdseed v2 | sample call rate <0.90; SNP call rate <0.95;  MAF<1%  pHWE<0.0001; | 709,003 | MACH version 1.0.16 | phased CEU haplotypes, HapMap release 22 (build 36) | none | PLINK, R | NA |
| **Rotterdam Study – I** | Version 3 Illumina Infinium II HumanHap550 | BeadStudio | call rate<90%; MAF<0.01;  pHWE<1E-5;  Mendelian errors>100; SNPs not in Hapmap or strandedness issues merging with Hapmap | 491,875 | MACH | HapMap release 22 (build 36) | none | ProbABEL | NA |
| **Rotterdam Study – II** | Version 3 Illumina Infinium II HumanHap550 | BeadStudio | call rate<90% MAF<0.01;  pHWE<1E-5  Mendelian errors>100; SNPs not in Hapmap or strandedness issues merging with Hapmap | 495,478 | MACH | HapMap release 22 (build 36) | none | ProbABEL | NA |
| **SHIP** | Affymetrix 6.0 | Affymetrix Birdseed2 | none | 869,224 | IMPUTE version 0.5.0 | HapMap release 22 (build 36) | none | SNPTEST v1.1.5, QUICKTEST v0.94, R, InforSense, InterSystems Caché, SAS | We observed no population stratification using PCs estimated using Eigenstrat.^[1]^ |
| **Sorbs** | 500K Affymetrix GeneChip (250K Sty and 250K Nsp arrays, Affymetrix, Inc) and Affymetrix Genome-Wide Human SNP Array 6.0 | Microarray Core Facility of the Interdisciplinary Centre for Clinical Research, University of Leipzig, Germany and ATLAS Biolabs GmbH, Berlin, Germany | call rate<95%  MAF<1%  pHWE<10^-4^ | 378,513 | IMPUTE | HapMap CEU release 21 (build 35) | Proper-info <0.4 | ProbABEL with robust variance option to account for residual relatedness | We observed very little deviation in the distributions of 4 PCs and applied exclusions to population outlier individuals. However due to relative ethnic isolation of the Sorbs Slavonic population, we apply additional correction from genome-wide data for remaining population stratification using robust variance approach. |
| **Vis** | Illumina HumanHap300 beadchip | Beadstudio | call rate≤98%  MAF≤1%  pHWE≤1E-6 | 305,068 | MACH version 1.0.15 | HapMap release 22 (build 36) | none | R, GenABEL, ProbABEL; | For the eGFR analysis, population structure was modeled by fitting a polygenic linear model, with the variance/covariance matrix based on the genetic kinship matrix. For the CKD analysis, population structure was adjusted for by including in the logistic regression model the first 3 PCs of the genetic kinship matrix. |
| **Women’s Genome Health Study (WGHS)** | Illumina HumanHap300 Duo "plus" | Beadstudio v3.1 | call rate<98%  MAF<1%  pHWE<1E-6 | 331,993 | MACH version 1.0.15 | HapMap release 22 (build 36) | none | PLINK, R, ProbABEL | none |

^1^Rsq is the estimate of squared correlation between imputed and true genotypes provided by the imputation software MACH

References

1.     Price AL, Patterson NJ, Plenge RM, Weinblatt ME, Shadick NA, et al. (2006) Principal components analysis corrects for stratification in genome-wide association studies. Nat Genet 38(8): 904-909.

2.     Patterson N, Price AL, Reich D. (2006) Population structure and eigenanalysis. PLoS Genet 2(12): e190.
