# Supplementary material for: Genome-Wide Association and Functional Follow-Up Reveals New Loci for Kidney Function
Source: PLoS Genet. 2012 Mar 29;8(3):e1002584. doi: 10.1371/journal.pgen.1002584 (PMC3315455; doi:10.1371/journal.pgen.1002584)
Supplement: Table S5 — Characteristics of stage 2 replication studies. (DOC) [file pgen.1002584.s017.doc]

**Table S5. Characteristics of stage 2 replication studies.**

| **Study** | **Stratum (include only subjects from the specified stratum)** | **Sample Size eGFRcrea / CKD / eGFRcys** | **Women:**  **%(n)** | **Age**  **[years]**  **mean(SD)** | **eGFRcrea [ml/min/ 1.73 m^2^]**  **mean(SD)** | **eGFRcys [ml/min/ 1.73 m^2^]**  **mean(SD)** | **CKD**  **%(n)** | **DM**  **%(n)** | **HTN**  **%(n)** | **CKD45**  **%(n)** |
| --- | --- | --- | --- | --- | --- | --- | --- | --- | --- | --- |
| **3 City Study** | all | 5413/5413/1018 | 61.3(3319) | 74.08(5.4) | 72.9(16.5) | 91.5(25.4) | 20.3(1100) | 9.6(515) | 78.5(4250) | 4.3(195) |
|  | no HTN | 1163/1163/215 | 70.3(818) | 72.3(4.8) | 75.8(15.3) | 94.3(20.0) | 16.7(159) | 4.4(51) | - | 1.3(13) |
|  | HTN | 4250/4250/803 | 58.9(2501) | 74.6(5.5) | 72.1(16.7) | 90.7(26.7) | 22.1(941) | 11.0(464) | 100(4250) | 5.2(182) |
|  | no DM | 4872/4872/897 | 62.6(3048) | 74.1(5.4) | 72.6(16.1) | 91.5(25.8) | 20.6(1001) | - | 77.3(3765) | 4.2(171) |
|  | DM | 515/515/118 | 49.1(253) | 74.2(5.3) | 76.0(19.4) | 91.7(23.0) | 18.6(96) | 100(515) | 90.1(464) | 5.2(23) |
|  | older age | 5413/5413/1018 | 61.3(3319) | 74.08(5.4) | 72.9(16.5) | 91.5(25.4) | 20.3(1100) | 9.6(515) | 78.5(4250) | 4.3(195) |
|  | younger age | 0/0/NA | NA | NA | NA | NA | NA | NA | NA | NA |
|  | women | 3319/3319/601 | 100(3319) | 74.2(5.4) | 72.0(15.9) | 91.9(23.1) | 21.0(698) | 7.7(253) | 75.4(2501) | 4.2(115) |
|  | men | 2094/2094/417 | - | 73.9(5.4) | 74.4(17.3) | 90.9(28.5) | 19.2(402) | 12.6(262) | 83.5(1749) | 4.5(80) |
| **Blue Mountains Eye Study (BMES)** | all | 2424/2424/NA | 56.8(1376) | 69.4(9.5) | 78.7(20.2) | NA | 13.2(320) | 10.9(264) | 76.4(1852) | 3.3(71) |
|  | no HTN | 571/571/NA | 55.2(315) | 66.4(9.8) | 81.6(19.8) | NA | 8.9(51) | 5.6(32) | - | 1.7(9) |
|  | HTN | 1852/1852/NA | 57.2(1060) | 70.4(9.2) | 77.8(20.2) | NA | 14.5(269) | 12.5(232) | 100(1852) | 3.8(62) |
|  | no DM | 2158/2158/NA | 57.9(1249) | 69.2(9.6) | 79.0(20.1) | NA | 12.6(271) | - | 75.0(1619) | 3.0(59) |
|  | DM | 264/264/NA | 47.7(126) | 71.1(8.2) | 76.0(20.7) | NA | 18.6(49) | 100(264) | 87.9(232) | 5.3(12) |
|  | older age | 1580/1580/NA | 56.6(895) | 75.0(6.1) | 73.4(18.2) | NA | 18.3(289) | 12.3(195) | 81.3(1285) | 5.0(68) |
|  | younger age | 844/844/NA | 57.0(481) | 59.0(4.7) | 88.5(20.0) | NA | 3.7(31) | 8.2(69) | 67.2(567) | 0.4(3) |
|  | women | 1376/1376/NA | 100(1376) | 69.4(9.7) | 77.7(20.3) | NA | 14.7(202) | 9.2(126) | 77.0(1060) | 3.4(41) |
|  | men | 1048/1048/NA | - | 69.4(9.2) | 79.9(20.0) | NA | 11.3(118) | 13.2(138) | 75.6(792) | 3.1(30) |
| **CoLaus** | all | 5409/5409/NA | 52.9(2863) | 53.4(10.7) | 90.2(19.5) | NA | 3.8(208) | 9.6(352) | 36.2(1957) | 0.5(25) |
|  | no HTN | 3448/3448/NA | 57.7(1989) | 50.1(9.9) | 92.7(18.9) | NA | 2.1(72) | 4.2(97) | - | 0.1(5) |
|  | HTN | 1957/1957/NA | 44.5(870) | 59.2(9.6) | 85.8(19.8) | NA | 6.9(135) | 18.7(255) | 100(1957) | 1.1(20) |
|  | no DM | 3296/3296/NA | 53.5(1763) | 52.6(10.6) | 89.6(18.9) | NA | 3.7(122) | - | 33.7(1109) | 0.5(16) |
|  | DM | 352/352/NA | 32.4(114) | 60.3(8.7) | 86.8(21.3) | NA | 9.1(32) | 100(352) | 72.4(255) | 0.9(3) |
|  | older age | 866/866/NA | 54.5(472) | 70.0(3.1) | 76.1(16.5) | NA | 14.7(127) | 18.7(106) | 65.6(567) | 2.0(15) |
|  | younger age | 4543/4543/NA | 52.6(2391) | 50.3(8.6) | 92.9(18.9) | NA | 1.8(81) | 8.0(246) | 30.6(1390) | 0.2(10) |
|  | women | 2863/2863/NA | 100(2863) | 53.88(10.72) | 88.7(20.1) | NA | 4.9(141) | 6.1(114) | 30.4(870) | 0.5(13) |
|  | men | 2546/2546/NA | - | 52.93(10.76) | 91.9(18.7) | NA | 2.6(67) | 13.4(238) | 42.7(1087) | 0.5(12) |
| **Cardiovascular risk in Young Finns Study (YFS)** | all | 2023/2023/NA | 54.7(1107) | 37.7(5.0) | 100.3(15.8) | NA | 0.2(5) | 2.1(42) | 20.0(404) | 0.0(1) |
|  | no HTN | 1619/1619/NA | 58.1(679) | 37.3(5.0) | 100.6(15.8) | NA | 0.1(2) | 0.9(15) | - | 0.0(0) |
|  | HTN | 404/404/NA | 41.3(167) | 39.4(4.7) | 99.3(16.0) | NA | 0.7(3) | 6.7(27) | 100(404) | 0.2(1) |
|  | no DM | 1981/1981/NA | 55.1(1091) | 37.7(5.0) | 100.3(15.7) | NA | 0.2(4) | - | 19.0(377) | 0.1(1) |
|  | DM | 42/42/NA | 38.1(16) | 39.5(5.4) | 104.0(20.1) | NA | 2.4(1) | 100(42) | 64.3(27) | 0.0(0) |
|  | older age | 0/0/NA | NA | NA | NA | NA | NA | NA | NA | NA |
|  | younger age | 2023/2023/NA | 54.7(1107) | 37.7(5.0) | 100.3(15.8) | NA | 0.2(5) | 2.1(42) | 20.0(404) | 0.0(1) |
|  | women | 1107/1107/NA | 100(1107) | 37.7(5.0) | 100.6(17.3) | NA | 0.3(3) | 1.5(16) | 15.1(167) | 0.0(0) |
|  | men | 916/916/NA | - | 37.7(5.0) | 100.1(13.8) | NA | 0.2(2) | 2.8(26) | 25.9(237) | 0.1(1) |
| **Estonian Genome Center** | all | 893/893/NA | 49.2(439) | 37.9(15.8) | 101.1(20.4) | NA | 1.6(14) | 1.8(16) | 22.6(202) | 0.3(3) |
|  | no HTN | 691/691/NA | 51.1(353) | 34.2(13.3) | 104.1(19.2) | NA | 0.6(4) | 2.0(14) | 0 | NA |
|  | HTN | 202/202/NA | 42.8(86) | 50.4(17.2) | 90.8(20.8) | NA | 5.0(10) | 1.0(2) | 100(202) | NA |
|  | no DM | 877/877/NA | 49.3(432) | 37.9(15.8) | 101.3(20.3) | NA | 1.6(14) | - | 22.8(200) | NA |
|  | DM | 16/16/NA | 43.8(7) | 37.8(16.0) | 103.8(20.7) | NA | - | 100(16) | 12.5(2) | NA |
|  | older age | 72/72/NA | 48.6(35) | 72.5(5.9) | 76.3(19.9) | NA | 16.7(12) | 2.8(2) | 65.3(47) | NA |
|  | younger age | 821/821/NA | 49.2(404) | 34.8(12.3) | 103.3(19.6) | NA | 0.2(2) | 1.7(14) | 18.9(155) | NA |
|  | women | 439/439/NA | 100(439) | 38.8(16.1) | 100.3(21.1) | NA | 2.3(10) | 1.6(7) | 19.6(86) | NA |
|  | men | 454/454/NA | - | 37.0(15.4) | 101.9(19.6) | NA | 0.9(4) | 2.0(9) | 25.6(116) | NA |
| **Family Heart Study - II (FamHS-II)** | all | 1426/1426/NA | 56.9(812) | 47.8(13.2) | 93.7(19.7) | NA | 2.2(31) | 4.2(59) | 18.0(256) | 0.4(5) |
|  | no HTN | 1170/1170/NA | 57.8(676) | 45.1(12.3) | 95.6(19.2) | NA | 1.5(17) | 2.6(30) | - | 0.3(3) |
|  | HTN | 256/256/NA | 53.1(136) | 60.2(9.7) | 85.1(20.2) | NA | 5.5(14) | 11.4(29) | 100(256) | 0.8(2) |
|  | no DM | 1358/1358/NA | 57.3(778) | 47.3(13.1) | 93.9(19.6) | NA | 2.1(28) | - | 16.6(225) | 0.3(4) |
|  | DM | 59/59/NA | 47.5(28) | 58.4(11.8) | 89.4(22.9) | NA | 5.1(3) | 100(59) | 49.2(29) | 1.7(1) |
|  | older age | 185/185/NA | 55.1(102) | 69.0(3.6) | 76.5(16.0) | NA | 10.8(20) | 10.9(20) | 49.7(92) | 1.6(3) |
|  | younger age | 1241/1241/NA | 57.2(710) | 44.6(11.0) | 96.3(19.0) | NA | 0.9(11) | 3.2(39) | 13.2(164) | 0.2(2) |
|  | women | 812/812/NA | 100(812) | 47.7(12.9) | 93.3(20.4) | NA | 2.8(23) | 3.5(28) | 16.8(136) | 0.5(4) |
|  | men | 614/614/NA | - | 47.9(13.6) | 94.2(18.9) | NA | 1.3(8) | 5.1(31) | 19.5(120) | 0.2(1) |
| **GoDARTs** | all | 2942/2893/NA | 46.4(1368) | 66.2(10.7) | 65.5(20.5) | NA | 14.5(427) | 100(2942) | 46.6(1368) | 18.4(542) |
|  | no HTN | 1400/1370/NA | 49.2(689) | 65.2(10.8) | 66.2(21.2) | NA | 15.2(214) | 100(1400) | NA | 19.9(279) |
|  | HTN | 1542/1523/NA | 46.0(710) | 67.3(10.4) | 54.9(19.9) | NA | 13.8(213) | 100(1542) | NA | 17.0(263) |
|  | no DM | 0/0/NA | NA | NA | NA | NA | NA | - | NA | NA |
|  | DM | 2942/2893/NA | 46.4(1368) | 66.2(10.7) | 65.5(20.5) | NA | 14.5(427) | 100(2942) | 46.6(1370) | 18.4(542) |
|  | older age | 1753/1734/NA | 49.9(876) | 70.68(10.0) | 61.9(19.6) | NA | 17.6(309) | 100(1753) | 55.3(970) | 21.8(383) |
|  | younger age | 1189/1342/NA | 43.9(523) | 59.8(7.9) | 70.9(20.7) | NA | 24.1(287) | 100(1189) | 48.1(572) | 13.3(159) |
|  | women | 1368/1342/NA | 100(136) | 65.4(15.4) | 60.05(20.5) | NA | 20.9(287) | 100(1368) | 51.9(710) | 25.5(350) |
|  | men | 1574/1551/NA | NA | 63.02(17.0) | 70.3(19.3) | NA | 8.8(140) | 100(1574) | 52.9(832) | 12.1(192) |
| **INGI – Carlantino Project** | all | 447/447/NA | 60.8(272) | 50.4(16.2) | 93.9(22.4) | NA | 0.0(0) | 9.4(42) | 34.9(156) | 0.0(0) |
|  | no HTN | 291/291/NA | 58.8(171) | 45.5(16.4) | 98.0(22.2) | NA | 0.0(0) | 6.2(18) | - | 0.0(0) |
|  | HTN | 156/156/NA | 64.7(101) | 59.5(11.2) | 86.12(20.6) | NA | 0.0(0) | 15.4(24) | 100(156) | 0.0(0) |
|  | no DM | 405/405/NA | 61.2(248) | 49.2(16.4) | 94.3(22.3) | NA | 0.0(0) | - | 32.6(132) | 0.0(0) |
|  | DM | 42/42/NA | 57.1(24) | 61.5(8.8) | 90.5(23.2) | NA | 0.0(0) | 100(42) | 57.1(24) | 0.0(0) |
|  | older age | 83/83/NA | 62.6(52) | 71.4(4.6) | 74.0(16.2) | NA | 0.0(0) | 16.9(14) | 59.0(49) | 0.0(0) |
|  | younger age | 364/364/NA | 60.4(220) | 45.6(13.9) | 98.5(21.1) | NA | 0.0(0) | 7.7(28) | 29.4(107) | 0.0(0) |
|  | women | 272/272/NA | 100(272) | 50.3(16.2) | 95.1(24.3) | NA | 0.0(0) | 8.8(24) | 37.1(101) | 0.0(0) |
|  | men | 175/175/NA | - | 50.6(16.2) | 92.0(18.8) | NA | 0.0(0) | 10.3(18) | 31.4(55) | 0.0(0) |
| **INGI – Cilento Study** | all | 817/817/NA | 55.2(451) | 54.2(18.1) | 88.7(21.8) | NA | 7.9(65) | 10.4(85) | 38.5(315) | 1.7(14) |
|  | no HTN | 340/340/NA | 53.2(181) | 44.0(15.2) | 96.1(19.3) | NA | 0.003(1) | 3.8(13) | - | 0.0(0) |
|  | HTN | 315/315/NA | 58.1(183) | 65.4(12.5) | 79.9(20.7) | NA | 16.2(51) | 18.7(59) | 100(315) | 3.2(10) |
|  | no DM | 605/605/NA | 56.0(339) | 52.7(17.7) | 89.7(21.3) | NA | 6.8(41) | - | 42.3(256) | 1.0(6) |
|  | DM | 85/85/NA | 55.3(47) | 66.0(13.3) | 79.3(23.3) | NA | 15.3(13) | 100(85) | 69.4(59) | 5.9(5) |
|  | older age | 267/267/NA | 56.9(152) | 74.9(6.2) | 72.6(18.0) | NA | 23.6(63) | 19.1(51) | 66.3(177) | 5.2(14) |
|  | younger age | 550/550/NA | 54.3(299) | 44.1(12.6) | 96.5(19.1) | NA | 0.004(2) | 6.2(34) | 25.1(138) | 0.0(0) |
|  | women | 451/451/NA | 100(451) | 54.1(18.3) | 88.3(22.2) | NA | 8.9(40) | 10.4(47) | 40.6(183) | 2.2(10) |
|  | men | 366/366/NA | - | 54.3(18.0) | 89.1(21.4) | NA | 6.8(25) | 10.4(38) | 36.1(132) | 1.1(4) |
| **INGI – FVG Project** | all | 874/874/NA | 59.4(519) | 52.4(16.5) | 90.6(21.8) | NA | 6.0(52) | 6.7(59) | 48.8(427) | 0.0(0) |
|  | no HTN | 447/447/NA | 55.7(289) | 44.8(14.8) | 96.2(21.0) | NA | 2.5(11) | 3.3(15) | - | 0.0(0) |
|  | HTN | 427/427/NA | 44.3(230) | 60.3(14.4) | 84.7(21.0) | NA | 9.6(41) | 10.3(44) | 100(427) | 0.0(0) |
|  | no DM | 815/815/NA | 96.0(498) | 51.6(16.5) | 91.2(21.4) | NA | 5.3(43) | - | 47.0(383) | 0.0(0) |
|  | DM | 59/59/NA | 4.0(21) | 63.0(12.8) | 82.0(24.9) | NA | 15.2(9) | 100(59) | 74.6(44) | 0.0(0) |
|  | older age | 212/212/NA | 26.2(136) | 74.2(5.7) | 73.9(17.9) | NA | 18.9(40) | 12.7(27) | 81.1(172) | 0.0(0) |
|  | younger age | 662/662/NA | 73.8(383) | 45.4(12.2) | 95.9(20.2) | NA | 1.8(12) | 4.8(32) | 38.5(255) | 0.0(0) |
|  | women | 519/519/NA | 100(519) | 52.7(17) | 90.1(22.6) | NA | 6.4(33) | 4.0(21) | 44.3(230) | 0.0(0) |
|  | men | 355/355/NA | - | 51.8(15.9) | 91.2(20.6) | NA | 5.3(19) | 10.7(38) | 55.5(197) | 0.0(0) |
| **INGI – Val Borbera Study** | all | 1636/1636/NA | 55.8(913) | 55.3(17.9) | 89.2(23.3) | NA | 8.5(139) | 6.5(107) | 43.8(717) | 1.9(32) |
|  | no HTN | 917/917/NA | 58.9(541) | 45.9(15.6) | 96.0(21.5) | NA | 3.2(29) | 2.9(27) | - | 0.5(5) |
|  | HTN | 717/717/NA | 51.7(371) | 67.2(12.8) | 80.5(22.6) | NA | 15.3(110) | 11.0(79) | 100(110) | 3.7(27) |
|  | no DM | 1529/1529/NA | 56.1(858) | 54.4(17.9) | 89.6(23.2) | NA | 8.0(123) | - | 41.7(638) | 1.9(30) |
|  | DM | 107/107/NA | 51.4(55) | 67.6(11.5) | 83.5(23.4) | NA | 14.9(16) | 100(107) | 73.8(79) | 1.8(2) |
|  | older age | 533/533/NA | 57.9(309) | 75.3(6.9) | 73.9(19.4) | NA | 20.6(110) | 12.0(64) | 78.2(417) | 5.8(31) |
|  | younger age | 1103/1103/NA | 54.7(604) | 45.6(12.7) | 96.6(21.3) | NA | 2.6(29) | 3.9(43) | 27.2(300) | 0.1(1) |
|  | women | 913/913/NA | 100(913) | 55.4(18.2) | 88.4(24.0) | NA | 9.3(85) | 6.0(55) | 40.6(371) | 1.8(17) |
|  | men | 723/723/NA | - | 55.0(17.5) | 90.2(22.2) | NA | 7.5(54) | 7.2(52) | 47.8(346) | 2.0(15) |
| **JUPITER** | all | 8780/8780/NA | 32.2(2826) | 66.1(7.8) | 80.1(18.1) | NA | 11.5(1008) | 0.6(54) | 63.8(5602) | 1.5(117) |
|  | no HTN | 3173/3173/NA | 29.2(925) | 64.8(7.7) | 81.8(17.2) | NA | 8.0(255) | 0.5(16) | - | 0.6(19) |
|  | HTN | 5602/5602/NA | 33.9(1899) | 66.8(7.7) | 79.1(18.6) | NA | 13.4(753) | 0.7(38) | 100(5602) | 2.0(98) |
|  | no DM | 8708/8708/NA | 32.2(2804) | 66.1(7.8) | 80.1(18.1) | NA | 11.5(998) | - | 63.8(5554) | 1.5(114) |
|  | DM | 54/54/NA | 29.6(16) | 66.9(7.9) | 73.2(15.2) | NA | 16.7(9) | 100(54) | 70.4(38) | 6.2(3) |
|  | older age | 4548/4548/NA | 46.7(2123) | 72.1(5.1) | 73.7(16.2) | NA | 18.3(834) | 0.7(31) | 68.8(3128) | 2.8(106) |
|  | younger age | 4232/4232/NA | 16.6(703) | 59.6(4) | 87.0(17.6) | NA | 4.1(174) | 0.5(23) | 58.5(2474) | 0.3(11) |
|  | women | 2826/2826/NA | 100(2826) | 69.8(6) | 74.7(16.9) | NA | 18.8(532) | 0.6(16) | 67.2(1899) | 2.6(61) |
|  | men | 5954/5954/NA | - | 64.3(7.9) | 82.7(18.1) | NA | 8.0(476) | 0.6(38) | 62.2(3703) | 1.0(56) |
| **KORA F3 – NGWA** | all | 1498/1498/1493 | 52.5(785) | 51.6(13.3) | 92.6(21.3) | 123.5(29.5) | 5.5(82) | 5.1(76) | 29.4(437) | 1.6(23) |
|  | no HTN | 1051/1051/NA | 56.6(594) | 49.5(12.4) | 94.0(20.1) | 126.6(28.2) | 3.2(34) | 3.2(34) | - | 0.8(9) |
|  | HTN | 439/439/NA | 42.6(186) | 56.6(13.9) | 89.3(23.6) | 116.1(31.2) | 11.0(48) | 9.6(42) | 100(437) | 3.4(14) |
|  | no DM | 1416/1416/NA | 52.7(744) | 50.9(12.9) | 93.2(21.0) | 124.8(28.9) | 5.0(70) | - | 28.0(395) | 1.4(20) |
|  | DM | 76/76/NA | 50.0(38) | 64.9(13.0) | 82.2(24.2) | 99.4(30.8) | 15.7(12) | 100(76) | 55.3(42) | 4.4(3) |
|  | older age | 257/257/NA | 51.2(131) | 76.3(5.8) | 74.7(22.3) | 87.5(25.6) | 24.2(62) | 15.2(39) | 47.1(120) | 8.0(17) |
|  | younger age | 1241/1241/NA | 52.8(654) | 46.5(7.3) | 96.3(19.1) | 131(24.3) | 1.7(20) | 3.0(37) | 25.8(317) | 0.5(6) |
|  | women | 787/787/NA | 100(785) | 51.6(13.0) | 91.4(21.1) | 128.7(30.4) | 5.9(46) | 4.9(38) | 23.8(186) | 1.5(11) |
|  | men | 711/711/NA | - | 51.5(13.6) | 94.0(21.4) | 117.8(27.3) | 5.1(36) | 5.3(38) | 35.6(251) | 1.8(12) |
| **KORA F4 - NGWA** | all | 1201/1201/1198 | 52.4(629) | 49.2(15.4) | 92.6(22.4) | 118.5(27.5) | 5.8(70) | 4.0(48) | 13.3(159) | 1.2(14) |
|  | no HTN | 1040/1040/NA | 54.5(566) | 47.8(14.7) | 94.3(22.3) | 120.4(27.4) | 4.7(49) | 3.2(33) | - | 1.2(12) |
|  | HTN | 160/160/NA | 39.0(62) | 58.3(16.9) | 81.4(19.7) | 105.8(24.6) | 13.2(21) | 9.5(15) | 100(159) | 1.4(2) |
|  | no DM | 1152/1152/NA | 53.1(611) | 48.4(14.9) | 93.3(22.0) | 119.6(27.0) | 5.0(57) | - | 12.5(143) | 1.0(11) |
|  | DM | 48/48/NA | 37.5(18) | 68.5(13.7) | 77.1(24.9) | 93.5(26.4) | 25.0(12) | 100(48) | 31.2(15) | 7.7(3) |
|  | older age | 249/249/NA | 47.2(117) | 76.5(4.2) | 71.4(16.5) | 87.1(19.9) | 23.8(59) | 13.4(33) | 26.7(66) | 6.5(13) |
|  | younger age | 953/953/NA | 53.8(512) | 42.1(7.0) | 98.2(20.4) | 126.6(22.9) | 1.2(11) | 15.8(15) | 9.8(93) | 0.1(1) |
|  | women | 629/629/NA | 100(628) | 48.5(14.9) | 93.1(24.1) | 123.1(28.4) | 5.3(33) | 2.9(18) | 9.9(62) | 0.1(6) |
|  | men | 573/573/NA | - | 49.9(15.9) | 92.1(20.4) | 113.4(25.5) | 6.5(37) | 5.3(30) | 17.0(97) | 1.5(8) |
| **Ogliastra Genetic Park (OGP) – (Talana excluded)** | all | 3000/3000/NA | 57.9(1737) | 63.3(12.6) | 78.7(23.3) | NA | 22.6(677) | 22.6(679) | 35.9(1078) | 3.8(113) |
|  | no HTN | 1922/1922/NA | 55.1(1060) | 60.5(12.2) | 84.6(22.4) | NA | 11.5(222) | 13.0(250) | - | 1.0(20) |
|  | HTN | 1078/1078/NA | 62.8(677) | 68.3(11.9) | 68.2(21.1) | NA | 42.2(455) | 39.8(429) | 100(1078) | 8.6(93) |
|  | no DM | 2321/2321/NA | 60.4(1402) | 62.6(12.5) | 78.5(22.9) | NA | 23.4(544) | - | 28.0(649) | 3.7(86) |
|  | DM | 679/679/NA | 49.3(335) | 65.5(12.8) | 79.3(24.4) | NA | 19.6(133) | 100(679) | 63.2(429) | 3.4(27) |
|  | older age | 1285/1285/NA | 58.4(751) | 75.5(6.7) | 67.9(19.8) | NA | 39.8(512) | 28.6(368) | 50.3(646) | 7.7(99) |
|  | younger age | 1715/1715/NA | 57.5(986) | 54.1(7.1) | 86.8(22.4) | NA | 9.6(165) | 18.1(311) | 25.2(432) | 0.8(14) |
|  | women | 1737/1737/NA | 100(1737) | 63.7(12.6) | 76.7(22.4) | NA | 24.7(429) | 19.3(335) | 39.0(677) | 3.9(68) |
|  | men | 1263/1263/NA | - | 62.8(12.7) | 81.5(24.2) | NA | 19.6(248) | 27.2(344) | 31.7(401) | 3.6(45) |
| **Ogliastra Genetic Park (OGP) - Talana** | all | 862/862/NA | 57.3(494) | 50.9(19.1) | 91.2(23.6) | NA | 7.5(65) | 5.1(44) | 37.3(322) | 1.5(13) |
|  | no HTN | 540/540/NA | 63.1(341) | 44.6(17.8) | 96.2(22.5) | NA | 3.5(19) | 2.4(13) | - | 0.4(2) |
|  | HTN | 322/322/NA | 47.5(153) | 61.5(16.4) | 82.7(23.1) | NA | 14.3(46) | 9.6(31) | 100(322) | 3.4(11) |
|  | no DM | 818/818/NA | 57.4(470) | 50.0(18.9) | 92.0(23.4) | NA | 6.3(52) | - | 35.6(291) | 1.1(9) |
|  | DM | 44/44/NA | 54.5(24) | 68.9(13.5) | 75.9(22.8) | NA | 29.5(13) | 100(44) | 70.4(31) | 9.1(4) |
|  | older age | 233/233/NA | 58.4(136) | 76.2(7.1) | 72.4(18.2) | NA | 23.2(54) | 12.0(28) | 63.5(148) | 4.7(11) |
|  | younger age | 629/629/NA | 57.0(358) | 41.6(12.5) | 98.1(21.6) | NA | 1.7(11) | 2.5(16) | 28.0(174) | 0.3(2) |
|  | women | 494/494/NA | 100(494) | 51.6(19.2) | 90.5(23.2) | NA | 7.9(39) | 4.8(24) | 31.0(153) | 1.6(8) |
|  | men | 368/368/NA | - | 50.1(19.0) | 92.1(24.2) | NA | 7.1(26) | 54.3(20) | 46.0(169) | 1.3(5) |
| **PROSPER / PHASE Study** | all | 5236/5236/NA | 51.7(2718) | 75.3(3.4) | 72.0(21.4) | NA | 29.6(1549) | 10.4(544) | 62.1(3251) | 7.6(302) |
|  | no HTN | 1985/1985/NA | 37.8(751) | 75.2(3.4) | 73.3(19.3) | NA | 25.7(510) | 12.5(249) | - | 5.5(86) |
|  | HTN | 3251/3251/NA | 60.5(1969) | 75.4(3.3) | 71.3(22.6) | NA | 32.0(1039) | 9.1(295) | 100(3251) | 8.9(216) |
|  | no DM | 4692/4692/NA | 52.8(2481) | 75.4(3.4) | 71.7(21.4) | NA | 29.9(1404) | - | 63.0(2962) | 7.8(278) |
|  | DM | 544/544/NA | 43.9(239) | 75.2(3.2) | 75.4(21.8) | NA | 26.7(145) | 100(544) | 54.2(295) | 5.7(24) |
|  | older age | 5236/5236/NA | 51.7(2718) | 75.3(3.4) | 72.0(21.4) | NA | 29.6(1549) | 10.4(544) | 62.1(3251) | 7.6(302) |
|  | younger age | 0/0/NA | NA | NA | NA | NA | NA | NA | NA | NA |
|  | women | 2718/2718/NA | 100(2718) | 75.7(3.4) | 72.5(24.2) | NA | 32.0(870) | 8.8(239) | 72.4(1969) | 9.1(186) |
|  | men | 2518/2518/NA | - | 75.0(3.3) | 71.5(18.1) | NA | 27.0(679) | 12.1(305) | 51.0(1288) | 5.9(116) |
| **SAPALDIA** | all | 6026/6026/NA | 50.2(3026) | 52.2(11.4) | 90.7(17.3) | NA | 2.9(174) | 2.9(172) | 27.5(1619) | 0.4(22) |
|  | no HTN | 4266/4266/NA | 55.2(2354) | 50.1(11.3) | 92.3(17.4) | NA | 2.3(98) | 1.8(77) | - | 0.3(13) |
|  | HTN | 1619/1619/NA | 36.0(583) | 57.5(9.7) | 86.8(16.5) | NA | 4.2(68) | 5.3(85) | 100(1399) | 0.5(8) |
|  | no DM | 5854/5854/NA | 50.8(2972) | 51.9(11.4) | 90.8(17.2) | NA | 2.7(159) | - | 26.8(1534) | 0.3(16) |
|  | DM | 172/172/NA | 31.3(54) | 60.7(8.3) | 85.5(21.1) | NA | 8.7(15) | 100(172) | 52.5(85) | 3.4(6) |
|  | older age | 931/931/NA | 53.2(495) | 68.7(2.2) | 76.4(14.7) | NA | 10.7(100) | 6.9(64) | 46.2(417) | 9.7(9) |
|  | younger age | 5095/5095/NA | 49.7(2531) | 49.2(9.7) | 93.3(16.4) | NA | 1.4(74) | 2.1(108) | 24.1(1202) | 0.3(13) |
|  | women | 3026/3026/NA | 100(3026) | 52.5(11.4) | 89.4(18.1) | NA | 3.8(116) | 1.8(54) | 19.8(583) | 0.4(13) |
|  | men | 3000/3000/NA | - | 51.8(11.4) | 92.0(16.3) | NA | 2(58) | 3.9(118) | 35.1(1036) | 0.3(9) |
| **SAPHIR** | all | 1721/1721/NA | 37.1(639) | 51.4(6.0) | 91.7(16.1) | NA | 1.1(19) | 3.3(56) | 55.7(959) | NA |
|  | no HTN | 746/746/NA | 33.6(251) | 50.0(6.0) | 93.0(15.7) | NA | 0.9(7) | 1.2(9) | - | NA |
|  | HTN | 959/959/NA | 39.8(382) | 52.5(5.7) | 90.8(16.2) | NA | 1.3(12) | 4.7(45) | 100(959) | NA |
|  | no DM | 1665/1665/NA | 37.4(623) | 51.3(6.0) | 91.5(15.8) | NA | 1.1(18) | - | 55.4(914) | NA |
|  | DM | 56/56/NA | 28.6(16) | 54.4(4.7) | 99.5(21.8) | NA | 1.8(1) | 100(56) | 83.3(54) | NA |
|  | older age | 4/4/NA | 50.0(2) | 66.5(0.6) | 75.5(13.0) | NA | 25.0(1) | 0.0(0) | 75.0(3) | NA |
|  | younger age | 1717/1717/NA | 37.1(637) | 51.4(6.0) | 91.8(16.0) | NA | 1.0(18) | 3.3(56) | 56.2(956) | NA |
|  | women | 639/639/NA | 100(639) | 55.7(4.3) | 88.6(17.4) | NA | 2.2(14) | 2.5(16) | 60.3(382) | NA |
|  | men | 1082/1082/NA | - | 48.9(5.4) | 93.6(14.9) | NA | 0.5(5) | 3.7(40) | 53.8(577) | NA |

**Abbreviations:** DM = diabetes mellitus; HTN = hypertension
