# Supplementary material for: Genome-Wide Association and Functional Follow-Up Reveals New Loci for Kidney Function
Source: PLoS Genet. 2012 Mar 29;8(3):e1002584. doi: 10.1371/journal.pgen.1002584 (PMC3315455; doi:10.1371/journal.pgen.1002584)
Supplement: Table S6 — Study-specific genotyping information for stage 2 in silico replication studies. (DOC) [file pgen.1002584.s018.doc]

**Table S6. Study-specific genotyping information for stage 2 *in silico* replication studies.**

| **Study*** | **Array type** | **Genotype calling** | **Quality control filters for genotyped SNPs used for imputation** | **No. of SNPs used for imputation** | **Imputation** | **Imputation backbone for phased CEU haplotypes (NCBI build)** | **Filtering of imputed genotypes**^1^ | **Data management and statistical analysis** | **Population stratification or principal components (PCs)** |
| --- | --- | --- | --- | --- | --- | --- | --- | --- | --- |
| **3 City Study** | Illumina 610K | Illumina | Call rate<98%,  MAF<1%,  pHWE<1E-6 | 537,029 | MACH version 1.0.15 | HapMap release 22 (build 36.3) | none | SAS, linear or logistic regression according to the type of outcome. Related individuals were excluded before data analysis. | none |
| **Blue Mountains Eye Study (BMES)** | Custom Illumina Infinium 670k array | Illumina | Call rate<95%,  MAF<1%,  pHWE<1E-6,  SNPs not in Hapmap or strandedness issues merging with Hapmap | 501,910 | MACH version 1.0.16 | HapMap release 22 (build 36) | MAF<1% Rsq<0.3 | SAS, linear and logistic models | No association was observed between either CKD or eGFR and the top 4 dimensions estimated using multi-dimensional scaling in PLINK.^[1]^ Therefore no ancestry dimensions were included in the analysis for association between genotype and either CKD or eGFR. |
| **CoLaus** | Affymetrix 500K | BRLMM | Call rate<70%,  MAF<1%  pHWE<1E-7 | 390,029 | IMPUTE version 0.2.0 | HapMap release 21 (build 35) | none | Matlab | First 4 ancestry PCs were significantly associated both with eGFR and CKD, thus were included in the analysis. |
| **Cardio-vascular risk in Young Finns Study (YFS)** | Illumina Custom BeadChip Human670K | Illumina | Call rate < 95%,  MAF<1%,  pHWE<1E-6,  pi-hat > 0.2 removed due to possible relatedness | 546,677 | MACH version 1.0 | HapMap 2 release 22 (NCBI build 36 dbSNP 126) | Rsq<0.2 | R, linear mixed effect models and GEE models, robust variance option to account for relatedness | We have used multidimensional scaling for genetic stratification and included the most affecting components in our model |
| **Estonian Genome Center** | Illumina 370CNV / OMNIExpress | Illumina Beadstudio/Genome Studio | Call rate <95%,  MAF < 1%,  pHWE<1E-6 | 183,887 | Impute version 1.0 | HapMap release 22 (build 36) | none | Data cleaning and relatedness: PLINK; Association testing: SNPTEST; R | No population stratification present, estimated using Eigenstrat^[2]^. |
| **Family Heart Study – II (FamHS-II)** | Illumina 1M | Illumina BeadStudio | call rate < 98%,  MAF < 1%,  pHWE<10E-6 | 456,293 | MACH | HapMap release 22 (build 36) | none | SAS, linear mixed effect models | The first 10 PCs estimated using Eigenstrat^[2]^ and field centers were included in the analysis for association between genotype and eGFR. |
| **GoDARTs** | Affymetrix 500K, Illumina 1.2M genotyping chip typed on the 1958 Birth Cohort (58C) and National Blood Service (NBS) individuals forming the common control group of WTCCC2 | Affymetrix | Call rate<97%,  MAF<1%,  pHWE<1E-6,  mishap p<1e-9,  Mendelian errors>100,  SNPs not in Hapmap or strandedness issues merging with Hapmap | 378,163 | IMPUTE version 2 | HapMap release 22 (build 36) | none | SNPTEST | We observed no association with CKD with the 10 PCs |
| **INGI – Carlantino Project** | Illumina 370K | Illumina | Call rate <90%,  MAF<5%,  pHWE<0.05 | 374,498 | MACH | HapMap release 22 (build 36) | MAF<5%,  Rsq<0.3 | R, GenABEL, mmscore | Because of the presence of close relatives in our dataset, statistical analyses were performed by using the kinship matrix, estimated through implementation in GenABEL |
| **INGI – Cilento Study** | Illumina 370K | Illumina | call rate<95%, SNPs not in Hapmap | 285,674 | MACH version 1.0.15 | HapMap release 22 (build 36) | none | R, linear model, GenABEL and ProbABEL (mmscore function was used to account for relatedness) | none |
| **INGI – FVG Project** | Illumina 370K | Illumina | Call rate<90%,  MAF<5%,  pHWE<0.05 | 374,498 | MACH | HapMap release 22 (build 36) | MAF<5%,  Rsq<0.3 | R, GenABEL, mmscore | Because of the presence of close relatives in our dataset, statistical analyses were performed by using kinship matrix, estimated through implementation in GenABEL |
| **INGI - Val Borbera Study** | Illumina SNP array 370K - HumanCNV370-Quadv3 | BeadStudio | Call rate<90%,  MAF<1%,  pHWE<1E-4 | 324,319 | MACH | HapMap release 22 (build 36) | MAF<1%  Rsq<0.3 | ProbABEL, linear mixed effect models to account for relatedness | We estimated PCs using PLINK. The first 3 PCs were included in the analysis for association between genotype and eGFR |
| **JUPITER** | Illumina Omni 1 Quad | Genome Studio | Call rate < 98.5%,  MAF<1%,  pHWE<1E-6 | 979,089 | MACH version 1.0.15 | 1000 Genomes, release 6/2010 | none | R | Ten PCs from EIGENSTRAT included |
| **Ogliastra Genetic Park (OGP) - Talana** | Affymetrix 500K | Affymetrix | Call rate<95%,  MAF<1%,  pHWE<1E-6 | 329,122 | MACH version 1.0.16 | HapMap release 22 (build 36) | Rsq<0.3 | R, GenABEL, ProbABEL (mmscore function was used to account for relatedness) | none |
| **PROSPER / PHASE Study** | Illumina 660K | Illumina | Call rate < 97.5% | 557,192 | MACH version 1.0.15 | HapMap release 22 (build 36) | none | R, SPSS, linear regression | We excluded outliers from the multi-dimensional scaling plot. Furthermore we adjusted our models for country of origin (The Netherlands, Scotland or Ireland). All subjects were of Caucasian origin. |

*Genotyping information for replication studies (KORA-F3 NGWA, KORA-F4 NGWA, Ogliastra Genetic Park, SAPALDIA, and SAPHIR) that genotyped replication SNPs *de novo* can be found in the Methods section.

^1^Rsq is the estimate of squared correlation between imputed and true genotypes provided by the imputation software MACH.

**References**

1.     Purcell S, Neale B, Todd-Brown K, Thomas L, Ferreira MA, et al. (2007) PLINK: A tool set for whole-genome association and population-based linkage analyses. Am J Hum Genet 81(3): 559-575. 10.1086/519795.

2.     Price AL, Patterson NJ, Plenge RM, Weinblatt ME, Shadick NA, et al. (2006) Principal components analysis corrects for stratification in genome-wide association studies. Nat Genet 38(8): 904-909. 10.1038/ng1847.
