# Supplementary material for: Genome-Wide Association and Functional Follow-Up Reveals New Loci for Kidney Function
Source: PLoS Genet. 2012 Mar 29;8(3):e1002584. doi: 10.1371/journal.pgen.1002584 (PMC3315455; doi:10.1371/journal.pgen.1002584)
Supplement: Table S7 — Top four SNPs from the CKD45 analysis. (DOC) [file pgen.1002584.s019.doc]

**Table S7. Top four SNPs from the CKD45 analysis.**

| **Locus description** | | | | | **Discovery meta-analysis** | |  | **Replication meta-analysis** | | | | **Combined meta-analysis** | | |
| --- | --- | --- | --- | --- | --- | --- | --- | --- | --- | --- | --- | --- | --- | --- |
| **SNP ID** | **Chr** | **Position**  **(bp)^†^** | **Genes**  **nearby^†^** | **Ref. All. (RAF)** | **OR (95%CI)** | ***P* value**^‡^ | **I^2^%** | **OR (95%CI)** | **1-sided *P* value** | **I^2^%** | **Q value** | **OR (95%CI)** | ***P* value** | **I^2^%** |
| rs4549145 | 2 | 48,555,686 | **KLRAQ1** | G(0.20) | 1.30 (1.16-1.45) | 2.9x10^-6^ | 0 | 0.93 (0.83-1.04) | 0.9008 | 0 | 0.3865 | 1.11 (1.03-1.20) | 0.0093 | 32 |
| rs9824190 | 3 | 98,163,910 | **EPHA6** | G(0.07) | 1.45 (1.24-1.70) | 3.2x10^-6^ | 0 | 1.03 (0.87-1.21) | 0.3758 | 50 | 0.2260 | 1.24 (1.11-1.38) | 0.0002 | 45 |
| rs2184241 | 9 | 3,574,112 | RFX3 | T(0.39) | 1.28 (1.15-1.42) | 1.9x10^-6^ | 9 | 0.99 (0.90-1.09) | 0.5573 | 0 | 0.2933 | 1.12 (1.05-1.20) | 0.0011 | 40 |
| rs11245299 | 10 | 126,253,483 | **LHPP** | C(0.73) | 1.30 (1.16-1.45) | 2.9x10^-6^ | 0 | 0.93 (0.85-1.03) | 0.9179 | 0 | 0.3865 | 1.08 (1.00-1.16) | 0.0371 | 37 |

**Abbreviations:** Chr.: chromosome; bp: base-pairs; Ref./Non-Ref. All.: reference/non-reference alleles; RAF: reference allele frequency; OR: Odds Ratio; 95%CI: 95% confidence interval.

**†**Positions and genes nearby were based on RefSeq genes (build 36). The gene closest to the SNP is listed first and is in boldface if the SNP is located within the gene.

‡Post GWAS meta-analysis genomic control correction applied to *P* values and standard errors.
