# Supplementary material for: Genome-Wide Association and Functional Follow-Up Reveals New Loci for Kidney Function
Source: PLoS Genet. 2012 Mar 29;8(3):e1002584. doi: 10.1371/journal.pgen.1002584 (PMC3315455; doi:10.1371/journal.pgen.1002584)
Supplement: Table S8 — Loci identified by the test for differential effects between strata in the GWAS. Results are sorted by trait, group and chromosome. For each SNP, the P value of the test for difference between strata is reported. (DOC) [file pgen.1002584.s020.doc]

**Table S8. Loci identified by the test for differential effects between strata in the GWAS. Results are sorted by trait, group and chromosome. For each SNP, the *P* value of the test for difference between strata is reported.**

| **Trait** | **SNPID** | **Chr** | **Position (bp)^1^** | **Genes nearby^1^** | **Ref. All. (RAF)** | **Group** | **Discovery analysis** | | **Replication analysis** | | **Combined analysis** | |
| --- | --- | --- | --- | --- | --- | --- | --- | --- | --- | --- | --- | --- |
|  |  |  |  |  |  |  | **Effect(SE)** | ***P* value for diff.** | **Effect(SE)** | ***P* value for diff.** | **Effect(SE)** | ***P* value for diff.** |
| eGFRcrea | rs1168357 | 2 | 170,004,477 | BBS5 | T(0.34) | non-DM | -0.0005(0.0013) | 2.4E-07 | 0.0000(0.0014) | 0.0648 | -0.0002(0.0009) | 2.3E-07 |
|  |  |  |  |  |  | DM | 0.0252(0.0048) |  | 0.0103(0.0054) |  | 0.0175(0.0033) |  |
|  | rs1500896 | 8 | 96,555,858 | C8orf37 | A(0.42) | non-DM | 0.0034(0.0013) | 4.6E-07 | 0.0008(0.0014) | 0.7664 | 0.0022(0.0009) | 0.0019 |
|  |  |  |  |  |  | DM | -0.0207(0.0046) |  | 0.0024(0.0052) |  | -0.0081(0.0032) |  |
|  | rs752805 | 11 | 125,798,495 | KIRREL3 | A(0.48) | non-DM | -0.0013(0.0013) | 1.6E-06 | -0.0011(0.0013) | 0.6759 | -0.0012(0.0009) | 0.0039 |
|  |  |  |  |  |  | DM | 0.0221(0.0047) |  | -0.0033(0.0051) |  | 0.0084(0.0032) |  |
|  | rs17446008 | 18 | 23,875,364 | **CDH2** | A(0.12) | no HTN | -0.0034(0.0033) | 1.1E-06 | -0.0005(0.0029) | 0.9275 | -0.0018(0.0022) | 0.0064 |
|  |  |  |  |  |  | HTN | 0.0238(0.0045) |  | -0.0009(0.0033) |  | 0.0077(0.0027) |  |
|  | rs11935537 | 4 | 130,709,590 | C4orf33 | C(0.84) | younger | 0.0034(0.0019) | 1.5E-06 | 0.0004(0.0022) | 0.5827 | 0.0021(0.0014) | 0.0043 |
|  |  |  |  |  |  | older | -0.0145(0.0032) |  | 0.0024(0.0029) |  | -0.0051(0.0021) |  |
|  | rs13014379 | 2 | 28,966,356 | WDR43 | T(0.79) | women | -0.0031(0.0020) | 6.1E-06 | 0.0008(0.0024) | 0.1970 | -0.0015(0.0015) | 0.0639 |
|  |  |  |  |  |  | men | 0.0121(0.0027) |  | -0.0034(0.0022) |  | 0.0027(0.0017) |  |
|  | rs10068737 | 5 | 118,064,446 | DTWD2 | T(0.55) | women | 0.0020(0.0015) | 8.7E-07 | -0.0012(0.0021) | 0.6462 | 0.0009(0.0012) | 1.5E-04 |
|  |  |  |  |  |  | men | -0.0103(0.0020) |  | -0.0025(0.0019) |  | -0.0061(0.0014) |  |
|  | rs7911360 | 10 | 122,795,537 | RPL19P16 | A(0.37) | women | 0.0016(0.0015) | 7.0E-06 | -0.0012(0.0019) | 0.7241 | 0.0005(0.0012) | 8.5E-04 |
|  |  |  |  |  |  | men | -0.0100(0.0021) |  | -0.0021(0.0017) |  | -0.0054(0.0013) |  |
| CKD | rs1322199 | 6 | 165,345,507 | PDE10A | A(0.71) | non-DM | 0.0140(0.0234) | 8.9E-06 | 0.0113(0.0092) | 0.9403 | 0.0117(0.0086) | 0.0038 |
|  |  |  |  |  |  | DM | -0.2490(0.0544) |  | 0.0081(0.0417) |  | -0.0870(0.0330) |  |
|  | rs500456 | 7 | 54,566,276 | VSTM2A | A(0.58) | non-DM | -0.0172(0.0228) | 1.0E-06 | -0.0001(0.0085) | 0.0912 | -0.0023(0.0080) | 3.4E-05 |
|  |  |  |  |  |  | DM | 0.2722(0.0547) |  | 0.0632(0.0365) |  | 0.1279(0.0304) |  |
|  | rs4149333 | 9 | 106,592,745 | **ABCA1** | A(0.88) | non-DM | 0.0308(0.0349) | 5.3E-06 | -0.0158(0.0172) | 0.6060 | -0.0067(0.0154) | 0.0018 |
|  |  |  |  |  |  | DM | -0.3535(0.0769) |  | -0.0471(0.0582) |  | -0.1595(0.0464) |  |

**Abbreviations:** Chr.: chromosome; bp: base-pairs; Ref. All.: reference allele; RAF: reference allele frequency; SE: standard error.

^1^Positions and genes nearby were based on RefSeq genes (build 36). The gene closest to the SNP is listed first and is in boldface if the SNP is located within the gene.
