# Supplementary material for: Genome-Wide Association and Functional Follow-Up Reveals New Loci for Kidney Function
Source: PLoS Genet. 2012 Mar 29;8(3):e1002584. doi: 10.1371/journal.pgen.1002584 (PMC3315455; doi:10.1371/journal.pgen.1002584)
Supplement: Table S9 — Imputation quality of replicated SNPs in all discovery and replication studies: median MACH-Rsq and interquartile range (IQR) are reported. (DOC) [file pgen.1002584.s021.doc]

**Supplementary Table 9. Imputation quality of replicated SNPs in all discovery and replication studies: median MACH-Rsq and interquartile range (IQR) are reported.**

| **SNPID** | **Locus name** | **Median Rsq*** | **IQR** |
| --- | --- | --- | --- |
| rs3925584 | *MPPED2* | 0.995 | 0.942; 0.999 |
| rs6431731 | *DDX1* | 0.632 | 0.591; 0.655 |
| rs12124078 | *CASP9* | 0.999 | 0.997; 1.000 |
| rs2453580 | *SLC47A1* | 0.966 | 0.715; 0.989 |
| rs11078903 | *CDK12* | 0.921 | 0.913; 0.923 |
| rs2928148 | *INO80* | 0.998 | 0.990; 0.999 |

*Results do not include the Sorbs study, for which genotype imputation was performed with a different software (IMPUTE). The "proper-info" index for the six SNPs in the Sorbs study was 0.999, 0.546, 1.000, 0.644, 0.914, and 0.987, respectively.
