# Supplementary material for: Genome-Wide Association and Functional Follow-Up Reveals New Loci for Kidney Function
Source: PLoS Genet. 2012 Mar 29;8(3):e1002584. doi: 10.1371/journal.pgen.1002584 (PMC3315455; doi:10.1371/journal.pgen.1002584)
Supplement: Table S10 — Effects of novel and known loci on log(eGFRcrea) in the overall population. (DOC) [file pgen.1002584.s022.doc]

**Table S10.** Effects of novel and known loci on log(eGFRcrea) in the overall population.

|  | **SNP ID** | **Locus name** | **Ref. All.** | **RAF** | **Effect** | **SE** | ***P* value** | **I^2^ (%)** |
| --- | --- | --- | --- | --- | --- | --- | --- | --- |
|  |  | | | |  |  |  |  |
| **Novel loci (sample size 130,600)** | rs3925584 | *MPPED2* | T | 0.54 | -0.0080 | 0.0009 | 8.4E-18 | 21 |
|  | rs6431731 | *DDX1* | T | 0.94 | -0.0130 | 0.0023 | 4.3E-08 | 11 |
|  | rs12124078* | *CASP9* | A | 0.70 | 0.0091 | 0.0010 | 9.5E-22 | 67 |
|  | rs2453580 | *SLC47A1* | T | 0.59 | 0.0052 | 0.0010 | 5.2E-08 | 22 |
|  | rs11078903 | *CDK12* | A | 0.76 | -0.0070 | 0.0011 | 3.1E-09 | 0 |
|  | rs2928148 | *INO80* | A | 0.52 | 0.0051 | 0.0009 | 4.0E-08 | 0 |
|  |  | | | | | | | |
| **Known loci^[1, 2]^ possibly related to renal function (sample size 74,354)** | rs10109414 | *STC1* | T | 0.42 | -0.0080 | 0.0013 | 2.1E-09 | 24 |
|  | rs11959928 | *DAB2* | A | 0.44 | -0.0090 | 0.0013 | 5.2E-13 | 8 |
|  | rs12460876 | *SLC7A9* | T | 0.61 | -0.0090 | 0.0013 | 8.6E-11 | 11 |
|  | rs1260326 | *GCKR* | T | 0.41 | 0.0089 | 0.0013 | 4.5E-12 | 15 |
|  | rs12917707 | *UMOD* | T | 0.18 | 0.0168 | 0.0017 | 2.2E-23 | 70 |
|  | rs13538 | *ALMS1* | A | 0.77 | -0.0100 | 0.0016 | 7.0E-10 | 9 |
|  | rs1394125 | *UBE2Q2* | A | 0.35 | -0.0090 | 0.0015 | 7.4E-11 | 3 |
|  | rs17319721 | *SHROOM3* | A | 0.43 | -0.0120 | 0.0013 | 1.5E-21 | 0 |
|  | rs267734 | *LASS2* | T | 0.80 | -0.0090 | 0.0016 | 3.7E-09 | 27 |
|  | rs347685 | *TFDP2* | A | 0.72 | -0.0080 | 0.0014 | 5.0E-09 | 0 |
|  | rs4744712 | *PIP5K1B* | A | 0.39 | -0.0080 | 0.0013 | 7.7E-11 | 19 |
|  | rs626277 | *DACH1* | A | 0.60 | -0.0080 | 0.0014 | 2.9E-10 | 17 |
|  | rs6420094 | *SLC34A1* | A | 0.66 | 0.0111 | 0.0015 | 5.7E-14 | 0 |
|  | rs881858 | *VEGFA* | A | 0.71 | -0.0100 | 0.0015 | 1.6E-10 | 8 |
|  | rs7805747 | *PRKAG2* | A | 0.24 | -0.0130 | 0.0018 | 2.7E-13 | 27 |
|  |  | | | | | | | |
| **Known loci^[1, 2]^ possibly related to creatinine metabolism (sample size 74,354)** | rs10774021 | *SLC6A13* | T | 0.64 | -0.0070 | 0.0014 | 4.7E-07 | 27 |
|  | rs10794720 | *WDR37* | T | 0.08 | -0.0130 | 0.0024 | 5.0E-08 | 0 |
|  | rs2279463 | *SLC22A2* | A | 0.88 | 0.0133 | 0.0020 | 1.1E-11 | 27 |
|  | rs491567 | *WDR72* | A | 0.78 | -0.0100 | 0.0016 | 3.5E-11 | 6 |
|  | rs6465825 | *TMEM60* | T | 0.60 | 0.0072 | 0.0013 | 2.0E-08 | 15 |
|  | rs7422339 | *CPS1* | A | 0.31 | -0.0100 | 0.0015 | 1.3E-10 | 0 |
|  | rs9895661 | *BCAS3* | T | 0.82 | 0.0095 | 0.0018 | 4.9E-08 | 0 |
|  | rs2453533 | *GATM* | A | 0.38 | -0.0130 | 0.0013 | 4.0E-22 | 0 |

**Abbreviations:** Ref. All.: reference allele; RAF: reference allele frequency; SE: standard error.

*Although the effect of this SNP was not significantly different between strata of age (**Table S18**), most of the heterogeneity observed in the overall sample (I^2^=67%) was explained by the age stratification. In fact this locus was uncovered in the younger group, where I^2^ was as low as 20% (**Table 1**).

References

1.     Kottgen A, Glazer NL, Dehghan A, Hwang SJ, Katz R, et al. (2009) Multiple loci associated with indices of renal function and chronic kidney disease. Nat Genet 41(6): 712-717.

2.     Kottgen A, Pattaro C, Boger CA, Fuchsberger C, Olden M, et al. (2010) New loci associated with kidney function and chronic kidney disease. Nat Genet 42(5): 376-384.
