# Supplementary material for: Genome-Wide Association and Functional Follow-Up Reveals New Loci for Kidney Function
Source: PLoS Genet. 2012 Mar 29;8(3):e1002584. doi: 10.1371/journal.pgen.1002584 (PMC3315455; doi:10.1371/journal.pgen.1002584)
Supplement: Table S11 — Genes nearest to loci associated with renal traits. (DOC) [file pgen.1002584.s023.doc]

**Table S11. Genes nearest to loci associated with renal traits.**

| **Location, lead SNP** | **Locus gene(s)*** | **Gene description**  (official gene name is reported in *Italic*)^†^ |
| --- | --- | --- |
| 11p13, rs3925584 | *MPPED2* | *Metallophosphoesterase domain containing 2*. This gene likely encodes a metallophosphoesterase. The encoded protein may play a role in brain development. SNP rs3925584 has also been identified in a GWAS of serum magnesium concentrations, and was associated nominally associated with eGFR in that study.^[1]^ |
|  | C11orf46 | *Chromosome 11 open reading frame 46*. No publications linking this gene to kidney function. |
| 2p24, rs6431731 | *DDX1* | *DEAD (Asp-Glu-Ala-Asp) box polypeptide 1*. *DDX1* shows high transcription levels in 2 retinoblastoma cell lines and in tissues of neuroectodermal origin,^[2]^ and has a reported role in testicular tumorigenesis.^[3]^ There are no publications linking this gene to kidney function. |
|  | *MYCN* | *V-myc myelocytomatosis viral related oncogene, neuroblastoma derived (avian)*. *MYCN* is an oncogene amplified in a subset of neuroblastomas and Wilms’ tumor, and a *MYCN* copy number gain was present in about 10% of patients with Wilms’ tumor in a genome-wide analysis.^[4]^ Mutations in *MYCN* were found in patients with Feingold Syndrome, an autosomal dominant disorder characterized by variable combinations of esophageal and duodenal atresias, microcephaly, learning disability, syndactyly, and cardiac defects. |
|  | *NBAS* | *Neuroblastoma amplified sequence.* There are no publications linking this gene to kidney function. |
| 17p11.2, rs2453580 | ***SLC47A1*** | *Solute carrier family 47, member 1*. *SLC47A1* encodes the multidrug and toxin extrusion transporter (*MATE1*) that excretes endogenous and exogenous substances through urine and bile. In the kidney, it is expressed in the brush border membrane of proximal and distal tubular cells.^[5]^ Here it represents the apical exit transporter for creatinine and metformin and other organic cations, many of which are thought to enter the cell at the basolateral membrane via the organic cation transporter 2, encoded by *SLC22A2*.^[6]^ Significant associations between SNPs in *SLC22A2* and eGFR were identified in our earlier GWAS.^[7]^ |
| 1p36.1, rs12124078 | ***DNAJC16*** | *DnaJ (Hsp40) homolog, subfamily C, member 16*. Integral membrane protein (geneCards), expressed in human renal tissue. Function not known. |
|  | *CASP9* | *Caspase 9, apoptosis-related cysteine peptidase*. The *CASP9* gene encodes the third apoptotic activation factor that is involved in the activation of cell apoptosis, necrosis and inflammation. In the kidney, caspase-9 may play an important role in the medulla response to hyperosmotic stress^[8]^ and in cadmium-induced toxicity.^[9]^ |
|  | *AGMAT* | *Agmatine ureohydrolase (agmatinase)*. Mitochondrial enzyme that catalyzes the reaction of agmatine, a breakdown product of arginine, and H2O to putrescine and urea. It is most abundantly expressed in human liver and kidney.^[10]^ In normal kidney, expression is restricted to tubulus epithelial cells, but different expression patterns have been observed in renal clear cell carcinoma.^[11]^ |
| 17q12, rs11078903 | ***CDK12*** | *Cyclin-dependent kinase 12*. Differential expression of cyclin-dependent kinase inhibitors have been described in human glomerular disease.^[12]^ |
|  | *MED1* | *Mediator complex subunit 1* (previous name: PPAR binding protein). There are no publications linking this gene to kidney function. The protein encoded by *MED1* binds to several nuclear receptors such as *PPARα*, *RARα*, *TRβ1*, *RXR*, *VDR*, *FXR*, *ERα* and *GR* and may therefore play a role in nuclear receptor mediated processes such as homeostatic regulation.^[13]^ |
|  | Other genes in this LD block include *LOC90110*, *FBXL20*, *NEUROD2*, *PPP1R1B*, *STARD3*, *PNMT*, *TCAP*, *PGAP3*, *ERBB2*, and *C17orf37* | *PNMT* belongs to the catecholaminergic pathway and has been associated with hypertension^[14]^ and adverse outcomes in acute kidney injury.^[15]^ Abnormal *ERBB2* expression is observed in human autosomal recessive polycystic kidney disease (PKD).^[16]^ Inhibition of HER-2(neu/ErbB2) restores normal function and structure to PKD epithelia.^[17]^ |
| 15q15.1, rs2928148 | ***INO80*** | *INO80 homolog (S. cerevisiae)*. *INO80* encodes a nuclear enzyme and belongs to a highly conserved complex that is involved in nucleosome remodeling. |
|  | *EXD1* | *Exonuclease 3'-5' domain containing 1*. Expressed in human renal tissue, no relation to kidney function known. |
|  | *CHAC1* | *ChaC, cation transport regulator homolog 1 (E. coli)*. Expressed in human renal tissue. No obvious relation to renal function, may have a role in the unfolded protein response pathway.^[18]^ |

*Bold means the SNP is in the gene. ^†^HUGO Gene Nomenclature Committee (HGNC), EMBL Outstation - Hinxton, European Bioinformatics Institute, Wellcome Trust Genome Campus, Hinxton, Cambridgeshire, CB10 1SD, UK, [www.genenames.org](http://www.genenames.org/).^[19]^ Accessed on: Aug 4th, 2011

References

1.     Meyer TE, Verwoert GC, Hwang SJ, Glazer NL, Smith AV, et al. (2010) Genome-wide association studies of serum magnesium, potassium, and sodium concentrations identify six loci influencing serum magnesium levels. PLoS Genet 6(8): e1001045.

2.     Godbout R, Squire J. (1993) Amplification of a DEAD box protein gene in retinoblastoma cell lines. Proc Natl Acad Sci U S A 90(16): 7578-7582.

3.     Tanaka K, Okamoto S, Ishikawa Y, Tamura H, Hara T. (2009) DDX1 is required for testicular tumorigenesis, partially through the transcriptional activation of 12p stem cell genes. Oncogene 28(21): 2142-2151.

4.     Williams RD, Al-Saadi R, Chagtai T, Popov S, Messahel B, et al. (2010) Subtype-specific FBXW7 mutation and MYCN copy number gain in wilms' tumor. Clin Cancer Res 16(7): 2036-2045.

5.     Otsuka M, Matsumoto T, Morimoto R, Arioka S, Omote H, et al. (2005) A human transporter protein that mediates the final excretion step for toxic organic cations. Proc Natl Acad Sci U S A 102(50): 17923-17928.

6.     Nies AT, Koepsell H, Damme K, Schwab M. (2011) Organic cation transporters (OCTs, MATEs), in vitro and in vivo evidence for the importance in drug therapy. Handb Exp Pharmacol (201)(201): 105-167.

7.     Kottgen A, Pattaro C, Boger CA, Fuchsberger C, Olden M, et al. (2010) New loci associated with kidney function and chronic kidney disease. Nat Genet 42(5): 376-384.

8.     Allan LA, Clarke PR. (2009) Apoptosis and autophagy: Regulation of caspase-9 by phosphorylation. FEBS J 276(21): 6063-6073.

9.     Gobe G, Crane D. (2010) Mitochondria, reactive oxygen species and cadmium toxicity in the kidney. Toxicol Lett 198(1): 49-55.

10.     Iyer RK, Kim HK, Tsoa RW, Grody WW, Cederbaum SD. (2002) Cloning and characterization of human agmatinase. Mol Genet Metab 75(3): 209-218.

11.     Dallmann K, Junker H, Balabanov S, Zimmermann U, Giebel J, et al. (2004) Human agmatinase is diminished in the clear cell type of renal cell carcinoma. Int J Cancer 108(3): 342-347.

12.     Shankland SJ, Eitner F, Hudkins KL, Goodpaster T, D'Agati V, et al. (2000) Differential expression of cyclin-dependent kinase inhibitors in human glomerular disease: Role in podocyte proliferation and maturation. Kidney Int 58(2): 674-683.

13.     Viswakarma N, Jia Y, Bai L, Vluggens A, Borensztajn J, et al. (2010) Coactivators in PPAR-regulated gene expression. PPAR Res 2010: 250126.

14.     Cui J, Zhou X, Chazaro I, DeStefano AL, Manolis AJ, et al. (2003) Association of polymorphisms in the promoter region of the PNMT gene with essential hypertension in african americans but not in whites. Am J Hypertens 16(10): 859-863.

15.     Alam A, O'Connor DT, Perianayagam MC, Kolyada AY, Chen Y, et al. (2010) Phenylethanolamine N-methyltransferase gene polymorphisms and adverse outcomes in acute kidney injury. Nephron Clin Pract 114(4): c253-9.

16.     Nakanishi K, Sweeney W,Jr, Avner ED. (2001) Segment-specific c-ErbB2 expression in human autosomal recessive polycystic kidney disease. J Am Soc Nephrol 12(2): 379-384.

17.     Wilson SJ, Amsler K, Hyink DP, Li X, Lu W, et al. (2006) Inhibition of HER-2(neu/ErbB2) restores normal function and structure to polycystic kidney disease (PKD) epithelia. Biochim Biophys Acta 1762(7): 647-655.

18.     Mungrue IN, Pagnon J, Kohannim O, Gargalovic PS, Lusis AJ. (2009) CHAC1/MGC4504 is a novel proapoptotic component of the unfolded protein response, downstream of the ATF4-ATF3-CHOP cascade. J Immunol 182(1): 466-476.

19.     Seal RL, Gordon SM, Lush MJ, Wright MW, Bruford EA. (2011) Genenames.org: The HGNC resources in 2011. Nucleic Acids Res 39(Database issue): D514-9.
