# Supplementary material for: Genome-Wide Association and Functional Follow-Up Reveals New Loci for Kidney Function
Source: PLoS Genet. 2012 Mar 29;8(3):e1002584. doi: 10.1371/journal.pgen.1002584 (PMC3315455; doi:10.1371/journal.pgen.1002584)
Supplement: Table S12 — Imputation Quality (MACH-Rsq) for the best SNPs in the African ancestry samples of the CARe consortium (1.00 refers to genotyped data). (DOC) [file pgen.1002584.s024.doc]

**Table S12.** Imputation Quality (MACH-Rsq) for the best SNPs in the African ancestry samples of the CARe consortium (1.00 refers to genotyped data)

| **SNP** | **ARIC** | **CARDIA** | **JHS** | **MESA** |
| --- | --- | --- | --- | --- |
| rs12278026 | 0.60 | 0.66 | 0.66 | 0.64 |
| rs4669002 | 0.81 | 0.84 | 0.86 | 0.87 |
| rs1472554 | 1.00 | 1.00 | 1.00 | 1.00 |
| rs1800869 | 1.00 | 1.00 | 1.00 | 1.00 |
| rs1874226 | 0.94 | 1.03 | 0.99 | 0.97 |
| rs8039934 | 1.00 | 1.00 | 1.00 | 1.00 |
