# Supplementary material for: Genome-Wide Association and Functional Follow-Up Reveals New Loci for Kidney Function
Source: PLoS Genet. 2012 Mar 29;8(3):e1002584. doi: 10.1371/journal.pgen.1002584 (PMC3315455; doi:10.1371/journal.pgen.1002584)
Supplement: Table S13 — Baseline characteristics of the kidney biopsies for the eQTL analysis. (DOC) [file pgen.1002584.s025.doc]

**Table S13.** Baseline characteristics of the kidney biopsies for the eQTL analysis.

|  | **Cohort 1** | **Cohort 2** |
| --- | --- | --- |
| **Sample size, n** | 56 | 25 |
| **Age, mean (range)** | 65 (29–87) | 62 (36–84) |
| **Males, n ( %)** | 32 (57%) | 18 (72%) |
| **Caucasians, n (%)** | 43 (76%) | 16 (64%) |
