# Supplementary material for: Genome-Wide Association and Functional Follow-Up Reveals New Loci for Kidney Function
Source: PLoS Genet. 2012 Mar 29;8(3):e1002584. doi: 10.1371/journal.pgen.1002584 (PMC3315455; doi:10.1371/journal.pgen.1002584)
Supplement: Table S14 — Analysis of the new loci for eQTL status in meta-analysis of two cohorts of kidney biopsies. (DOC) [file pgen.1002584.s026.doc]

**Table S14.** Analysis of the new loci for eQTL status in meta-analysis of two cohorts of kidney biopsies.

| **SNPID**  (lead SNP) | **Locus name** | **eQTL SNP**† | **R^2^**‡ | **Expression probe**§ | **Expression Gene*** | ***P* value eQTL**** |
| --- | --- | --- | --- | --- | --- | --- |
| rs3925584 | *MPPED2* | rs10767873 | 0.982 | 205413_at | *MPPED2* | 0.4250 |
| rs3925584 |  | rs10767873 | 0.982 | 215692_s_at | *MPPED2* | 0.6090 |
| rs6431731 | *DDX1* | rs11902962 | 0.234 | 207028_at | *MYCNOS* | 0.2710 |
| rs6431731 |  | rs11902962 | 0.234 | 216188_at | *MYCNOS* | 0.5760 |
| rs12124078 | *CASP9* | rs12124078 | NA | 212908_at | *DNAJC16* | 0.7890 |
| rs12124078 |  | rs12124078 | NA | 212911_at | *DNAJC16* | 0.0480 |
| rs12124078 |  | rs12124078 | NA | 217358_at | *DNAJC16* | 0.9820 |
| rs2453580 | *SLC47A1* | rs2018675 | 0.353 | 219525_at | *SLC47A1* | 0.9600 |
| rs11078903 | *CDK12* | rs12936996 | 0.910 | 213557_at | *CDK12* | 0.2580 |
| rs11078903 |  | rs12936996 | 0.910 | 219226_at | *CDK12* | 0.3080 |
| rs11078903 |  | rs12936996 | 0.910 | 225690_at | *CDK12* | 0.0908 |
| rs11078903 |  | rs12936996 | 0.910 | 225691_at | *CDK12* | 0.6550 |
| rs11078903 |  | rs12936996 | 0.910 | 225694_at | *CDK12* | 0.1980 |
| rs11078903 |  | rs12936996 | 0.910 | 225697_at | *CDK12* | 0.5250 |
| rs2928148 | *INO80* | rs2306083 | 0.896 | 225357_s_at | *INO80* | 0.1950 |
| rs2928148 |  | rs2306083 | 0.896 | 229356_x_at | *INO80* | 0.0872 |

†eQTL probe rsID with strongest linkage disequilibrium (R^2^) to the locus lead SNP; in case of equal R^2^ the probe with shortest distance to the lead SNP was chosen.

‡Linkage disequilibrium (R^2^) for eQTL probe with the lead SNP.

§Expression probe identifier (Affymetrix U133set).

*Gene symbol associated with expression probe.

**Fisher’s method combined *P* value for eQTL. None of the eQTLs met the Bonferroni corrected cutoff of 1.61x10^-3^.
