# Supplementary material for: Genome-Wide Association and Functional Follow-Up Reveals New Loci for Kidney Function
Source: PLoS Genet. 2012 Mar 29;8(3):e1002584. doi: 10.1371/journal.pgen.1002584 (PMC3315455; doi:10.1371/journal.pgen.1002584)
Supplement: Table S15 — Association of novel and known loci with CKD and CKD45: Odds Ratios (OR), 95% confidence intervals (95%CI) and P values. (DOC) [file pgen.1002584.s027.doc]

**Table S15. Association of novel and known loci with CKD and CKD45: Odds Ratios (OR), 95% confidence intervals (95%CI) and *P* values.**

| **SNP ID** | **Chr** | **Position^†^** | | **Locus name** | | **Ref. / Non-Ref. all. (RAF)** | **CKD** | | | **CKD45** | | |
| --- | --- | --- | --- | --- | --- | --- | --- | --- | --- | --- | --- | --- |
|  |  |  |  |  |  |  | **OR** | **95%CI** | ***P* value** | **OR** | **95%CI** | ***P* value** |
|  |  |  | |  | |  |  |  |  |  |  |  |
| **Novel loci** |  |  | |  | |  |  |  |  |  |  |  |
| rs3925584 | 11 | 30,716,911 | | *MPPED2* | | T/C(0.54) | 1.02 | (1.01, 1.03) | 0.0021 | 1.12 | (1.06, 1.20) | 0.0002 |
| rs6431731 | 2 | 15,780,453 | | *DDX1* | | T/C(0.94) | 1.05 | (1.00, 1.11) | 0.0435 | 1.10 | (0.93, 1.30) | 0.2869 |
| rs12124078 | 1 | 15,742,486 | | *CASP9* | | A/G(0.70) | 0.99 | (0.97, 1.01) | 0.1767 | 0.99 | (0.92, 1.05) | 0.6716 |
| rs2453580 | 17 | 19,378,913 | | *SLC47A1* | | T/C(0.59) | 0.99 | (0.97, 1.01) | 0.1621 | 0.97 | (0.91, 1.05) | 0.4694 |
| rs11078903 | 17 | 34,885,450 | | *CDK12* | | A/G(0.76) | 1.01 | (0.99, 1.02) | 0.4988 | 0.97 | (0.89, 1.04) | 0.3707 |
| rs2928148 | 15 | 39,188,842 | | *INO80* | | A/G(0.52) | 0.98 | (0.97, 1.00) | 0.0230 | 0.93 | (0.87, 0.99) | 0.0237 |
|  |  |  | |  | |  |  |  |  |  |  |  |
| **Known loci^[1, 2]^ possibly related to renal function** | | | | | | | | | | | | |
| rs10109414 | 8 | | 23,807,096 | | *STC1* | T/C(0.42) | 1.05 | (1.01, 1.10) | 0.0112 | 1.08 | (0.98, 1.18) | 0.1222 |
| rs11959928 | 5 | | 39,432,889 | | *DAB2* | A/T(0.44) | 1.09 | (1.05, 1.14) | 1.3E-05 | 1.14 | (1.04, 1.26) | 0.0052 |
| rs12460876 | 19 | | 38,048,731 | | *SLC7A9* | T/C(0.61) | 1.06 | (1.02, 1.10) | 0.0055 | 1.03 | (0.94, 1.13) | 0.5326 |
| rs1260326 | 2 | | 27,584,444 | | *GCKR* | T/C(0.41) | 0.97 | (0.93, 1.01) | 0.1078 | 1.02 | (0.93, 1.12) | 0.7061 |
| rs12917707 | 16 | | 20,275,191 | | *UMOD* | T/G(0.18) | 0.79 | (0.75, 0.84) | 3.7E-16 | 0.74 | (0.65, 0.85) | 1.1E-05 |
| rs13538 | 2 | | 73,721,836 | | *ALMS1* | A/G(0.77) | 1.04 | (0.99, 1.10) | 0.0924 | 1.13 | (1.00, 1.27) | 0.0465 |
| rs1394125 | 15 | | 73,946,038 | | *UBE2Q2* | A/G(0.35) | 1.08 | (1.03, 1.13) | 0.0005 | 1.13 | (1.02, 1.25) | 0.0158 |
| rs17319721 | 4 | | 77,587,871 | | *SHROOM3* | A/G(0.43) | 1.07 | (1.03, 1.12) | 0.0005 | 1.06 | (0.97, 1.16) | 0.2226 |
| rs267734 | 1 | | 149,218,101 | | *LASS2* | T/C(0.80) | 1.07 | (1.02, 1.13) | 0.0056 | 1.00 | (0.90, 1.12) | 0.9506 |
| rs347685 | 3 | | 143,289,827 | | *TFDP2* | A/C(0.72) | 1.08 | (1.03, 1.13) | 0.0013 | 1.18 | (1.06, 1.31) | 0.0023 |
| rs4744712 | 9 | | 70,624,527 | | *PIP5K1B* | A/C(0.39) | 1.07 | (1.03, 1.11) | 0.0015 | 1.10 | (1.00, 1.21) | 0.0421 |
| rs626277 | 13 | | 71,245,697 | | *DACH1* | A/C(0.60) | 1.06 | (1.02, 1.11) | 0.0028 | 1.07 | (0.97, 1.18) | 0.1530 |
| rs6420094 | 5 | | 176,750,242 | | *SLC34A1* | A/G(0.66) | 0.92 | (0.88, 0.96) | 0.0002 | 0.82 | (0.74, 0.91) | 0.0002 |
| rs881858 | 6 | | 43,914,587 | | *VEGFA* | A/G(0.71) | 1.08 | (1.03, 1.13) | 0.0024 | 1.00 | (0.89, 1.11) | 0.9531 |
| rs7805747 (CKD) | 7 | | 151,038,734 | | *PRKAG2* | A/G(0.24) | 1.19 | (1.13, 1.26) | 6.0E-10 | 1.28 | (1.13, 1.46) | 0.0001 |
| rs653178 (eGFRcys) | 12 | | 110,492,139 | | *ATXN2* | T/C(0.51) | 0.97 | (0.93, 1.01) | 0.1461 | 1.02 | (0.93, 1.12) | 0.7040 |
|  |  | |  | |  |  |  |  |  |  |  |  |
| **Known loci^[1, 2]^ suspected to be related to creatinine metabolism** | | | | | | | | | | | | |
| rs10774021 | 12 | | 219,559 | | *SLC6A13* | T/C(0.64) | 1.04 | (1.00, 1.09) | 0.0516 | 1.14 | (1.03, 1.26) | 0.0118 |
| rs10794720 | 10 | | 1,146,165 | | *WDR37* | T/C(0.08) | 1.14 | (1.05, 1.22) | 0.0008 | 1.24 | (1.05, 1.47) | 0.0114 |
| rs2279463 | 6 | | 160,588,379 | | *SLC22A2* | A/G(0.88) | 0.90 | (0.85, 0.96) | 0.0007 | 0.90 | (0.79, 1.03) | 0.1359 |
| rs491567 | 15 | | 51,733,885 | | *WDR72* | A/C(0.78) | 1.07 | (1.02, 1.12) | 0.0080 | 1.04 | (0.94, 1.16) | 0.4455 |
| rs6465825 | 7 | | 77,254,375 | | *TMEM60* | T/C(0.60) | 0.97 | (0.93, 1.01) | 0.0924 | 0.99 | (0.91, 1.09) | 0.9055 |
| rs7422339 | 2 | | 211,248,752 | | *CPS1* | A/C(0.31) | 1.13 | (1.07, 1.18) | 8.0E-07 | 1.09 | (0.97, 1.22) | 0.1412 |
| rs9895661 | 17 | | 56,811,371 | | *BCAS3* | T/C(0.82) | 0.95 | (0.90, 1.01) | 0.0929 | 0.95 | (0.84, 1.08) | 0.4768 |
| rs2453533 | 15 | | 43,428,517 | | *GATM* | A/C(0.38) | 1.11 | (1.06, 1.15) | 1.3E-06 | 1.14 | (1.04, 1.25) | 0.0073 |

**Abbreviations:** Ref. All.: reference allele; RAF: reference allele frequency.

**^†^**Based on RefSeq genes (build 36).

**References**

1.     Kottgen A, Glazer NL, Dehghan A, Hwang SJ, Katz R, et al. (2009) Multiple loci associated with indices of renal function and chronic kidney disease. Nat Genet 41(6): 712-717.

2.     Kottgen A, Pattaro C, Boger CA, Fuchsberger C, Olden M, et al. (2010) New loci associated with kidney function and chronic kidney disease. Nat Genet 42(5): 376-384.
