# Supplementary material for: Genome-Wide Association and Functional Follow-Up Reveals New Loci for Kidney Function
Source: PLoS Genet. 2012 Mar 29;8(3):e1002584. doi: 10.1371/journal.pgen.1002584 (PMC3315455; doi:10.1371/journal.pgen.1002584)
Supplement: Table S19 — Association between novel and known loci and log(eGFRcrea) in females and in males and test for difference between strata. (DOC) [file pgen.1002584.s031.doc]

**Table S19. Association between novel and known loci and log(eGFRcrea) in females and in males and test for difference between strata.**

| **SNP ID** | **Locus name** | **Ref. All.** | **Females** | | | **Males** | | | **Test for different effects between groups: *P* value** |
| --- | --- | --- | --- | --- | --- | --- | --- | --- | --- |
|  |  |  | **Effect** | **SE** | ***P* value** | **Effect** | **SE** | ***P* value** |  |
|  | | | | | | | | | |
| **Novel loci** | | | | | | | | | |
| rs3925584 | *MPPED2* | T | -0.0074 | 0.0012 | 1.2E-10 | -0.0079 | 0.0013 | 8.2E-10 | 0.7775 |
| rs6431731 | *DDX1* | T | -0.0104 | 0.0030 | 6.2E-04 | -0.0127 | 0.0035 | 2.3E-04 | 0.6178 |
| rs12124078 | *CASP9* | A | 0.0080 | 0.0013 | 3.2E-10 | 0.0070 | 0.0014 | 1.0E-06 | 0.6007 |
| rs2453580 | *SLC47A1* | T | 0.0059 | 0.0012 | 2.2E-06 | 0.0040 | 0.0014 | 0.0045 | 0.3028 |
| rs11078903 | *CDK12* | A | -0.0072 | 0.0014 | 6.4E-07 | -0.0062 | 0.0017 | 2.1E-04 | 0.6498 |
| rs2928148 | *INO80* | A | 0.0055 | 0.0012 | 5.7E-06 | 0.0042 | 0.0014 | 0.0026 | 0.4808 |
|  |  |  |  |  |  |  |  |  |  |
| **Known loci**^[1,2]^ **possibly related to renal function** | | | | | | | | | |
| rs10109414 | *STC1* | T | -0.0069 | 0.0016 | 6.9E-06 | -0.0085 | 0.0020 | 4.2E-05 | 0.5316 |
| rs11959928 | *DAB2* | A | -0.0075 | 0.0016 | 1.5E-06 | -0.0123 | 0.0021 | 4.1E-09 | 0.0689 |
| rs12460876 | *SLC7A9* | T | -0.0103 | 0.0016 | 5.5E-11 | -0.0051 | 0.0021 | 0.0167 | 0.0488 |
| rs1260326 | *GCKR* | T | 0.0108 | 0.0016 | 3.8E-12 | 0.0061 | 0.0021 | 0.0035 | 0.0749 |
| rs12917707 | *UMOD* | T | 0.0155 | 0.0021 | 2.1E-14 | 0.0189 | 0.0027 | 3.1E-12 | 0.3225 |
| rs13538 | *ALMS1* | A | -0.0098 | 0.0019 | 1.4E-07 | -0.0088 | 0.0024 | 3.9E-04 | 0.7445 |
| rs1394125 | *UBE2Q2* | A | -0.0084 | 0.0018 | 9.0E-07 | -0.0113 | 0.0023 | 1.7E-06 | 0.3215 |
| rs17319721 | *SHROOM3* | A | -0.0124 | 0.0016 | 8.8E-16 | -0.0115 | 0.0020 | 3.1E-08 | 0.7249 |
| rs267734 | *LASS2* | T | -0.0116 | 0.0020 | 1.8E-09 | -0.0046 | 0.0026 | 0.0827 | 0.0336 |
| rs347685 | *TFDP2* | A | -0.0082 | 0.0017 | 1.2E-06 | -0.0078 | 0.0023 | 7.1E-04 | 0.8889 |
| rs4744712 | *PIP5K1B* | A | -0.0093 | 0.0016 | 1.9E-09 | -0.0068 | 0.0021 | 0.0013 | 0.3435 |
| rs626277 | *DACH1* | A | -0.0098 | 0.0016 | 4.3E-10 | -0.0049 | 0.0021 | 0.0236 | 0.0633 |
| rs6420094 | *SLC34A1* | A | 0.0105 | 0.0018 | 1.9E-09 | 0.0125 | 0.0024 | 5.4E-07 | 0.5060 |
| rs881858 | *VEGFA* | A | -0.0091 | 0.0019 | 5.0E-07 | -0.0113 | 0.0024 | 4.7E-06 | 0.4735 |
| rs7805747 | *PRKAG2* | A | -0.0109 | 0.0021 | 9.2E-08 | -0.0179 | 0.0032 | 1.1E-08 | 0.0633 |
|  |  |  |  |  |  |  |  |  |  |
| **Known loci**^[1,2]^ **suspected to be related to creatinine metabolism** | | | | | | | | | |
| rs10774021 | *SLC6A13* | T | -0.0078 | 0.0016 | 1.0E-06 | -0.0046 | 0.0021 | 0.0334 | 0.2253 |
| rs10794720 | *WDR37* | T | -0.0146 | 0.0029 | 4.9E-07 | -0.0090 | 0.0039 | 0.0181 | 0.2461 |
| rs2279463 | *SLC22A2* | A | 0.0129 | 0.0024 | 3.7E-08 | 0.0133 | 0.0032 | 2.9E-05 | 0.9193 |
| rs491567 | *WDR72* | A | -0.0104 | 0.0019 | 1.7E-08 | -0.0093 | 0.0025 | 2.1E-04 | 0.7270 |
| rs6465825 | *TMEM60* | T | 0.0081 | 0.0016 | 1.9E-07 | 0.0061 | 0.0020 | 0.0036 | 0.4342 |
| rs7422339 | *CPS1* | A | -0.0104 | 0.0018 | 2.8E-09 | -0.0083 | 0.0024 | 8.1E-04 | 0.4850 |
| rs9895661 | *BCAS3* | T | 0.0120 | 0.0022 | 1.7E-08 | 0.0035 | 0.0028 | 0.2184 | 0.0176 |
| rs2453533 | *GATM* | A | -0.0108 | 0.0016 | 8.9E-12 | -0.0156 | 0.0021 | 1.8E-13 | 0.0689 |

**Abbreviations:** Ref. All.: reference allele; RAF: reference allele frequency; SE: standard error.

References

1.     Kottgen A, Glazer NL, Dehghan A, Hwang SJ, Katz R, et al. (2009) Multiple loci associated with indices of renal function and chronic kidney disease. Nat Genet 41(6): 712-717.

2.     Kottgen A, Pattaro C, Boger CA, Fuchsberger C, Olden M, et al. (2010) New loci associated with kidney function and chronic kidney disease. Nat Genet 42(5): 376-384.
