# Supplementary material for: Genome-Wide Association and Functional Follow-Up Reveals New Loci for Kidney Function
Source: PLoS Genet. 2012 Mar 29;8(3):e1002584. doi: 10.1371/journal.pgen.1002584 (PMC3315455; doi:10.1371/journal.pgen.1002584)
Supplement: Table S20 — Effects of novel loci on the logarithm of urinary albumin-to-creatinine ratio (log(UACR)) in the overall sample and by diabetes and hypertension status. (DOC) [file pgen.1002584.s032.doc]

**Table S20.** Effects of novel loci on the logarithm of urinary albumin-to-creatinine ratio (log(UACR)) in the overall sample and by diabetes and hypertension status.*

| **Locus information** | | | **Overall sample**  **(N=31,069)** | | **No diabetes**  **(N=26,652)** | | **Diabetes**  **(N=2,554)** | | **No hypertension**  **(N=19,447)** | | **Hypertension**  **(N=11,431)** | |
| --- | --- | --- | --- | --- | --- | --- | --- | --- | --- | --- | --- | --- |
| **SNPID** | **Locus name** | **Ref. All. (RAF)** | **Effect(SE)** | ***P* value** | **Effect(SE)** | ***P* value** | **Effect(SE)** | ***P* value** | **Effect(SE)** | ***P* value** | **Effect(SE)** | ***P* value** |
| rs3925584 | *MPPED2* | T(0.54) | -0.0022(0.0089) | 0.8091 | -0.0083(0.0092) | 0.3727 | 0.0476(0.0435) | 0.2792 | -0.0165(0.0098) | 0.0938 | 0.0232(0.0170) | 0.1761 |
| rs6431731 | *DDX1* | T(0.54) | -0.0076(0.0248) | 0.7616 | 0.0100(0.0254) | 0.6973 | -0.1184(0.1334) | 0.3796 | -0.0149(0.0271) | 0.5848 | -0.0067(0.0488) | 0.8923 |
| rs11078903 | *CDK12* | A(0.70) | -0.0233(0.0112) | 0.0390 | -0.0149(0.0116) | 0.2057 | -0.1347(0.0542) | 0.0138 | -0.0307(0.0121) | 0.0117 | -0.0167(0.0222) | 0.4573 |
| rs12124078 | *CASP9* | A(0.59) | -0.0064(0.0097) | 0.5096 | -0.0069(0.0100) | 0.4959 | -0.0861(0.0482) | 0.0768 | -0.0019(0.0106) | 0.8610 | -0.0025(0.0187) | 0.8966 |
| rs2453580 | *SLC47A1* | T(0.76) | 0.0057(0.0108) | 0.6015 | 0.0078(0.0111) | 0.4859 | -0.0353(0.0517) | 0.4990 | 0.0006(0.0119) | 0.9570 | 0.0190(0.0204) | 0.3577 |
| rs2928148 | *INO80* | A(0.52) | 0.0152(0.0089) | 0.0910 | 0.0097(0.0092) | 0.3007 | 0.0594(0.0429) | 0.1703 | 0.0134(0.0098) | 0.1736 | 0.0213(0.0171) | 0.2177 |

*****Reference: Böger CA, Chen MH, Tin A, Olden M, Köttgen A, et al. (2011) CUBN is a gene locus for albuminuria. J Am Soc Nephrol 22(3): 555-570.

**Abbreviations:** Ref. All.: reference allele; RAF: reference allele frequency; SE: standard error.

**Note**: all *P* values reported in this table were corrected for inflation using the genomic-control method twice: at the individual-study level, before the meta-analysis, and after the meta-analysis on the summary results.
