# Supplementary material for: Genome-Wide Association and Functional Follow-Up Reveals New Loci for Kidney Function
Source: PLoS Genet. 2012 Mar 29;8(3):e1002584. doi: 10.1371/journal.pgen.1002584 (PMC3315455; doi:10.1371/journal.pgen.1002584)
Supplement: Table S21 — Effects (log odds ratios) of novel loci on microalbuminuria (MA) in the overall sample and by diabetes and hypertension status. (DOC) [file pgen.1002584.s033.doc]

**Table S21. Effects (log odds ratios) of novel loci on microalbuminuria (MA) in the overall sample and by diabetes and hypertension status.***

| **Locus information** | | | **Overall sample**  **(N=30,289)** | | **No diabetes**  **(N=25,914)** | | **Diabetes**  **(N=2,552)** | | **No hypertension**  **(N=18,501)** | | **Hypertension**  **(N=11,430)** | |
| --- | --- | --- | --- | --- | --- | --- | --- | --- | --- | --- | --- | --- |
| **SNPID** | **Locus name** | **Ref. All. (RAF)** | **Effect(SE)** | ***P* value** | **Effect(SE)** | ***P* value** | **Effect(SE)** | ***P* value** | **Effect(SE)** | ***P* value** | **Effect(SE)** | ***P* value** |
| rs3925584 | *MPPED2* | T(0.54) | 0.0003(0.0258) | 0.9915 | -0.0117(0.0296) | 0.6905 | 0.0605(0.0634) | 0.3395 | -0.0775(0.0406) | 0.0565 | 0.0492(0.0345) | 0.1535 |
| rs6431731 | *DDX1* | T(0.54) | -0.0470(0.0718) | 0.5125 | -0.0182(0.0813) | 0.8217 | -0.2141(0.1863) | 0.2504 | -0.0755(0.1094) | 0.4900 | -0.0323(0.0979) | 0.7417 |
| rs11078903 | *CDK12* | A(0.70) | -0.0415(0.0327) | 0.2042 | -0.0323(0.0378) | 0.3888 | -0.0931(0.0795) | 0.2414 | -0.0366(0.0507) | 0.4696 | -0.0457(0.0444) | 0.3030 |
| rs12124078 | *CASP9* | A(0.59) | 0.0129(0.0288) | 0.6548 | 0.0129(0.0330) | 0.6953 | -0.0646(0.0713) | 0.3645 | 0.0239(0.0443) | 0.5895 | 0.0149(0.0384) | 0.6978 |
| rs2453580 | *SLC47A1* | T(0.76) | -0.0152(0.0312) | 0.6263 | -0.0120(0.0352) | 0.7311 | -0.0693(0.0776) | 0.3713 | -0.0124(0.0484) | 0.7979 | -0.0190(0.0419) | 0.6502 |
| rs2928148 | *INO80* | A(0.52) | 0.0179(0.0264) | 0.4977 | 0.0121(0.0301) | 0.6872 | 0.0369(0.0648) | 0.5693 | 0.0313(0.0405) | 0.4393 | 0.0069(0.0353) | 0.8450 |

*****Reference**:** Böger CA, Chen MH, Tin A, Olden M, Köttgen A, et al. (2011) CUBN is a gene locus for albuminuria. J Am Soc Nephrol 22(3): 555-57**0.**

**Abbreviations:** Ref. All.: reference allele; RAF: reference allele frequency; SE: standard error.

**Note**: all *P* values reported in this table were corrected for inflation using the genomic-control method twice: at the individual-study level, before the meta-analysis, and after the meta-analysis on the summary results.
