# Supplementary material for: Genome-Wide Association and Functional Follow-Up Reveals New Loci for Kidney Function
Source: PLoS Genet. 2012 Mar 29;8(3):e1002584. doi: 10.1371/journal.pgen.1002584 (PMC3315455; doi:10.1371/journal.pgen.1002584)
Supplement: Table S22 — Association of novel loci with diastolic and systolic blood pressure in the ICBP consortium. (DOC) [file pgen.1002584.s034.doc]

**Table S22**. Association of novel loci with diastolic and systolic blood pressure in the ICBP consortium.*

| **Locus information** | | | **Diastolic blood pressure** | | | | | **Systolic blood pressure** | | | | |
| --- | --- | --- | --- | --- | --- | --- | --- | --- | --- | --- | --- | --- |
| **SNPID** | **Locus name** | **Ref. All.** | **RAF** | **Sample size** | **No. of studies** | **Effect(SE)** | ***P* value** | **RAF** | **Sample size** | **No. of studies** | **Effect(SE)** | ***P* value** |
| rs3925584 | *MPPED2* | T | 0.54 | 68,184 | 43 | 0.1177(0.0618) | 0.0567 | 0.54 | 68,193 | 43 | 0.1791(0.0974) | 0.0660 |
| rs6431731 | *DDX1* | T | 0.94 | 38,266 | 40 | 0.0100(0.1644) | 0.9516 | 0.94 | 38,113 | 40 | -0.0142(0.2606) | 0.9564 |
| rs11078903 | *CDK12* | A | 0.76 | 61,983 | 41 | -0.0046(0.0762) | 0.9522 | 0.76 | 61,969 | 41 | 0.0773(0.1212) | 0.5239 |
| rs12124078 | *CASP9* | A | 0.69 | 68,938 | 43 | -0.0632(0.0662) | 0.3398 | 0.69 | 68,950 | 43 | -0.1303(0.1040) | 0.2105 |
| rs2453580 | *SLC47A1* | T | 0.59 | 56,498 | 43 | -0.0089(0.0678) | 0.8957 | 0.59 | 56,507 | 43 | 0.0316(0.1072) | 0.7681 |
| rs2928148 | *INO80* | A | 0.53 | 69,146 | 43 | -0.1313(0.0614) | 0.0325 | 0.53 | 69,167 | 43 | -0.0919(0.0971) | 0.3441 |

*****Reference: The International Consortium for Blood Pressure Genome-Wide Association Studies, Ehret GB, Munroe PB, Rice KM, Bochud M, et al. (2011) Genetic variants in novel pathways influence blood pressure and cardiovascular disease risk. Nature 478(7367):103-109.

**Abbreviations:** Ref. All.: reference allele; RAF: reference allele frequency; SE: standard error.
