# Supplementary material for: Genome-Wide Association and Functional Follow-Up Reveals New Loci for Kidney Function
Source: PLoS Genet. 2012 Mar 29;8(3):e1002584. doi: 10.1371/journal.pgen.1002584 (PMC3315455; doi:10.1371/journal.pgen.1002584)
Supplement: Table S23 — Association of novel loci with myocardial infarction in the CARDIoGRAM consortium. (DOC) [file pgen.1002584.s035.doc]

**Supplementary Table 23. Association of novel loci^†^ with myocardial infarction in the CARDIoGRAM consortium.***

| **SNPID** | **Locus name** | **Reference allele** | **Reference allele frequency** | **Sample size** | **Odds ratio** | **95% confidence interval** | ***P* value** |
| --- | --- | --- | --- | --- | --- | --- | --- |
| rs3925584 | *MPPED2* | C | 0.44 | 82,472 | 1.02 | (0.99, 1.05) | 0.1550 |
| rs6431731 | *DDX1* | C | 0.06 | 72,946 | 0.96 | (0.90, 1.03) | 0.2856 |
| rs11078903 | *CDK12* | G | 0.22 | 77,474 | 1.01 | (0.98, 1.05) | 0.4634 |
| rs12124078 | *CASP9* | G | 0.32 | 82,860 | 1.01 | (0.98, 1.04) | 0.4496 |
| rs2928148 | *INO80* | G | 0.48 | 81,505 | 1.02 | (0.99, 1.05) | 0.1953 |

*Reference: Schunkert H, Konig IR, Kathiresan S, Reilly MP, Assimes TL, et al. (2011) Large-scale association analysis identifies 13 new susceptibility loci for coronary artery disease. Nat Genet 43(4): 333-338.

**^†^SNP rs2453580 did not pass CARDIoGRAM internal quality control checks and was not assessed.**
